# Recent trends of organic synthesis through cobalt metal-based catalysis: a review

**DOI:** 10.1039/d6ra04780g

**Published:** 2026-07-30

**Authors:** Sehrish Sewyra, Muhammad Zubair, Saima Bibi, Nighat Nawaz, Rehana Riaz, Faiz Ahmed

**Affiliations:** a Department of Chemistry, Government College University Faisalabad Pakistan zubairmkn@gcuf.edu.pk

## Abstract

Over the last three decades, transition-metal-catalysed organic transformations have been shown to be extremely important in organic synthesis. Cobalt, a first-row transition metal, has gained prominence due to its low toxicity, cost-effectiveness, and unique reactivity profiles, making it an attractive alternative to more precious metals traditionally used in catalysis. This review highlights the recent progress in the synthesis of biologically important organic compounds facilitated by cobalt metal-based catalysis, underscoring the versatility, and efficiency of these methodologies. In this review various cobalt-catalysed reactions like cycloaddition, cross coupling, multicomponent reaction, hydrogenation, reduction, hydroboration, amidation, annulation, isomerization and carbonylation reactions have been discussed since 2020, in which the products obtained have high yields with excellent enantioselectivities (ee). Future research in this area is poised to further expand the scope of cobalt-catalysed reactions, opening new avenues for the efficient and sustainable synthesis of biologically important compounds.

## Abstract 2

In this review various cobalt-catalysed reactions like cycloaddition, cross coupling, multicomponent reaction, hydrogenation, reduction, hydroboration, amidation, annulation, isomerization and carbonylation reactions have been discussed.

## Graphical abstract

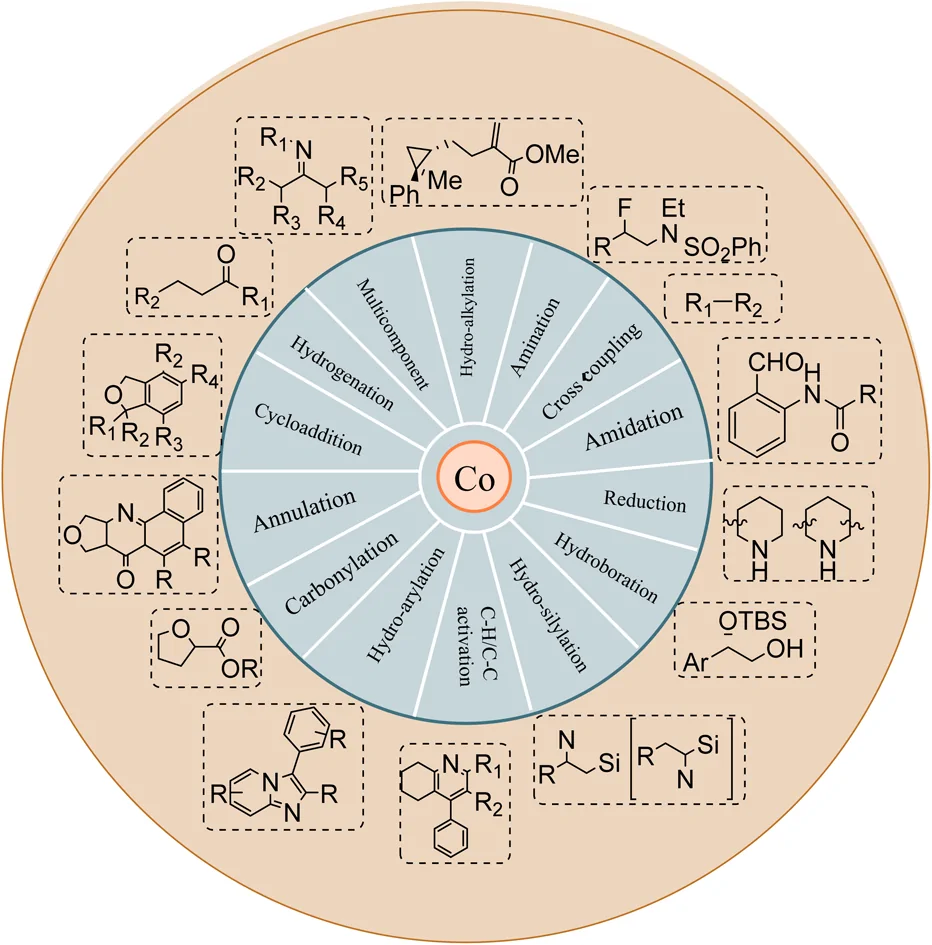

## 1 Introduction

**Fig. 1:**
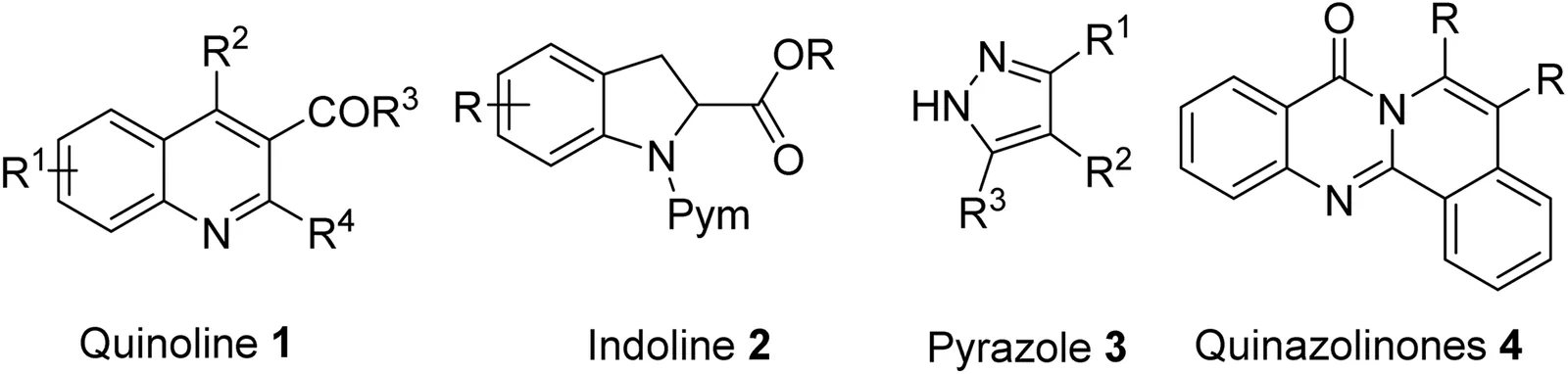
For biologically active compounds synthesized by cobalt-catalysis.

**Fig. 2:**
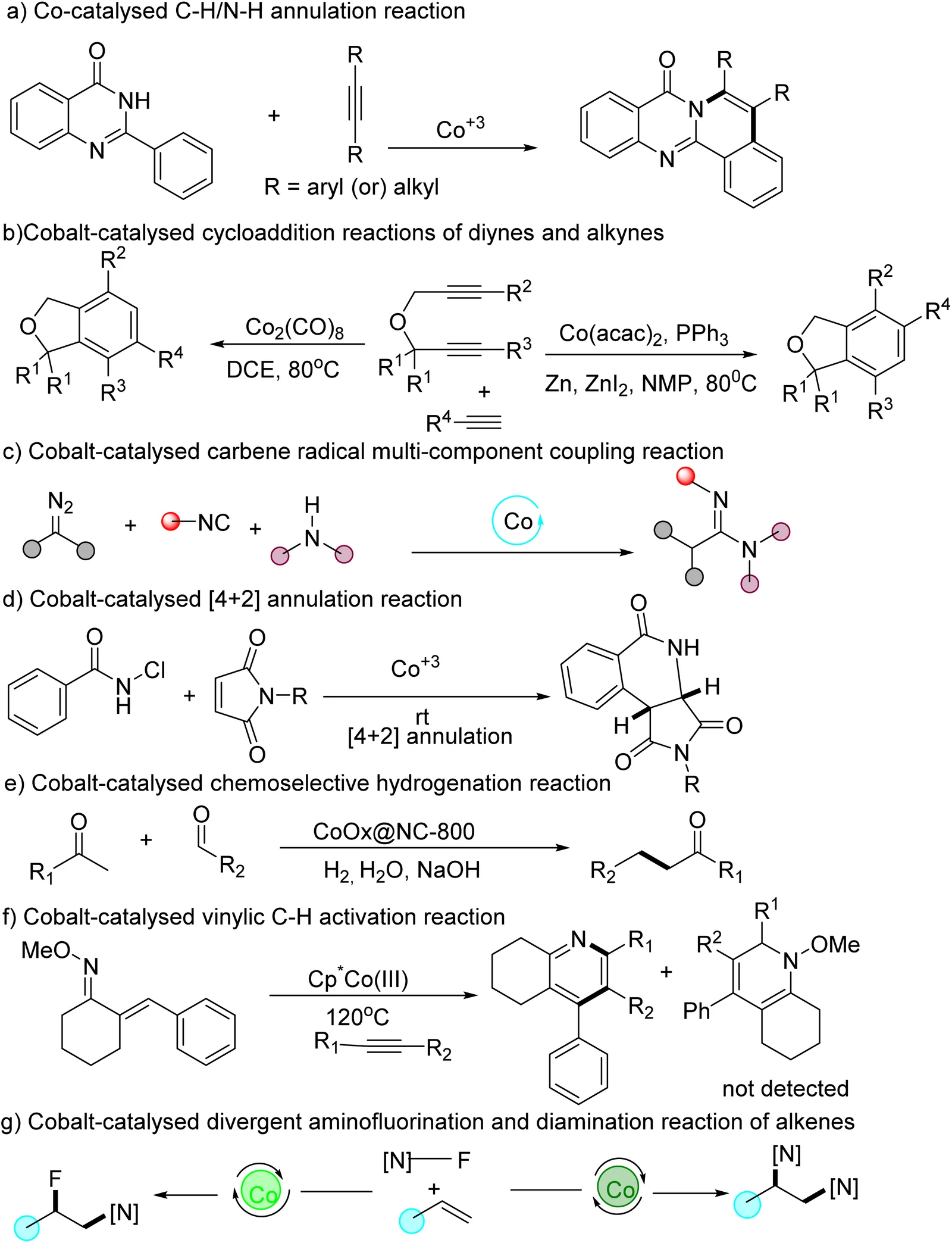
For various cobalt-catalysed reactions.

There is growing interest in the development of catalysts using first-row transition metals such as cobalt (Co), manganese (Mn), iron (Fe), nickel (Ni), and so forth.^1,2^ Among the first-row transition metals, cobalt is one of the most desirable options for catalysis. Cobalt metal was only formally recognized as an element in 1970. Cobalt metal-catalysed reactions have become more significant and have demonstrated notable growth in organic synthesis in recent years due to the progress made in organometallic chemistry. Fig. 1 shows the various biologically active compounds synthesized by cobalt-catalysis. These compounds show different biological activities like antibacterial, antitumor, antiviral analgesic, anticancer, antimalarial, antimicrobial, antifungal, anti-inflammatory, anti-diabetic, anti-tubercular, anti-hypertensive and other therapeutic effects. It is generally accepted that Co^+3^ hexamine was the initial coordination complex that Tassaert discovered in 1798 and cobalt complexes were also essential to Werner's revolutionary study in 1893.^3^ The majority of cobalt found in systems of living organisms as Co^+2^ and Co^+3^ though Co^+1^ (found in vitamin B12) and Co^+4^ are also familiar to exist.^4^ Cobalt is used in the chemical synthesis of medicines, other organic molecules and natural products. It is known that cobalt can catalyse a wide range of reactions, including hydro-functionalization,^5^ coupling reactions,^6^ cycloaddition reactions^7^ and hydrogenation^8^ as well as others (Fig. 2). The products of these reactions are biologically active and show different activities. For example, the product of a (Fig. 2) reaction is substituted quinazolinones, which is biologically active and shows various therapeutic effects. Conversely, transition metal catalysis has historically dominated organic synthesis^9^ and especially Co-catalysis is used in various transformations. Various processes that use cobalt metal as a catalyst are employed in order to produce bioactive compounds. Cobalt attached with different ligands and form various complexes. Remarkably, some of these cobalt complexes showed exceptional activity in catalytic hydrogenation reactions.^10,11^ Furthermore, it has been shown that these carefully constructed catalytic complex systems, which are created *in situ* from cobalt precursors and multidentate ligands (such as tetraphos and triphos) can catalyse significant hydrogenation reactions.^12^ Particularly, cobalt catalysts^13–26^ and nanostructured 3d-metal catalysts^25,27–29^ have all been developed especially for the *N*-heterocycles' selective hydrogenation. It was possible to find a very active cobalt catalyst for the specific hydrogenation of aromatic *N*, *O*, and *S* heterocycles.

Cobalt catalysis has wide range of biological history in the synthesis of natural products.^388–392^ It is used to synthesis complex compound sach as anti-tumor sesquiterpene coriolin and lycopodium alkaloids such as fawcettidine, fawcettimine and lycoflexin.^393^

Okamoto *et al.*, reported a regioselective [2 + 2 + 2] reaction when zinc powder and CoCl_2_·6H_2_O were present as the catalyst, cycloaddition of diynes and nitriles to produce substituted pyridines (a).^30^ Synthetic chemists have focused significant attention on developing cost-effective, efficient, and sustainable C–H activation catalysed by cobalt catalysts.^31–40^ In order to synthesize different *N*-methyl indolo[1,2-*a*]quinoxalin-6(5*H*)-ones, Xu and colleagues proposed in 2019 a C–H activation and *N*-dealkylative carbonylation of *o*-indolyl *N*,*N*-dimethylarylamines under a mixed gas of carbon monoxide and oxygen at a pressure of one atmosphere facilitated by copper.^41^ Cobalt-catalysed hydroalkenylation,^42^ hydrosilylation^43^ and NHC-catalysed hydroacylation^44,45^ have all been used for producing different functionalized cyclopropanes enantioselectively. In the absence of cobalt catalyst high temperature and pressure is required in mostly reactions used for the synthesis of various compounds. The less reactivity and enantioselectivity is obtained without atom-economical cobalt catalysis. The earthly abounding, economical cobalt catalysis^46^ underwent a redox-neutral radical transfer process with *N*-fluoroamides and *N*-fluorosulfonamides^47–52^ to form their corresponding intramolecular C–H amination and C–H fluorination products, respectively.^53^ Based on the constitutional radical-type reactivity of cobalt^+3^ carbene radicals, metalloradical catalysis provides a pathway to a particular class of eight membered ring compounds (dibenzocyclo octenes). Dihydropyridines have been reported to be produced by vinylic C–H activation of α,β-unsaturated imines with a relatively low valence cobalt catalyst followed by annulation with alkynes.^54,55^

Various research groups summarized the significant reactions and applications of cobalt catalysis, considering its synthetic utility. In 2024, Li *et al.*, group of researchers summarized the applications of cobalt catalysis in regio- or stereoselective hydro functionalization of alkenes and alkynes specifically.^56^ Jose & Mathew 2024 presented the cobalt mediated hydrosilylation across C–C, C–O and C–N multiple bonds.^57^ In 2024 Hassan *et al.*, summarized the cobalt-catalysed reductive coupling in detail.^58^

The catalytic systems align with green chemistry rules has accelerated because of environment friendly and sustainable chemical processes. Due to special electrical properties, high surface area to volume ratio and adaptable reactivity history, cobalt based nanoparticles have become important catalyst.^394–396^ Cobalt is a first-row transition metal. It is easily affordable because of its low price and safe substitute for valuable metals like palladium, platium, rhodium and ruthenium, which are restricted by their high price, scarcity and environmental issues.^397,398^

Because of magnetic behavior, reachable redox states and highly important capacity, cobalt nanoparticles as a heterogenous catalyst used. It is used in cascade reactions like C–H functionalization, hydrogenation and transfer hydrogenation reactions, cross coupling procedures and reductive aminations.^399–407^

Cobalt nanoparticles like bimetallic alloys, substrate-anchored nanocomposites and core–shell nanoparticles are prepared to enhance stability, selectivity and recyclability during catalytic cycles.^407^ Our review aims to address cobalt-catalysed important organic transformations in detail.

## 2 Literature review

### 2.1 Ligand controlled stereoselective reaction

The major class of carbohydrates known as 2-deoxy-β-*C*-glycosides is used extensively in the pharmaceutical industry and is found in many bioactive compounds.^59–61^*C*-glycosides' biological activity and biostability make them significant in the field of research.^62^*C*-glycosides' biological activity is greatly influenced by their structure. Because there are no substituents at the C-2 position, 2-deoxy-β-*C*-glycosides are highly challenging to synthesize stereo-selectively. However, a novel ligand-controlled method has been presented to build difficult 2-deoxy-β-*C*-glycoside from widely available glycals and alkyl-halides. In the presence of cobalt catalyst and ligand (L1) the β-configuration can be obtained with greater selectivity and effectiveness. Nevertheless, the study reports strong stereoselectivity in the hydroalkylation of glycals by Co-catalysed ligand-controlled hydroalkylation to obtain 2-deoxy-β-*C*-glycoside. In hydroalkylation mechanism, the halide portion of RX is coordinated with LCo^I^X eliminating the alkyl free radical and form LCo^II^X_2_ species. Then LCo^II^X_2_ react with silane and get hydride from it and form LCo^II^X-H species because Co–H moiety has key role in determining the β-selectivity. In next step LCo^II^X-H binds with C–C double bond of glycal and form adduct. The adduct rapidly react with alkyl free radical and undergoes reductive elimination. After reductive elimination the desired product (alkyl glycal) with β-selectivity is produced and original catalyst is regenerated. Cobalt demonstrates stereoselective, cost-effective and biologically compatible hydro-functionalization reactions.

**Scheme 1:**
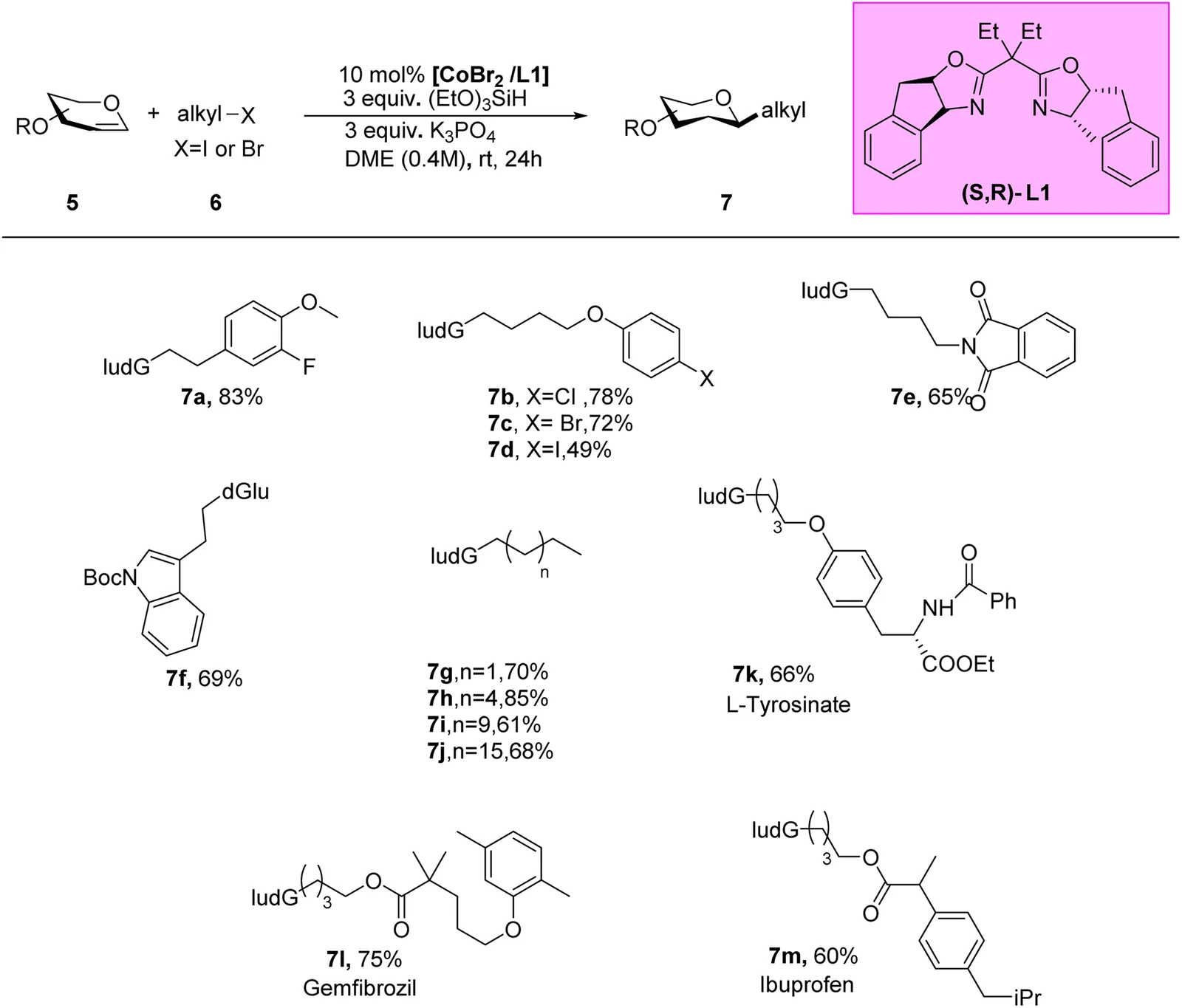
Concerning the 2-deoxy-β-*C*-glycosides that are catalysed by cobalt, DME = dimethyl ether, rt = room temperature.

The alkyl halides' 6 substrate scope was investigated by Liu *et al.*, under optimal reaction conditions (Scheme 1). To achieve high efficiency and stereoselectivity, the ligand (*R*,*S*)-L1 having high enantioselectivity was employed in the process. This transformation was possible for a large variety of alkyl halides, such as RI and R–Br facilitating to the synthesis of corresponding 2-deoxy-β-*C*-glycosides 7 in moderate to good yields with greater stereoselectivity. The efficiency of alkyl bromides' reaction was only slightly enhanced. Functional groups that were tolerated included amides 7e, heterocyclic groups 7f, –F(product 7a), –Cl(7b), –Br(7c) and –I(7d). For instance, the presence of strongly coordinative heteroatoms such as nitrogen and sulfur does not significantly deactivate the Co-bisoxazoline catalyst. Furthermore, it has been demonstrated that the catalyst rather than any specific groups in the RX coupling partners, controls stereoselectivity because nonfunctionalized alkyl halides are equally effective substrates for the conversion with good selectivity (products 7g–7j). The numerous 2-deoxy-β-*C*-glycoside derivatives of pharmacological importance and natural products, such as l-tyrosinate 7k, gemfibrozil 7l and ibuprofen 7m can also be effectively obtained using this method.

**Fig. 3:**
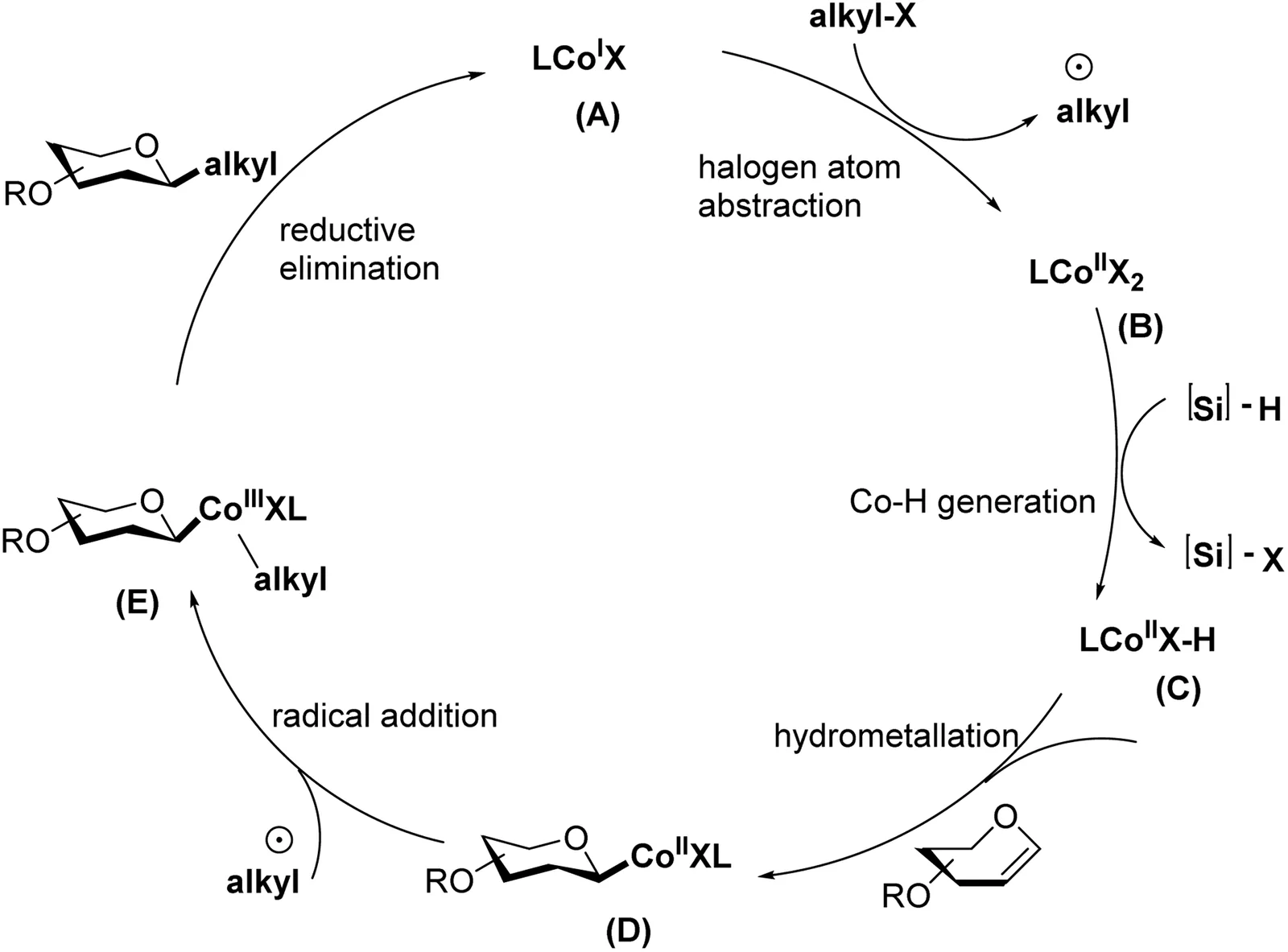
Proposed reaction mechanism.

Fig. 3 shows a hypothetical reaction mechanism that may have involved the activation of alkyl halides (A to B) the formation of Co^II^–H (B to C), the reductive elimination of Co^+3^ species and the *syn*-hydrometallation of Co^II^–H (C to D) to yield the intended products (E to A).^61^

### 2.2 Hydrogenation reactions

Numerous organic and synthetic bioactive substances contain *Z*-olefins as key structural elements. In certain cases, these compounds exhibit optical activity when chirality is present.^63,64^ Due to their lower thermodynamic stability than *E*-olefins, *Z*-olefins are more difficult to directly synthesize. By employing asymmetric sequential hydrogenation of conjugated enynes with a Ph-BPE-cobalt catalyst, an effective technique for producing chiral *Z*-allylamides with highly effectiveness and outstanding enantioselectivities (98.5–99.9% *ee*) has been devised. Due to their low cost and unique characteristics transition metal catalysts which are common in the earth's crust are extremely beneficial in asymmetric hydrogenation.

**Scheme 2:**
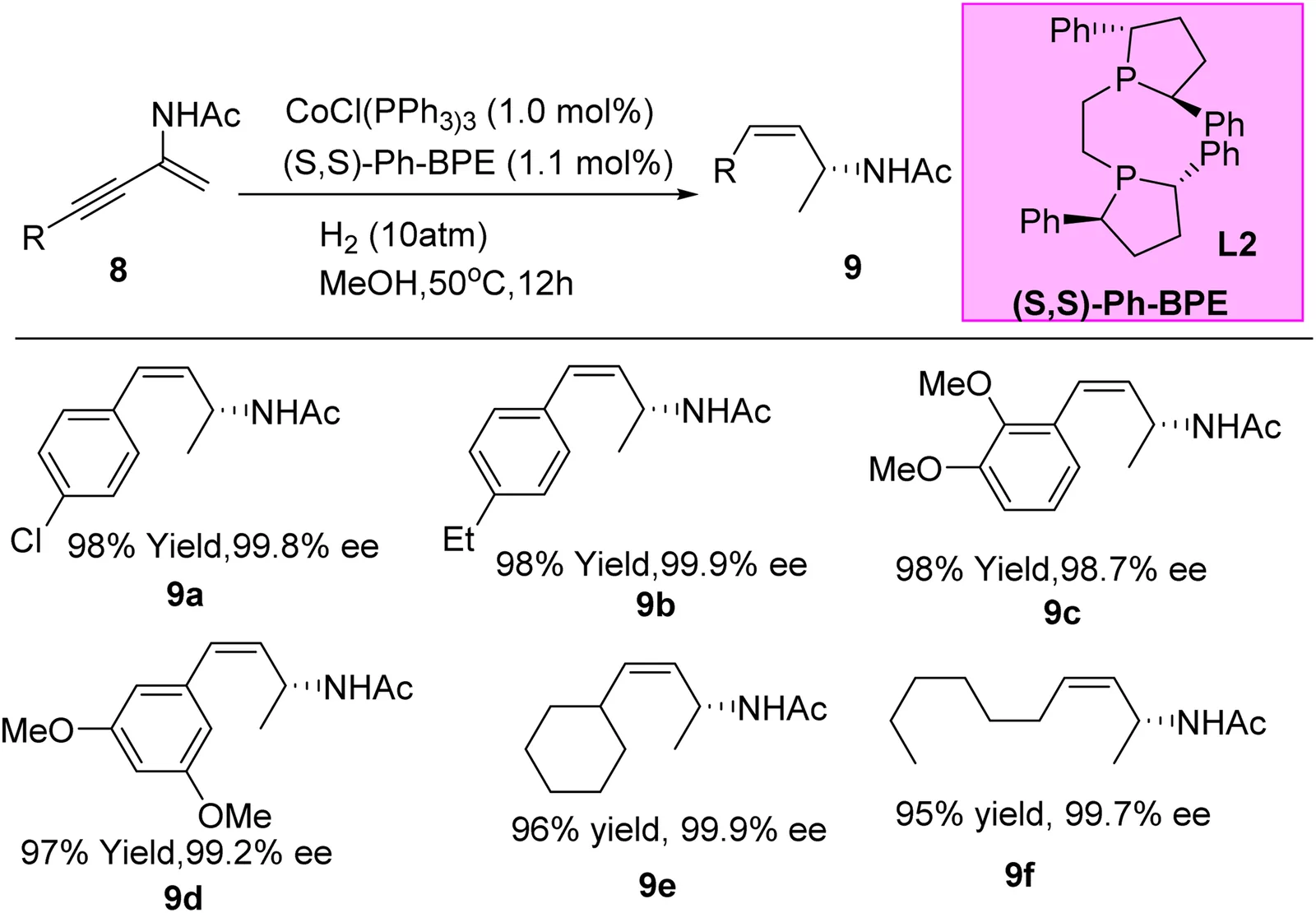
For the synthesis of chiral *Z*-allylamides.

More C2-linked diphosphine ligands were preferred by Allard *et al.*, under optimum reaction conditions because they are more efficient in their reactions than ligands that are linked at the position 3 and 4 of carbon. Although its phenyl-substituted counterpart (*S*,*S*)-phenyl substituted bis(phospholane) ethane showed remarkably excellent selectivity and activity (100%). The ligand that is similar to it the (*R*,*R*)-methyl substituted bis(phospholane) likewise provides substantially poor conversion. Monosubstituted enyne substrates bearing aliphatic and aromatic groups can be hydrogenated precisely to the appropriate products 9a and 9b under optimal reaction conditions, regardless of their pattern of substitution or electronic characteristics, with high yields (98% and 98%) and enantioselectivities. The related products 9c and 9d of the disubstituted substrates are also formed under various reaction conditions give 98% and 97% yield. Aryl groups were converted to alkyl groups, and the hydrogenation process went without difficulty, producing the required products 9e and 9f with high yield (96% and 97%) and excellent enantioselectivity (Scheme 2).^64^

**Fig. 4:**
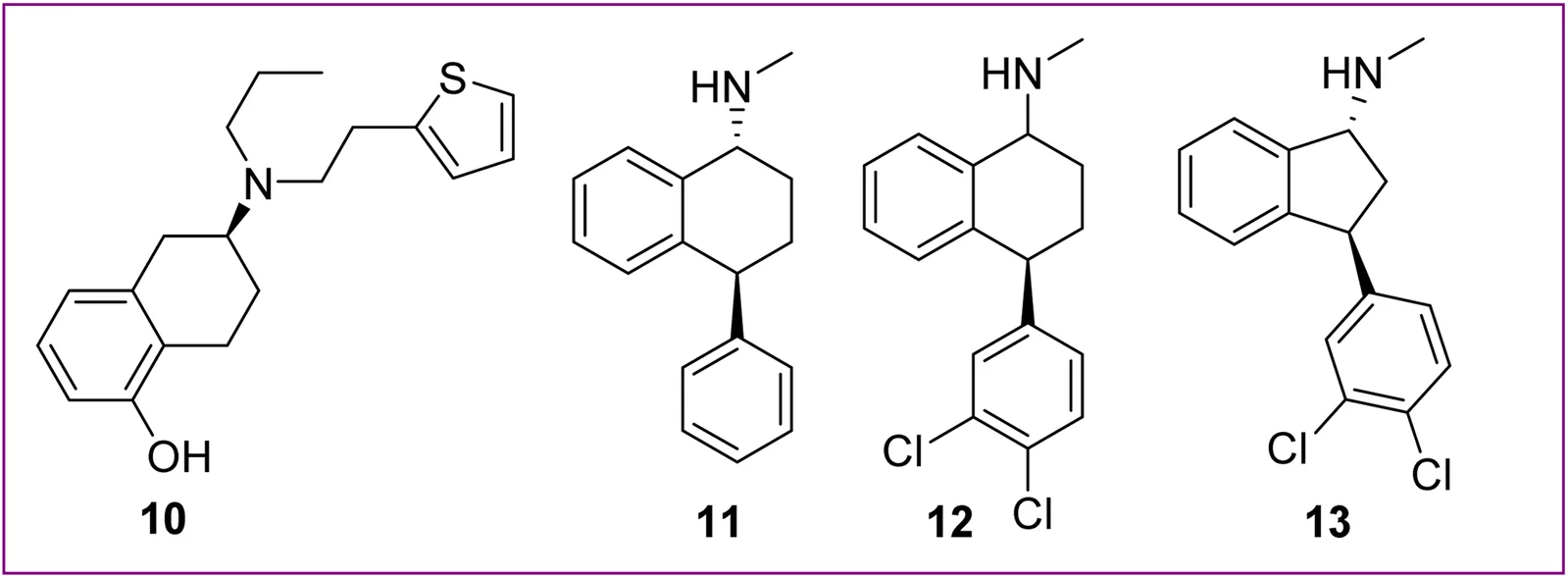
Different cyclic chiral amines having therapeutic properties.

One of the most atom-efficient approaches to constructing enantiopure saturated motifs from their prochiral precursors is enantioselective hydrogenation.^65^ Massive production of important medications and agricultural products is accomplished through the use of asymmetric hydrogenation.^65–71^ Enantiopure carboxylic motifs such chiral cyclic alkanes, amines, amides, esters and ethers *etc.* are present in many significant medications and a variety of bioactive compounds.^72^ The therapeutic properties of this class of olefins analogous chiral amine derivatives, such as rotigotine 10 (a dopamine agonist used to treat Parkinson's disease),^73^ tametraline 11 (a selective serotonin reuptake inhibitor),^74^ sertraline 12 (an antidepressant, selective serotonin reuptake inhibitor)^75^ and indatraline 13 (an antidepressive agent)^76^ make their asymmetric hydrogenation highly desirable (Fig. 4).

**Scheme 3:**
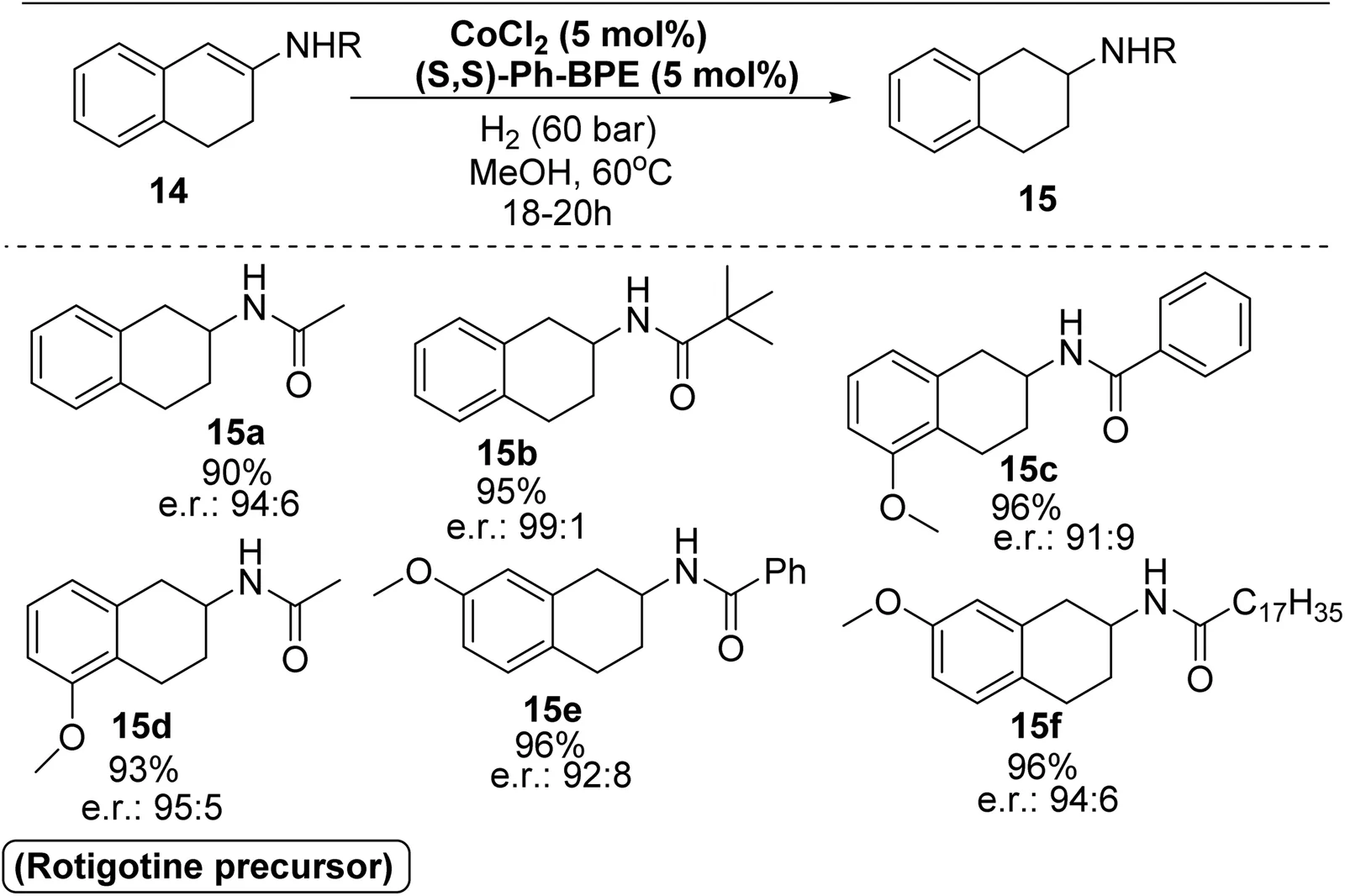
For the formation of cyclic amides with chirality.

Chakrabortty *et al.*, studied the general reaction's substrate range under optimum reaction conditions. *N*-(3,4-dihydronaphthalen-2-yl)acetamide is hydrogenated to produce amide 15a in 88% of the *ee* with 90% of the isolated yield. The hydrogenation continues with even greater enantioselectivity when the alkyl groups at the amide are changed to –Me and –^*t*^Bu, producing the corresponding product 15b. The product 15b is formed in great yield 95% when bulky groups such as *tert*-butyl are used in the amide functionality. These bulky groups increased enantioselectivity up to 98%. In 90% enantiomeric excess, enamide (5-OMe) was hydrogenated to produce 15d with 93%, a possible precursor for the production of rotigotine. Benzamide underwent hydrogenation to form 15c with 96% (82% *ee*). The methoxy derivative 15e (7-OMe, phenyl) is successfully hydrogenated to produce yield of 96% and enantioselectivity up to 99% *ee*. The corresponding stearic acid derivative's enamide 15f with yield 96%, which has a lengthy alkyl chain was also hydrogenated with 88% *ee* (Scheme 3).^77^

**Fig. 5:**
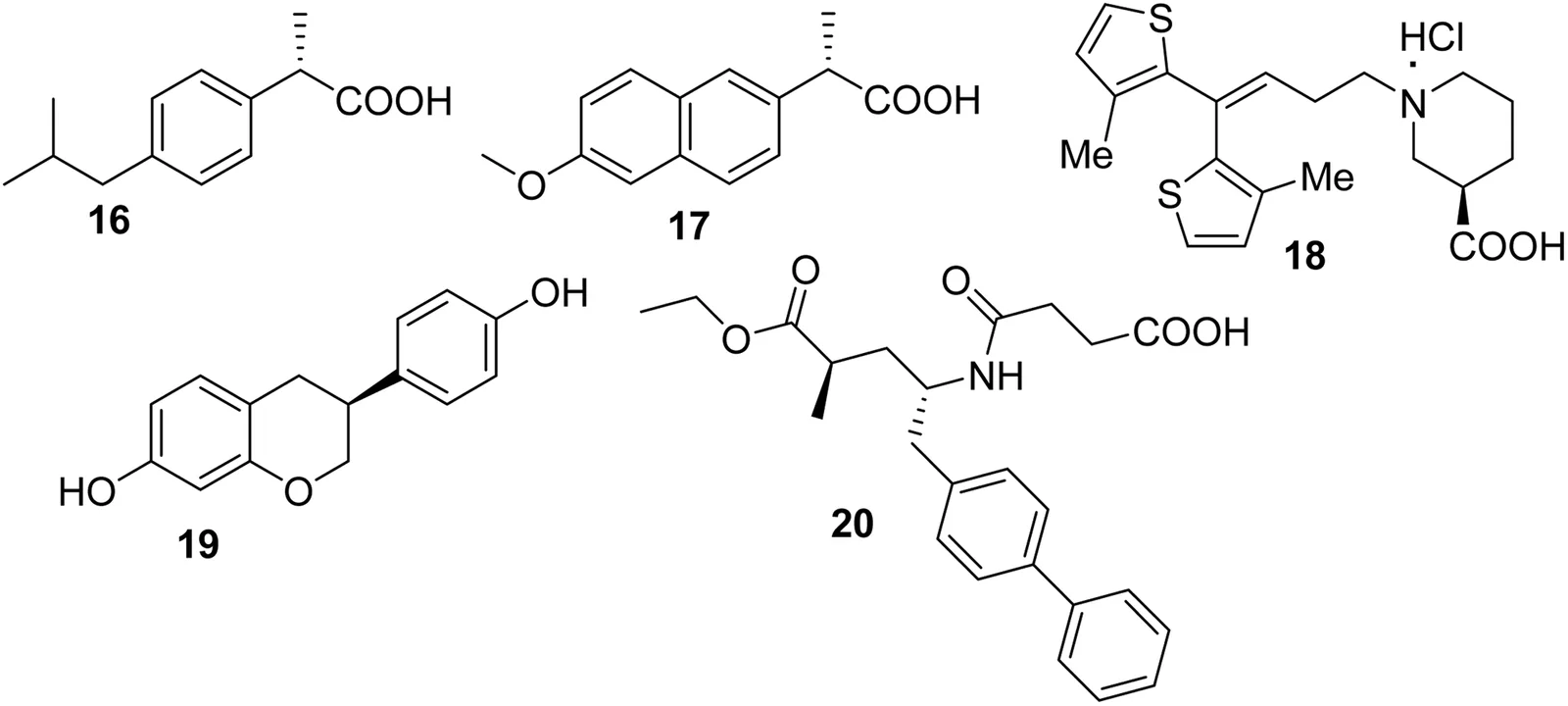
Chiral carboxylic acid-derived substances and medicines with biological activity.

The chiral carboxylic acids are important structural elements in a variety of medicinal compounds, agrochemicals, flavours and fragrances. In Fig. 5 chiral carboxylic acids are well-known medications like ibuprofen 16, naproxen^78,79^17 and (*R*)-tiagabine 18.^80^ Chiral carboxylic acids are vital intermediates in a significant number of pharmaceuticals or biologically active substances including (*S*)-equol 19 (ref. 81) and sacubitril 20.^82–84^ Enantioselective hydrogenation of α,β-unsaturated carboxylic acids using a transition metal catalyst is among the most efficient and economical techniques for producing chiral carboxylic acids.^85^

**Scheme 4:**
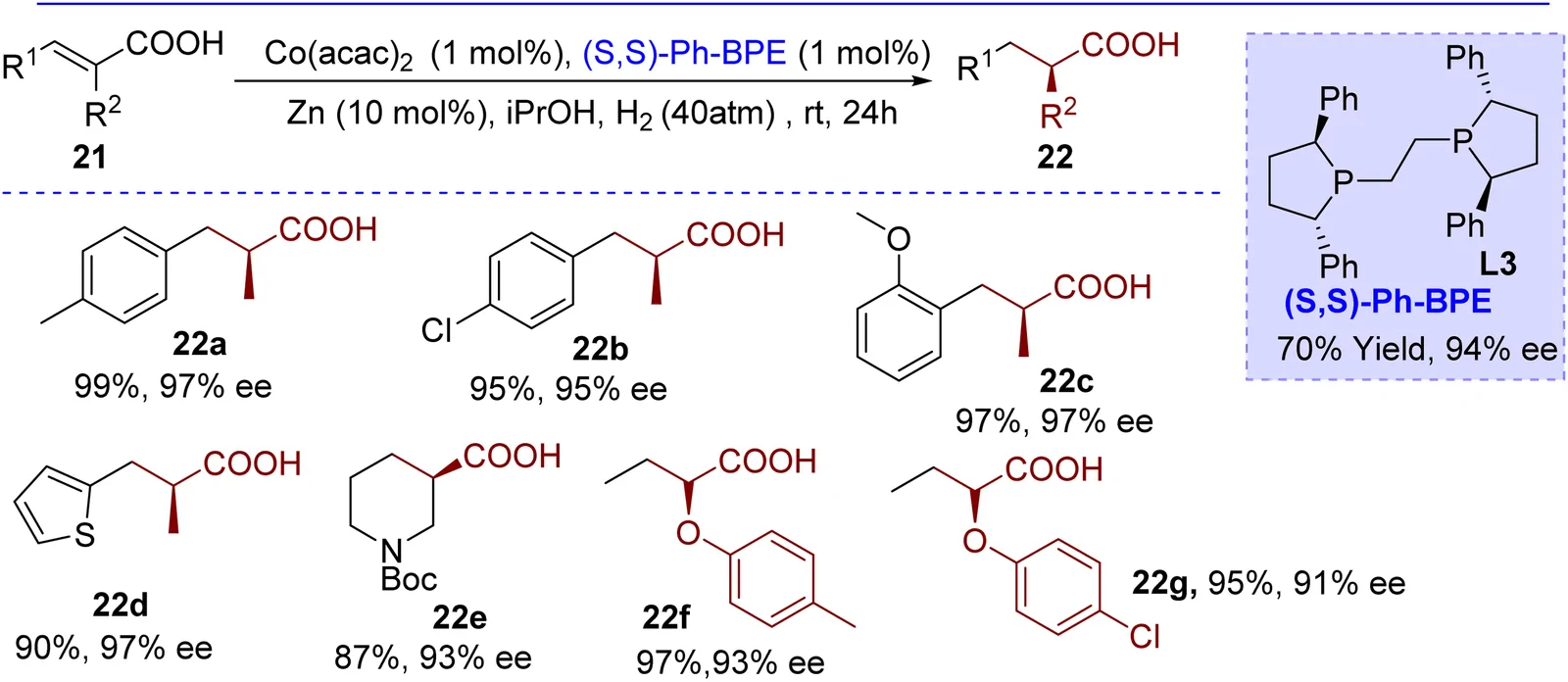
The method for hydrogenating α,β-unsaturated carboxylic acids.

To explain the scope of the cobalt-catalysed asymmetric hydrogenation Du *et al.*, subjected the catalytic mechanism for the reaction of several types of α,β-unsaturated carboxylic acids. The α-aryl and α-alkyl cinnamic acid derivatives were successfully hydrogenated to provide their corresponding products under optimal reaction conditions (Scheme 4). Most reactions proceeded smoothly at room temperature and 40 atm H_2_ pressure, producing the required products with high isolated yields and excellent enantioselectivities (95–99% *ee*). This process effectively produces the corresponding compounds (22a yield 95%, 22b yield 99%) for derivatives of α-methyl cinnamic acid having both electron-donating and electron-withdrawing groups. Under reaction conditions, substitutes at the *p*- and *m*-positions of the –Ph ring are tolerable and produce the hydrogenation product 22c in high yields 97% with excellent enantioselectivity 99% *ee*. Effective hydrogenation of heteroaromatic, -unsaturated carboxylic acid also produced the desired product 22d in 90% isolated yield with 97% *ee*. *N*-Boc-1,2,5,6-tetrahydropyridine-3-carboxylic acid underwent asymmetric hydrogenation to produce the target product 22e 87% with a remarkable 93% *ee*. Due to the fact that the related hydrogenation products are essential elements for the synthesis in both the pharmaceutical and agrochemical sectors, oxy-functionalized α,β-unsaturated carboxylic acids were also treated to [Co]/BPE. A wide variety of α,β-unsaturated carboxylic acids with α-aryloxy and α-alkoxy substitutes were smoothly hydrogenated to the corresponding chiral α-oxy-functionalized acids (22f 97% and 22g 95%) with excellent yield and enantioselectivities.^80^

The process of converting arenes into saturated cyclic compounds holds significant value in the manufacturing of refined and the majority of chemicals, pharmaceuticals, and agrochemicals.^86^ Arenes can be chemically hydrogenated, which is an advanced method to store hydrogen that is important in the synthesis of organic compounds. The development of nanostructured earth abundant metal catalysts^87–89^ which carry out significant chemical processes with greater selectivity as well as effectiveness has allowed for the selective hydrogenation of *N*-heterocycles. Selective hydrogenation of different classes of *N*-, *O*- and *S*-heterocyclic compounds such pyrroles, benzofuranes, aciridine amine indoles and isoquinoline could be produced using a highly active Co-catalyst under mild conditions.

**Scheme 5:**
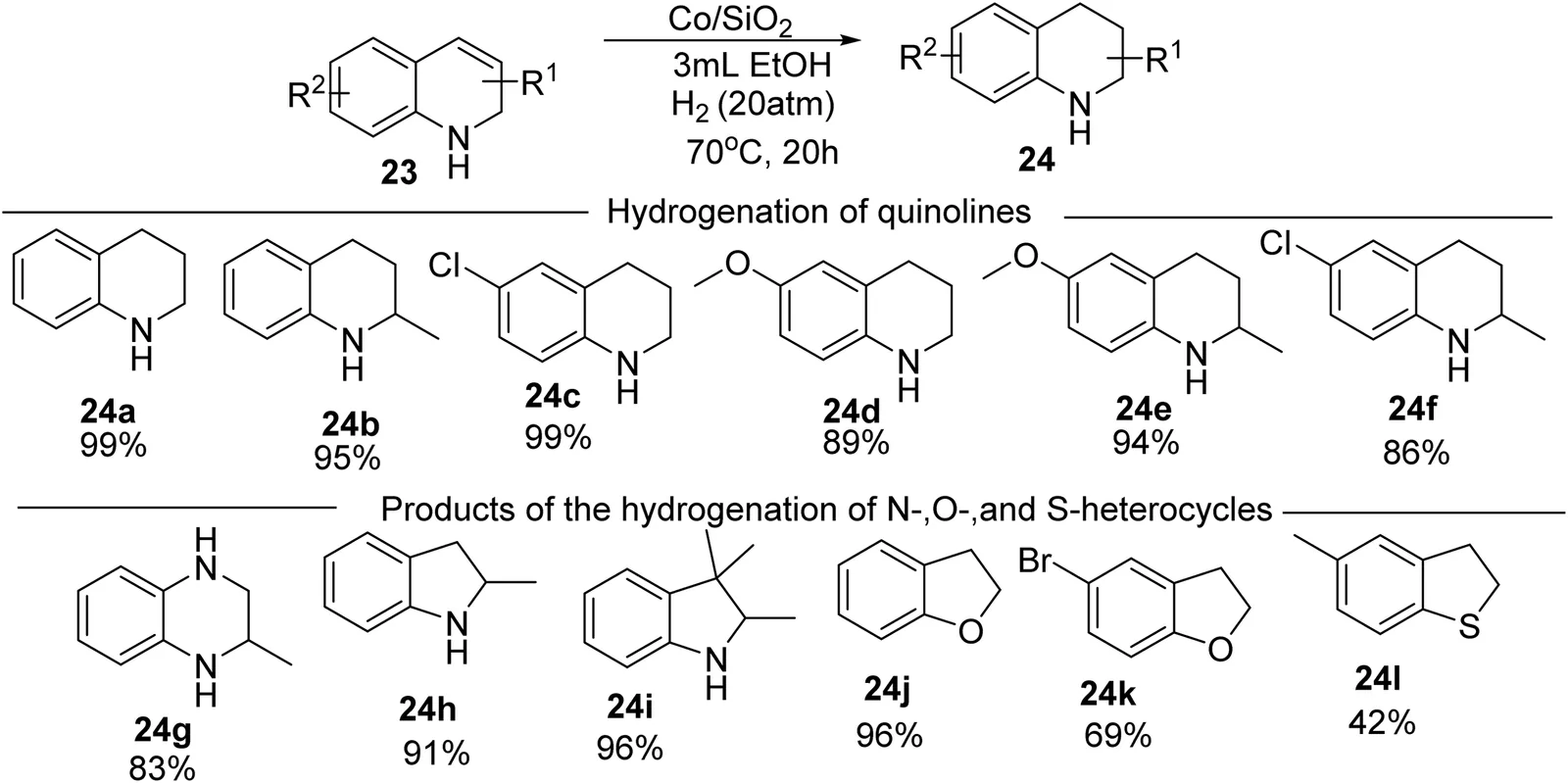
For selective hydrogenation of aromatic heterocycles.

Bauer *et al.*, investigated the substrate range of the catalyst has been described for optimal reaction conditions. Under extremely mild conditions, a number of functionalized and substituted quinolones go through selective hydrogenation to provide excellent yields of aliphatic cyclic derivatives. First, researchers used their catalyst to hydrogenate pure quinoline and the yield was very good. With up to 99% isolated yields, 1,2,3,4-tetrahydroquinolines were produced by mildly hydrogenating the different quinoline derivatives. Particularly, the electron-donating methyl substitutes and the electron-withdrawing halogen substitute both provide the desired product with a high yield (Scheme 5, product 24b 95% and 24c 99%). The product 24d was obtained 89% in (Scheme 5) when a methoxy group was present. Two functional groups (Scheme 5, products 24e 94% and 24f 86%) which produce that compounds in excellent yields can be tolerated by the cobalt catalyst. The 1,2,3,4-tetrahydroquinolines are employed as starting materials in the production of bioactive compounds. To be more specific, the products 24a, 24b, and 24d (Scheme 5) can lead to 5-HT3 receptor antagonists, antitrypanosomal medications, and tubulin polymerization inhibitors. After quinolines were selectively hydrogenated, they developed an interest in hydrogenating other heterocycles. For the first time using a nanostructured cobalt catalyst under mild reaction conditions, 2-methyl-1,2,3,4-tetrahydroquinoxaline (Scheme 5), product 24g 83%, was produced by the cobalt catalyst being used under reaction conditions. When 2-methylindole and 2,3,3-trimethylindolenin were hydrogenated, the appropriate product is produced in excellent yields (24h 91% and 24i 96%). It is advantageous to synthesize indolines because they are well-known as key agrochemicals and medicines. The catalyst was further being used for the more difficult hydrogenation of oxygen and sulphur heterocycles since the resulting compounds serve as important bioactive compounds. Benzofuran and 5-bromobenzofuran are selectively hydrogenated to produce products 24j 96% and 24k 69%, respectively. Although it is highly challenging to selectively hydrogenate benzothiophenes, but this can be done by using cobalt catalyst being used under reaction conditions (Scheme 5, product 24l 42%).^90^

**Fig. 6:**
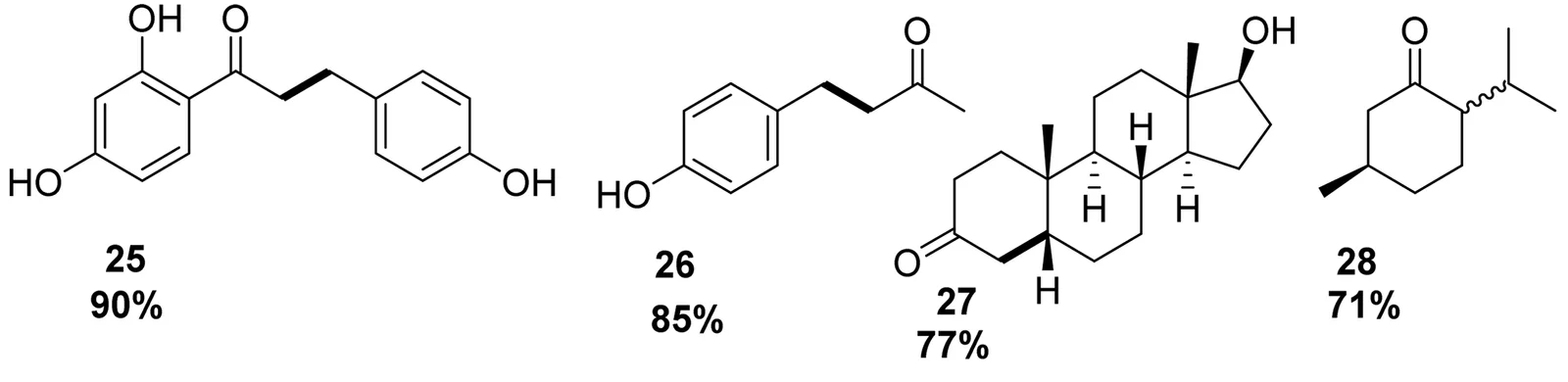
The 1,4-diaryl ketone skeletons were present in particular pharmaceutically useful compounds.

The process of hydrogenating α,β-unsaturated carbonyls chemo-selectively is an appropriate technique for the production of high-quality compounds, medicines, and functional materials.^91–95^ One significant synthetic process is the selective hydrogenation of α,β-unsaturated carbonyls' C=C bonds while leaving the C=O bond intact since the resulting reduced products are useful sub-structures for a variety of pharmaceutically active compounds.^96–99^ Under the mild reaction conditions CoO_*x*_@NC-800 (10 mol% of Co) catalyst, and in the presence of tetrabutylammonium iodide, 2 MPa H_2_, NaOH, the naturally occurring and/or biologically active compounds, including davidigenin 25, rheosmin 26, menthone 27 and testosterone 28, could also be effectively and produced in good to high yields through chemoselective synthesis from their corresponding α,β-unsaturated carbonyls (Fig. 6). The *cis*-isomer of testosterone was the only product of the testosterone's exclusive diastereoselective synthesis in the presence of a catalytic system.

**Scheme 6:**
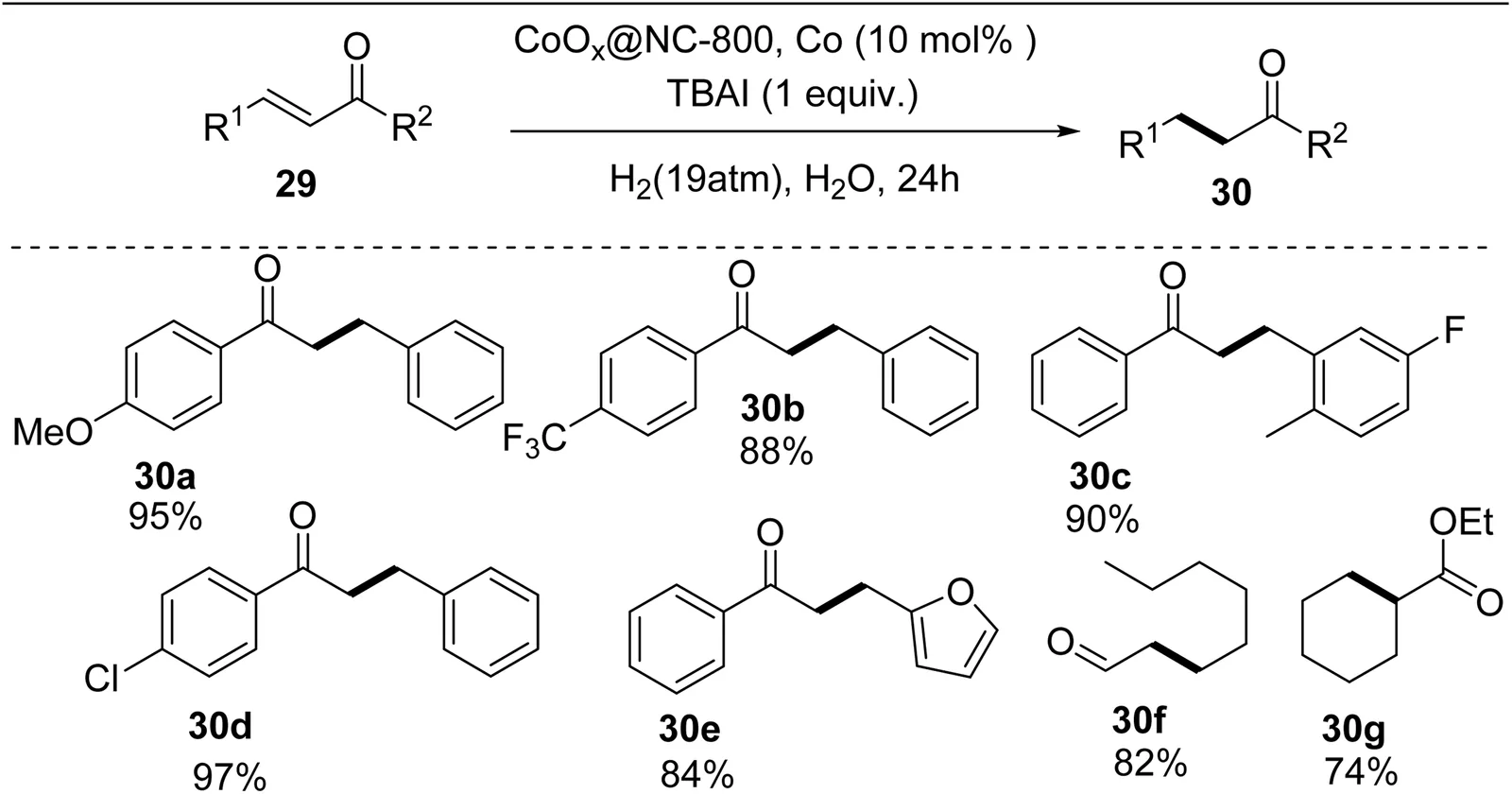
For α,β-unsaturated carbonyls that have been hydrogenated, TBAI = tetra-*n*-butylammonium iodide.

Song *et al.*, under idealized reaction conditions studied the various α,β-unsaturated carbonyls (Scheme 6). The required products 30a, 30b and 30c were produced in good to exceptional yields 95%, 88%, 90% by successfully reducing both electron-donating and electron-withdrawing aromatic substituted α, β-enones. When it comes to react, the steric effects are barely noticeable, notably for *ortho*-substituted. When *ortho* position is substituted in these compounds 30c such as the methyl group is particularly not very reactive with respect to steric effects. High isolated yields of the respective saturated carbonyls were generated by the halogen-substituted chalcones 30c 90% and 30d 97% without the presence of any by-products of dehalogenation. Heteroaromatic chacone-type derivatives worked well under the current conditions and produced the required saturated ketone 30e with an 84% yield. Furthermore, octanal, an aliphatic unsaturated aldehyde, was solely reduced to its corresponding saturated aldehyde 30f with an 82% yield. Ester, another aliphatic unsaturated carbonyl was easily transformed into the saturated carbonyls 30g with 74% yield.^100^ The biologically active compounds that are produced with cobalt catalyst exhibit excellent enantioselectivity and high to excellent yield. Different compounds can be hydrogenated under mild reaction conditions. Cobalt has potential benefits for the environment in addition to economic ones.

**Fig. 7:**
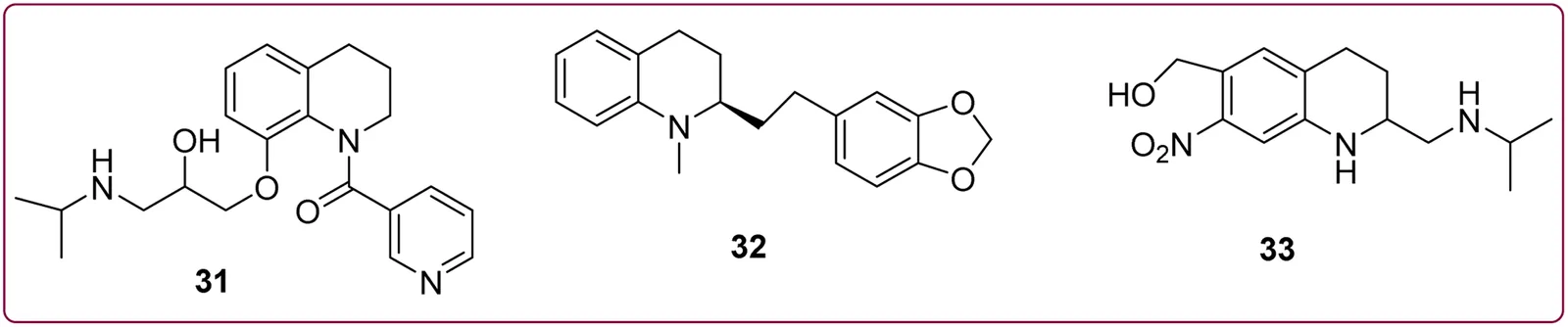
Tetrahydroquinoline-structured pharmaceuticals and organic compounds.

Numerous organic compounds and pharmaceutical compounds, including the antiarrhythmic drug nicainoprol 31, the antitischistosomiasis drug oxamniquine 33 and naturally occurring compounds like (*R*)-galipinine 32 (Fig. 7), contain 1,2,3,4-tetrahydroquinoline (THF) derivatives which are very significant *N*-heterocyclic compounds with various biological and pharmacological activities. Therefore, chemists are quite interested in synthesizing this molecule.^101,102^ Using ammonia and borane as hydrogen sources, core–shell Co-catalysts have been found to be more effective and selective in the manufacture of various medications and the transformation of biologically active compounds.

**Scheme 7:**
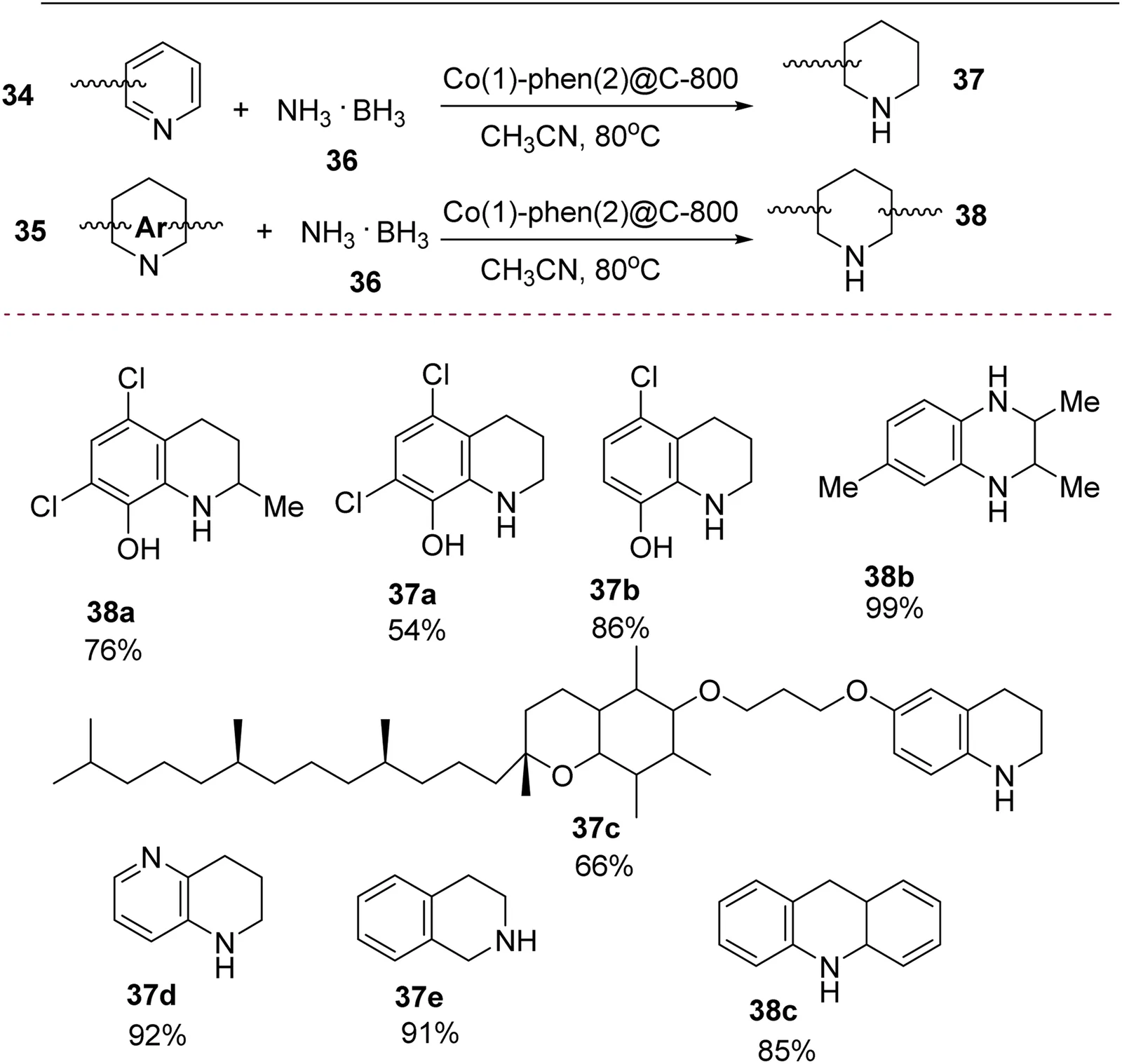
For the chemoselective reduction of *N*-heteroarenes.

The numerous bioactive quinoline derivatives have been researched under optimal reaction conditions (Scheme 7). Many bioactive molecules have the quinoline as essential structural components. A family of substituted quinolin-8-ols, including chlorquinaldol, chloroxine and cloxyquin 38a, 37a, and 37b, were produced 76%, 54%, 86% under ideal reaction conditions and demonstrated antibacterial, antifungal and antimicrobial properties. Vitamin E is combined with the quinoline substrate in order to yield the appropriate hydrogenated product 38b 99%. Other *N*-heteroarenes, such as 2,3,6-trimethylquinoxalin can be effectively reduced in this process in order to provide its equivalent tetrahydroquinoxaline 37c. With a 92% yield, the aromatic core of 1,5-naphthyridine is selectively hydrogenated, and the resulting product 37d is a basic component of the topoisomerase I inhibitor. With a 91% yield, the corresponding product 37e was produced by selectively reducing the other pharmacologically active molecule, isoquinoline. The target product 38c which is produced 85% by reducing trinuclear *N*-heteroarenes like acridine is obtained.^103^

High selectivity and efficacy are achieved in the reduction reactions when economical cobalt catalysis is used. Its eco-friendly, waste-free and effective methodology has a beneficial effect on the environment. The limitation of this reaction is that it requires high pressure and temperature in the absence of a metal catalyst. The use of metal catalysts such as Pd, Ir, Cu, and Pt in reduction processes is limited due to their non-atom-economic nature.

### 2.3 Cycloaddition reactions

**Fig. 8:**
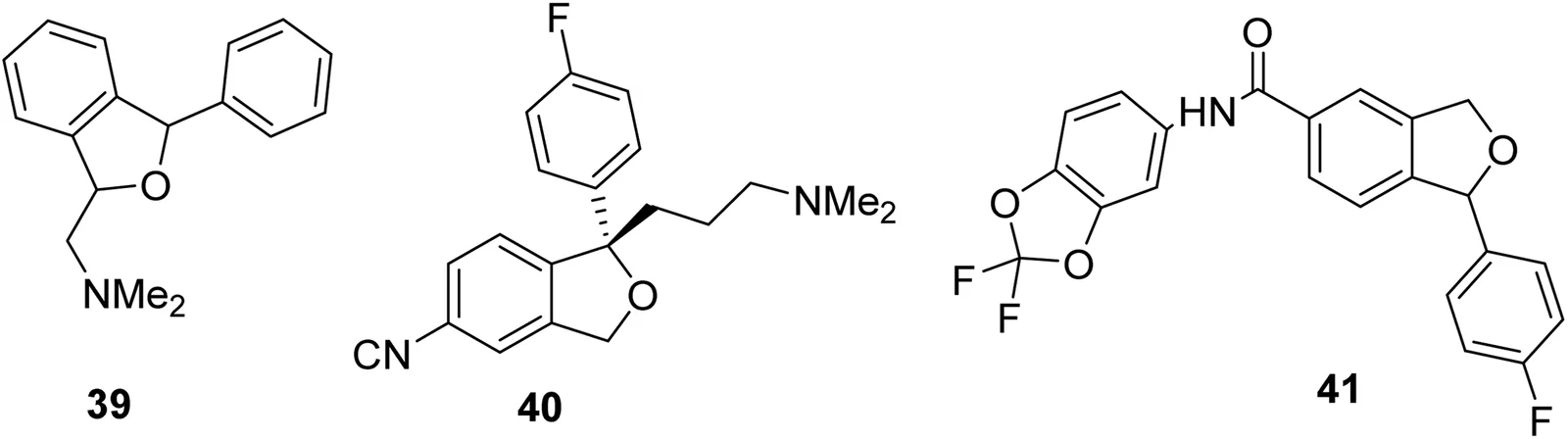
Various biologically active substances exhibiting a range of activities.

Major depression or MDD, is treated with escitalopram 40, a drug that inhibits the absorption of serotonin that contains 1,3-dihyroisobenzofurans, important scaffolds present in many natural products, medications, and compounds that are bioactive.^29,104,105^ There is strong anti-histaminic activity in compound 39. Compound 41 is a tumor fighting drug with potent anti-proliferative effects against prostate carcinoma cell lines (Fig. 8).^29^ In synthetic chemistry, 1,3-dihydroisobenzofuran products are useful starting points for the formation of functioning heterocyclic compounds.^106,107^ Because of the significance of their scaffolds, Wang *et al.*,^29^ used cobalt-catalysed cyclic addition of diynes and alkynes to produce 1,3-dihydroisobenzofurans.

**Scheme 8:**
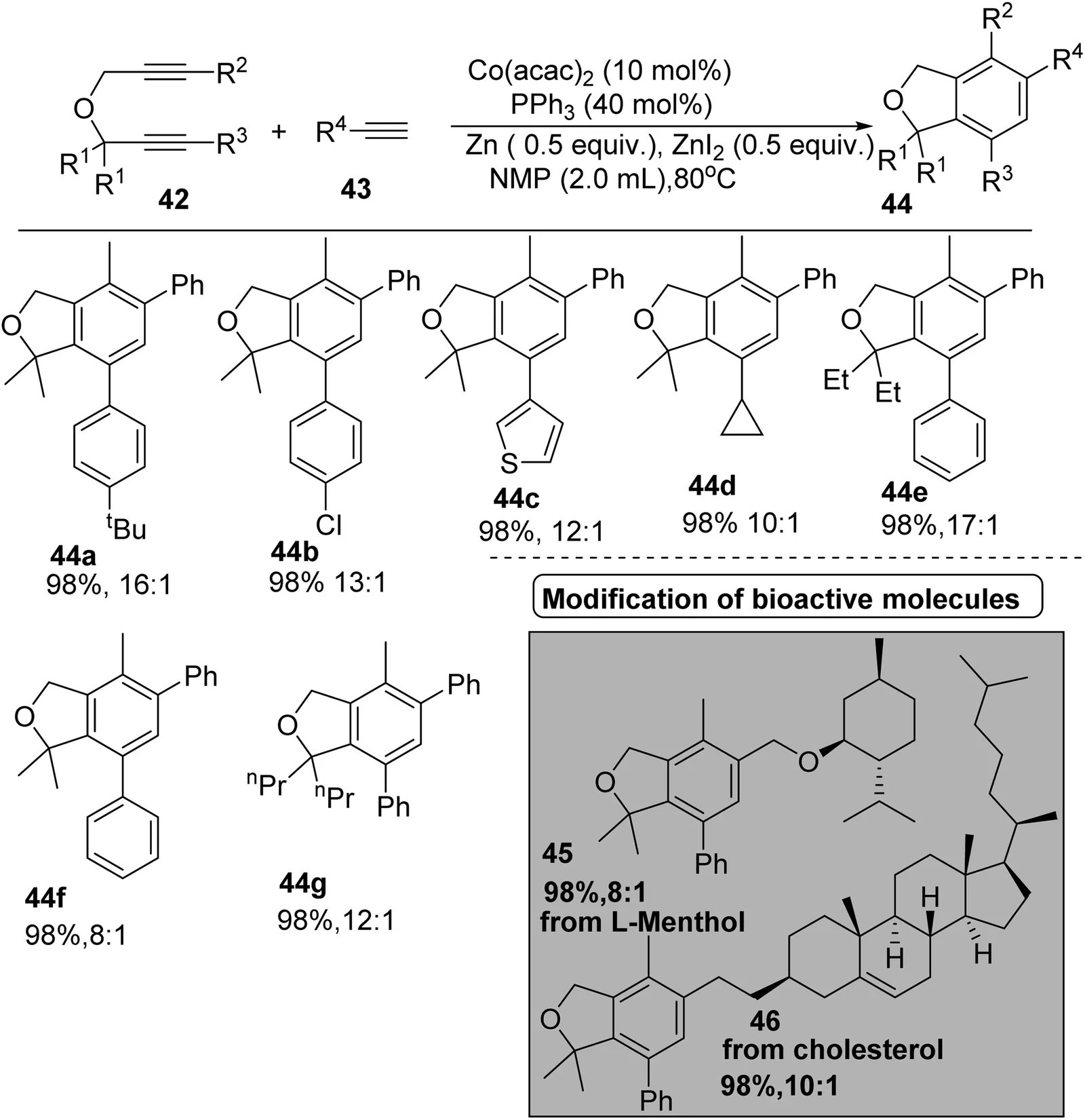
For synthesis of different derivatives of 1,3-dihydroisobenzofurans, NMP = *N*-methyl pyrrolidone.

The substrate scope for Co-catalysed cycloaddition was examined by Wang *et al.*, under the optimum reaction conditions. A variety of diynes have been evaluated when subjected to an alkyne (Scheme 8). For diynes that are *para*- or *meta*-substituted, the reaction produced the desired products 44a 98% and 44b 98%, but *meta*-substituted diynes showed less regioselectivity (16 : 1, 13 : 1). The desired products 44c and 44d were also effectively produced (98% and 98%) yield by the reaction with diynes that contained a thiophene or cyclopropane group with regioselectivity (12 : 1, 10 : 1). After treating the diethyl-substituted diyne, the compound 44e was produced 98% yield with significant regioselectivity (17 : 1). With high regioselectivity (8 : 1) the target product 44f was produced with 98% yield from the terminal diyne. The final product of other substituted diyne was 44g with 98% yield. By applying this approach, various bioactive molecule substituted alkyne derivatives could be modified to produce the products (45 with 98% yield and 46 with 98%) in quantifiable amounts and with great regioselectivity (8 : 1, 10 : 1).^29^

Through the [2 + 2 + 2] cycloaddition of a nitrile and two alkynes, which is catalysed by a transition metal, multisubstituted pyridines^108–111^ can be produced, which are used to synthesize complex natural products^112,113^ and bioactive compounds that contain pyridines.^114^ Nitrile and internal alkynes were subjected to a cycloaddition reaction using the most earth-abundant and economically advantageous cobalt catalyst. In order to synthesize monocyclic, tetra- and pentaryl-pyridines with an incomparable degree of structural variation, the catalyst was simply developed using CoI_2_, dppp and Zn.

**Scheme 9:**
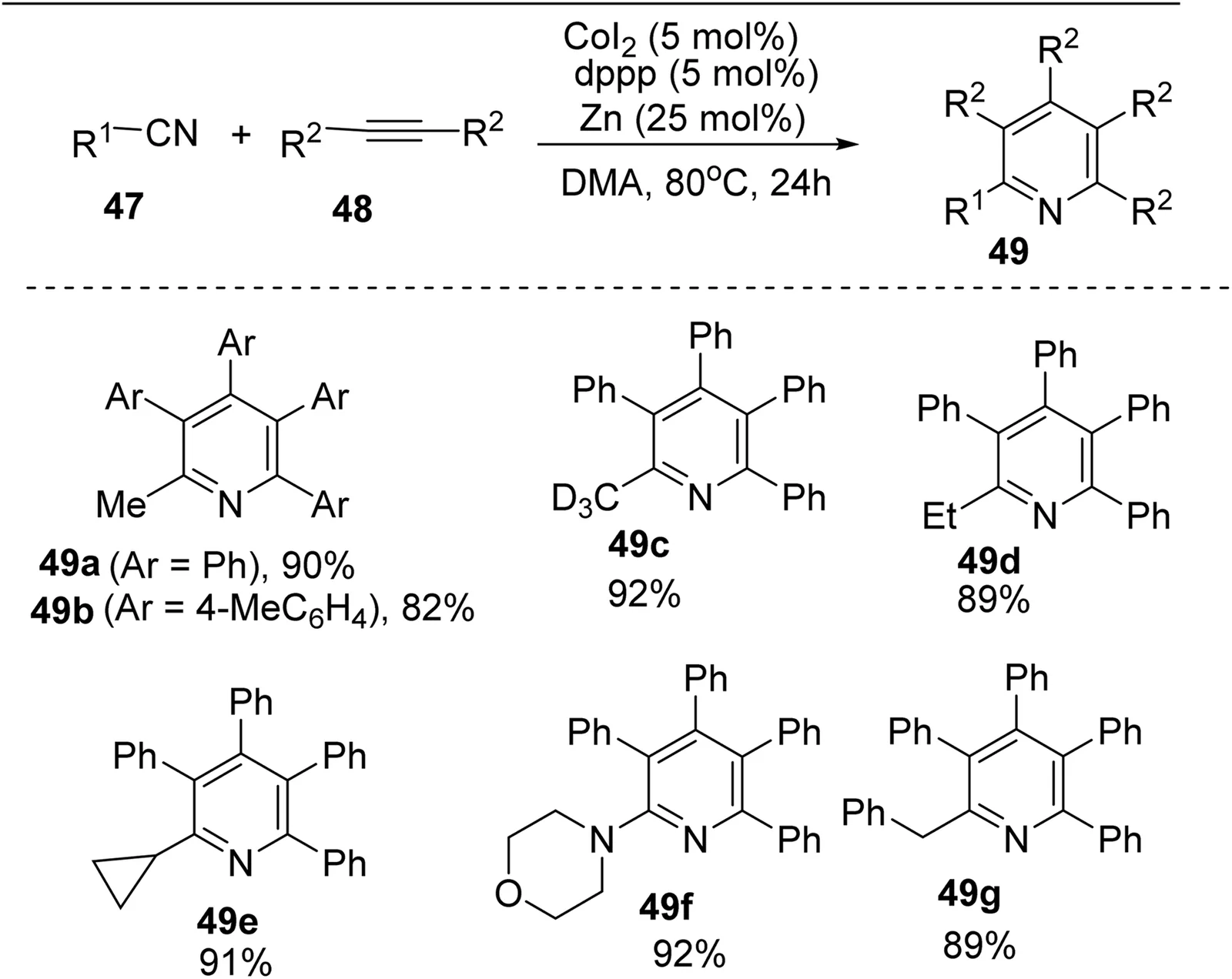
For the synthesis of poly-arylpyridines, DMA = dimethylacetamide.

Wang *et al.*, under optimum reaction conditions explored the range of substrates for alkyl nitriles 47 through [2 + 2 + 2] cycloaddition. It was discovered that an altered catalytic mechanism containing CoI_2_ (5 mol%), 1,3-bis(diphenylphosphino)propane (5 mol%) and Zn (25 mol%) in DMA effectively accelerated the reaction at the expense of a significant surplus of MeCN producing the desired product 49a in 90% yield. *Para*-substituted diphenylacetylenes and MeCN(methyl cyanide) might also be combined to produce 49b in good yields 82%. Product 49c was obtained in 92% yield when using CD_3_CN. Alkyl and benzyl nitriles that weren't sterically hindered, including those containing an olefin component took part in the reaction with 48 to produce the desired pyridine derivatives 49d 89% and 49e 91% in large quantities. In the literature, only the 2-alkyl-3,4,5,6-tetraarylpyridines 49a, 49d and 49g have been reported, this is indicative of the scarcity of relevant [2 + 2 + 2] cycloadditions. When combined with 48 a cyanamide derivative like morpholine-4carbonitrile also interacted to produce the 2-aminopyridine 49f in a high yield 92% (Scheme 9).^115^

**Scheme 10:**
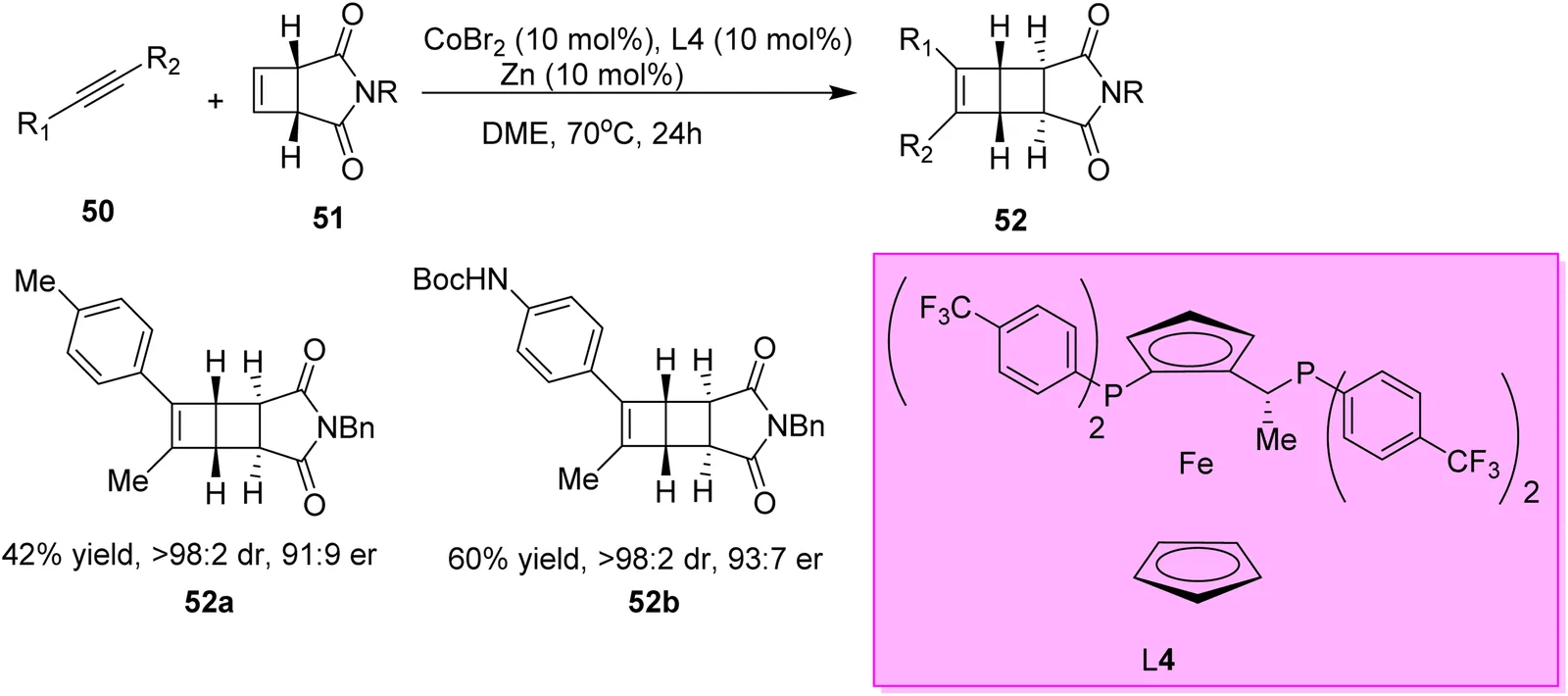
For cobalt-catalysed enantioselective [2 + 2] cycloaddition of alkyne and cyclobutenes.

**Fig. 9:**
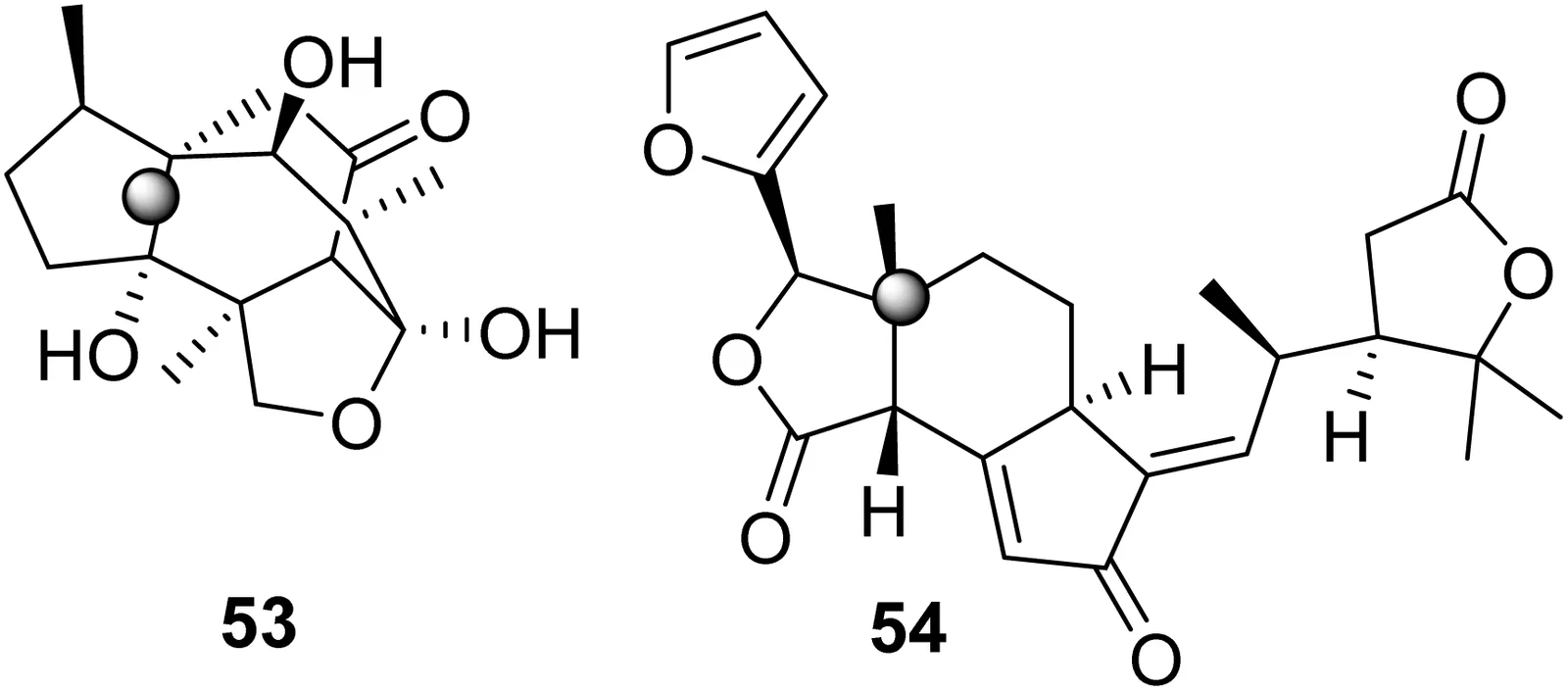
Biologically active natural compounds with all-carbon quaternary stereocenters and a 5-6-5 tricyclic structure.

A cobalt-catalysed procedure for divergent enantioselective coupling of alkynes and cyclobutenes is reported by Zhang *et al.*, with >98 : 2 regioselectivity having 42% yield 52a and diastereoselectivity and >99.5 : 0.5 enantiomeric ratio, with 60% yield 52b these processes, which start with oxidative cyclization and proceed with protonation or reductive elimination precisely regulated by ligands, yield densely functionalized cyclobutanes and cyclobutenes in up to 95% yield (Scheme 10).^116^ In order to synthesize the tricyclic cyclohexadiene's with quadratic bridgehead carbons present in a variety of naturally occurring biologically active compounds like 11-*O*-debenzoyltashironin 53 and perforanoid A 54 (ref. 117 and 118) [2 + 2 + 2] cycloaddition of enediynes by cobalt or photo redox catalysis has been employed (Fig. 9). Co/photo redox cooperative catalysis is used to achieve strong enantioselectivity in this reaction. The most enantioselective tricyclic framework with all carbon quaternary stereocenters may be generated *via* this technique which is the greatest way of producing biologically active molecules.

**Scheme 11:**
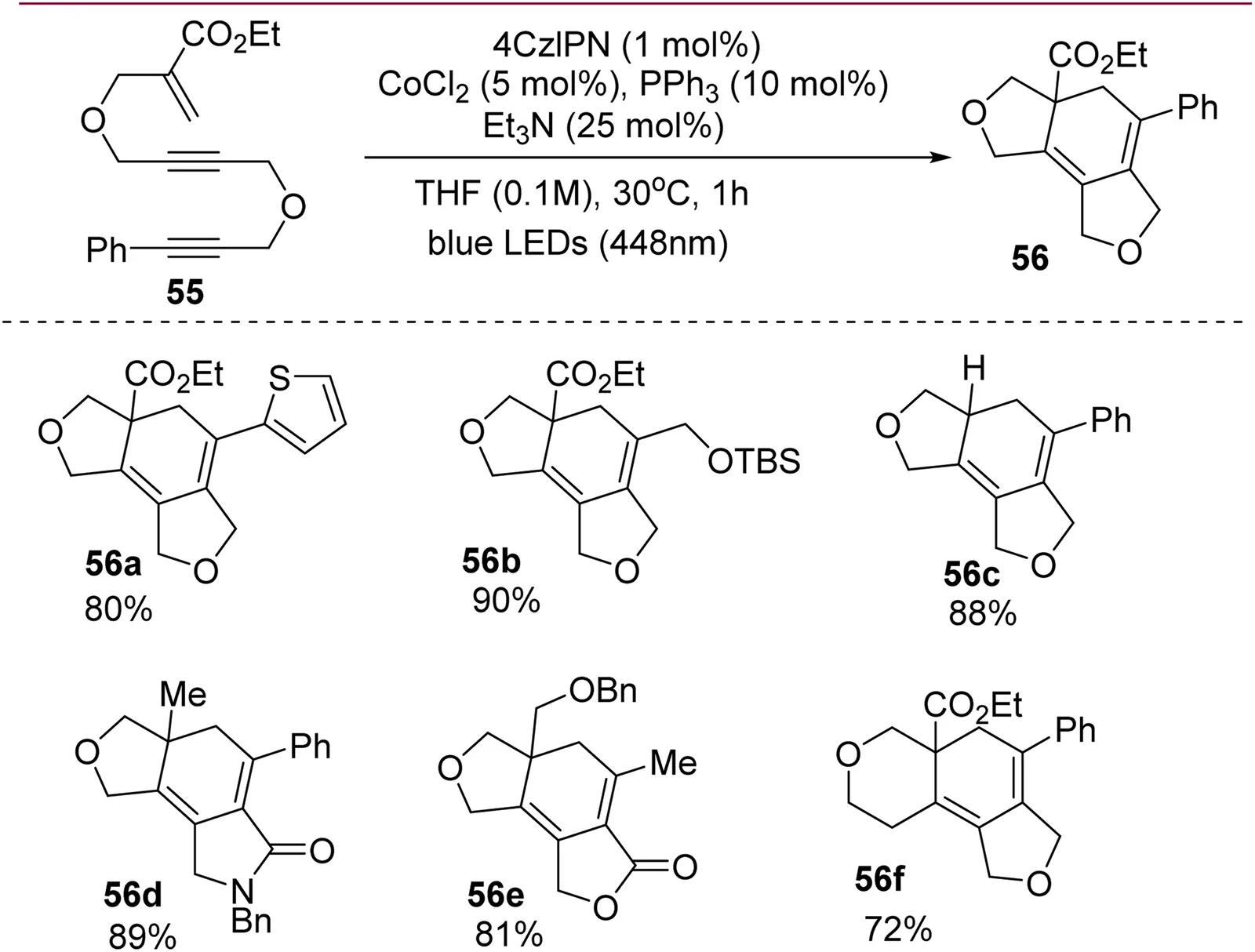
Enediynes' [2 + 2 + 2] cycloaddition for highly enantioselective conversion.

Under optimal reaction conditions Yasui *et al.*, investigated the [2 + 2 + 2] cycloaddition process of several enediynes. The related compounds 56a were produced with a high yield 80% from a substrate containing an alkyne terminal heteroaryl group. The substrate protected with *tert*-butyldimethylsilyl was effectively transformed into cycloadduct 56b 90% as a substitute strategy to introduce hydroxyl group. Endiyne underwent cycloaddition without incident and the product 56c was produced 88% in large quantities with no β-hydride elimination products. The amide group can be readily incorporated with regard to tether moieties and produces the appropriate 56d product in excellent yield 89%. The tricyclic lactone 56e was successfully produced 81% by successfully introducing the benzyloxymethyl group into the alkene moiety of ester-tethered enediynes. The 6-6-5 tricyclic cyclohexadiene 56f was successfully synthesized in good yield 72% with regard to the ring size of the cycloadduct (Scheme 11). Cobalt and photoredox catalysis enabled [2 + 2 + 2] cycloaddition of enediynes to produce tricyclic cyclohexadienes bearing a quaternary bridgehead carbon. The use of chiral ligand (*S*)-segphos, enabled a highly enantioselective reaction allowing access to highly enantio-enriched cyclohexadienes.^119^

### 2.4 Annulation reactions

Pharmaceuticals, natural products and biologically active compounds all contain nitrogen-containing heterocycles.^108,120–127^ He *et al.*, developed a novel technique for the synthesis of azaheterocycles. Cobalt catalyses an affordable hydroarylation and annulation reaction between maleimides and benzimidates in redox neutral conditions. This reaction forms the basis of the technique.

**Scheme 12:**
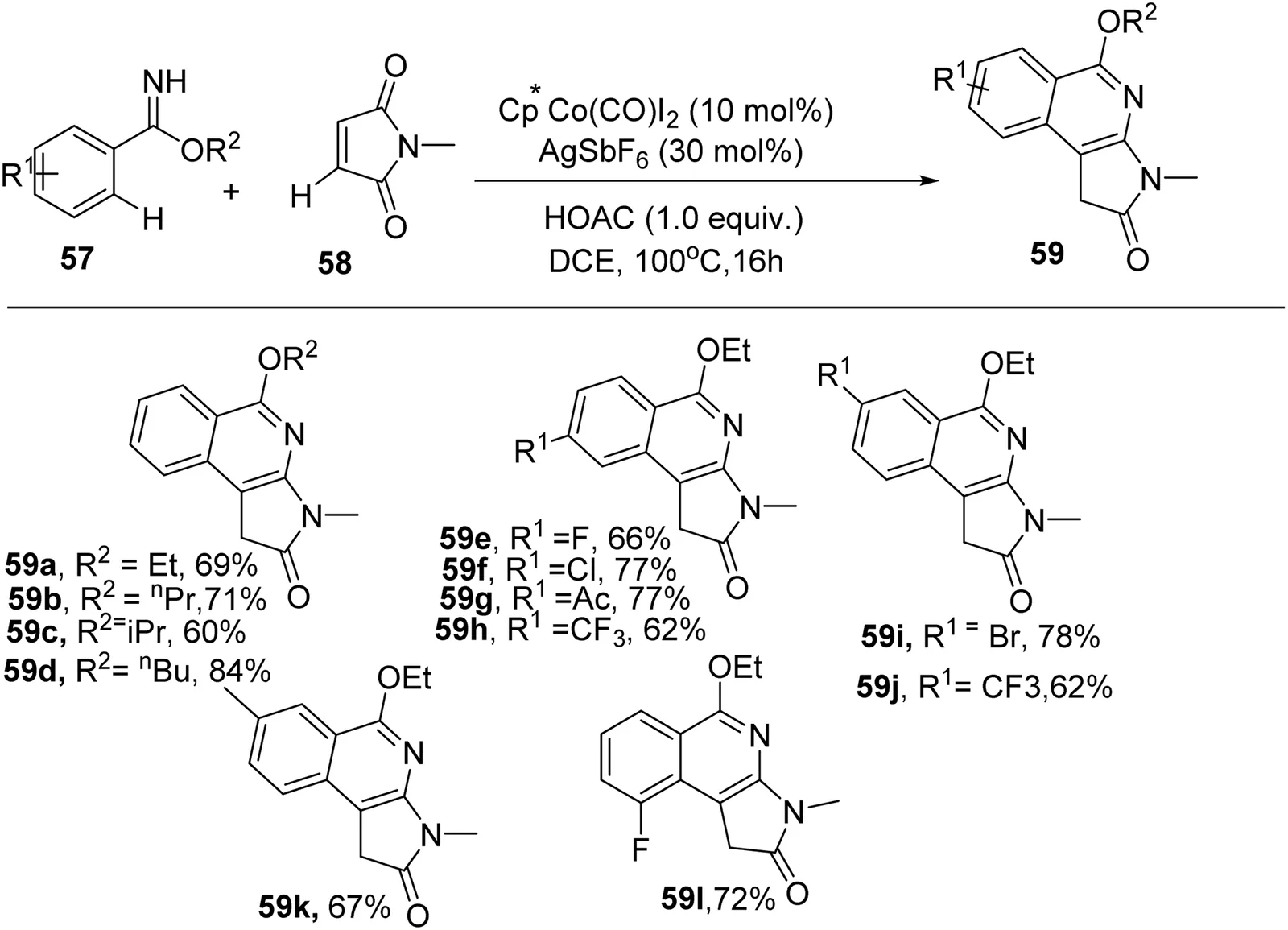
For the purpose to synthesize polycyclic compounds.

Firstly, He *et al.*, looked at the range of benzimidate substances in ideal conditions for the reaction. The desired polycyclic azaheterocycles 59a, 59b and 59d may be produced by this reaction using the benzimidate compounds made up of groups that donate and withdraw electrons. Other alkyl esters including the ethyl ester also smoothly reacted with the ethyl ester to produce good yields of the fused ring compounds 59c and 59d. In a reaction involving different functioning groups such as fluoro, chloro *etc.* and strong substituents that withdraw electrons such as acetyl and –CF_3_, targeted azaheterocycles 59e, 59f, 59g and 59h are produced in fair to good yields. With *meta*-substituted benzimidates a specific regioselectivity can be observed and only one isomer is produced (59k, 59i and 59j). The corresponding product 59l was generated by *meta*-fluoro substituted benzimidate in 72% yield. This results from the fluorine atom's tiny size and particular electronic properties (Scheme 12).

**Fig. 10:**
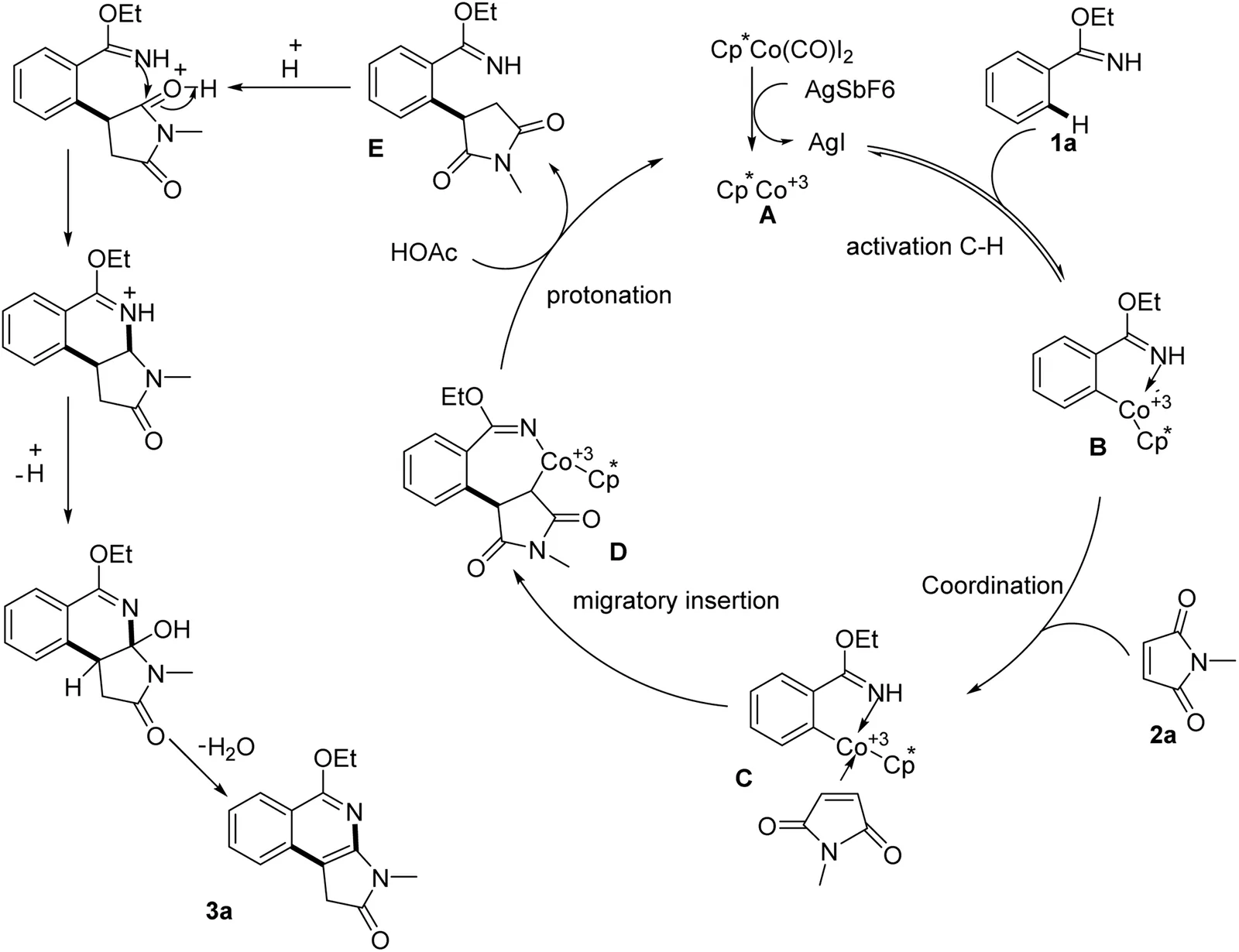
Proposed reaction mechanism for the synthesis of polycyclic azaheterocycles.

Fig. 10 shows a possible reaction mechanism. Initially, the AgSbF_6_ compound and cobalt catalyst combined to form the pre-activated catalyst Co^+3^ species A. The electrophile aromatic substitution process of benzimidate with species A then resulted in the production of the significant five-membered cobalt acyclic transition B, which coupled with maleimide to form product C. This process was reversible and involved a C–H activation. Complex C underwent migratory insertion, yielding intermediate D. The regeneration of the Co^+3^ species A that had been activated and intermediate E were the results of protecting species D. The final polycyclic nitrogen-containing heterocycle has been produced by annulation sequences on intermediate E in the presence of acid.^34^

**Fig. 11:**
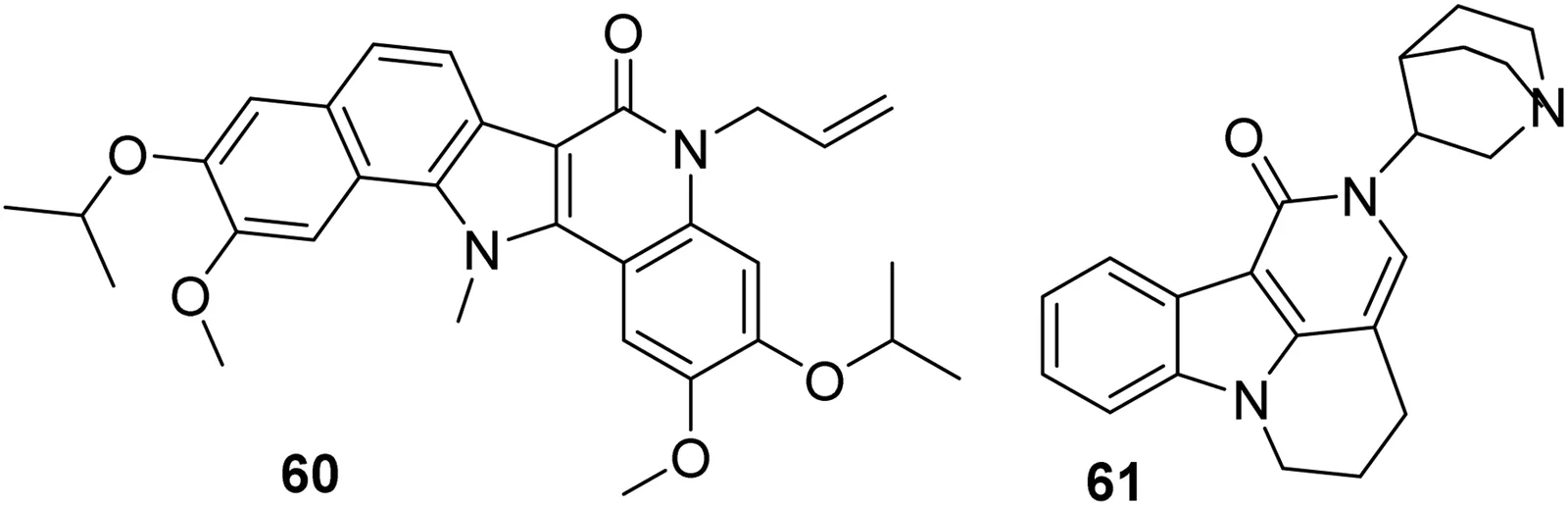
Biologically active indololactams.

Complexes of multicyclic indole are abundantly found in numerous naturally occurring and biologically active compounds.^128^ Due to their beneficial pharmacological properties, such as checkpoint kinase 13 with inhibitory properties 60 (ref. 129) as well as strong affinity for the 5-HT3 receptor 61,^130^ indololactams have been recognized as a significant family of polycyclic indole drugs (Fig. 11). Therefore, organic chemists are quite interested in the synthesis of indololactams. Researchers have developed structurally complicated six-membered indololactam derivatives by using affordable cobalt salt with supporting ligand by using salicylaldehyde to C–H/N–H annulate indoloamides with alkynes.

**Scheme 13:**
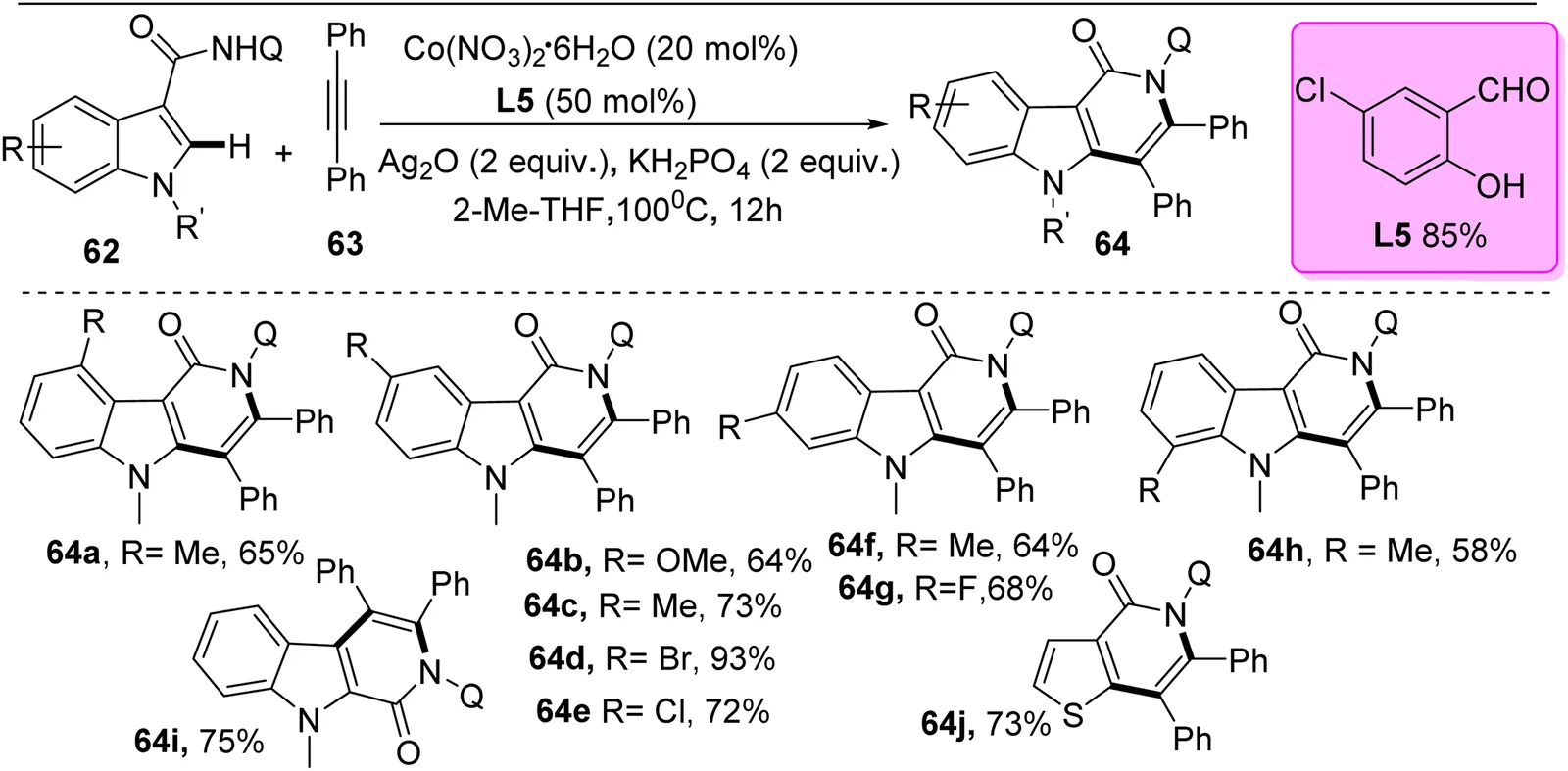
For producing an analogue of a 5-HT3 receptor antagonist, 2-Me-THF = 2-methyltetrahydrofuran.

Huang *et al.*, examined the scope of various indole substrates with alkyne 63 under optimal reaction conditions (Scheme 13). The annulation process was smoothly carried out by indole having amides at 3 position with various functional groups at positions 4 to 7 such as methyl to produce the appropriate products 64a, 64c, 64f and 64h with low yields. Regarding indole's phenyl ring several substituents such the MeO, F, Cl and Br groups were all converted to their respective products 64b, 64g, 64e and 64d, respectively. Under some circumstances, indole-2-amide can react producing the desired product 64i with a 75% yield. Other heteroaromatic substrates such as thiophene-3-carboxylic amide also yielded the same product 64j with a yield of 73%. The reaction is unaffected by monodentate coordination groups such as benzylamine.^131^

**Scheme 14:**
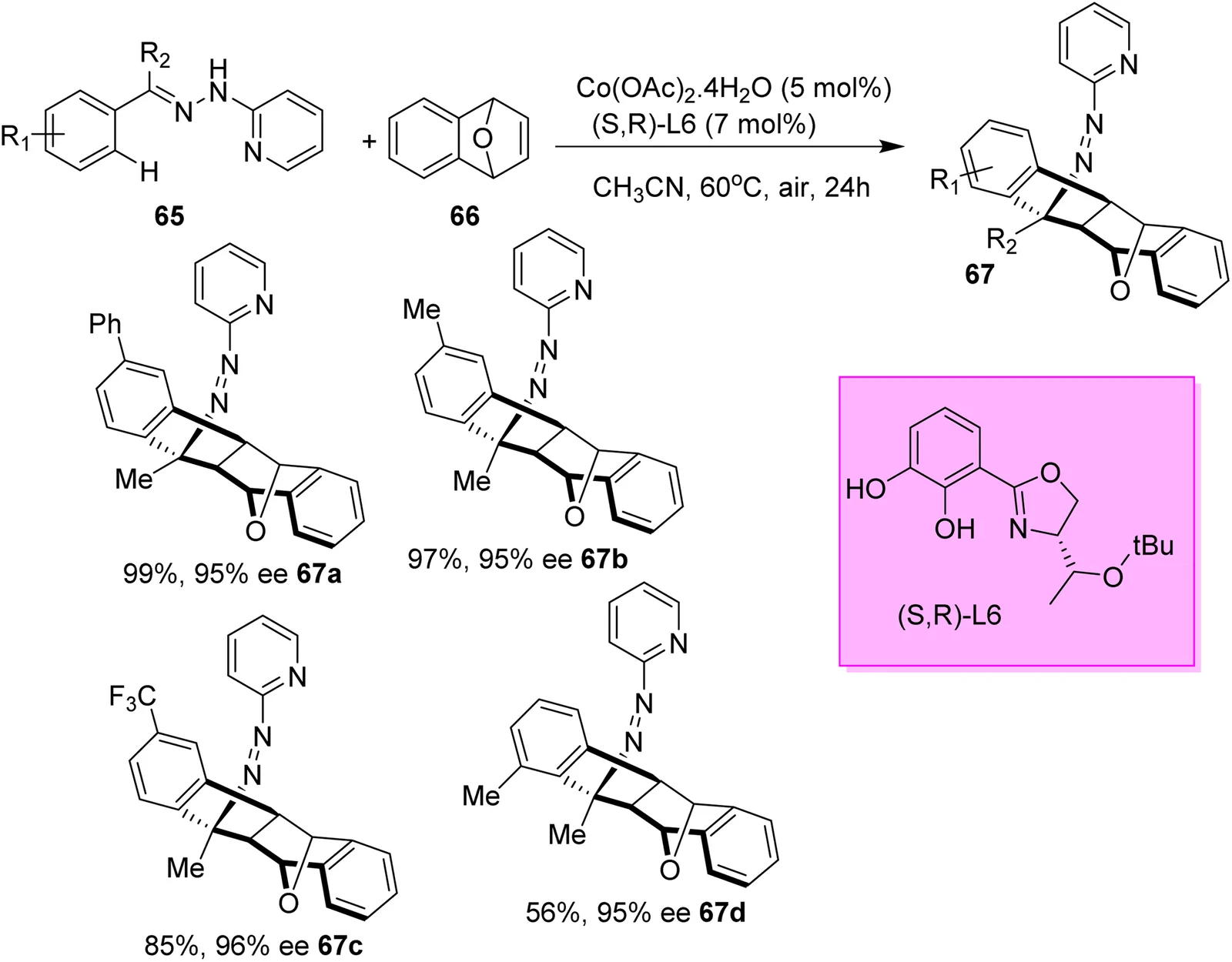
Enantioselective transformation of aryl hydrazones *via* cobalt catalyst.

Using symmetrical bicyclic alkenes 65, aryl hydrazone 66 and an unprecedented cobalt-catalysed enantioselective C–H activation/nucleophilic [3 + 2] annulation, Huang *et al.*, create the domino transformations. The techniques provide easy access to a large number of chiral compounds with four or five consecutive stereocenters in a single step that have [2.2.1]-bridged bicyclic cores. Excellent enantioselectivity results from the orientation of bicyclic alkenes being directed by the well-defined chiral pockets created by asymmetric coordination around the trivalent cobalt catalyst (Scheme 14).^132^

**Fig. 12:**
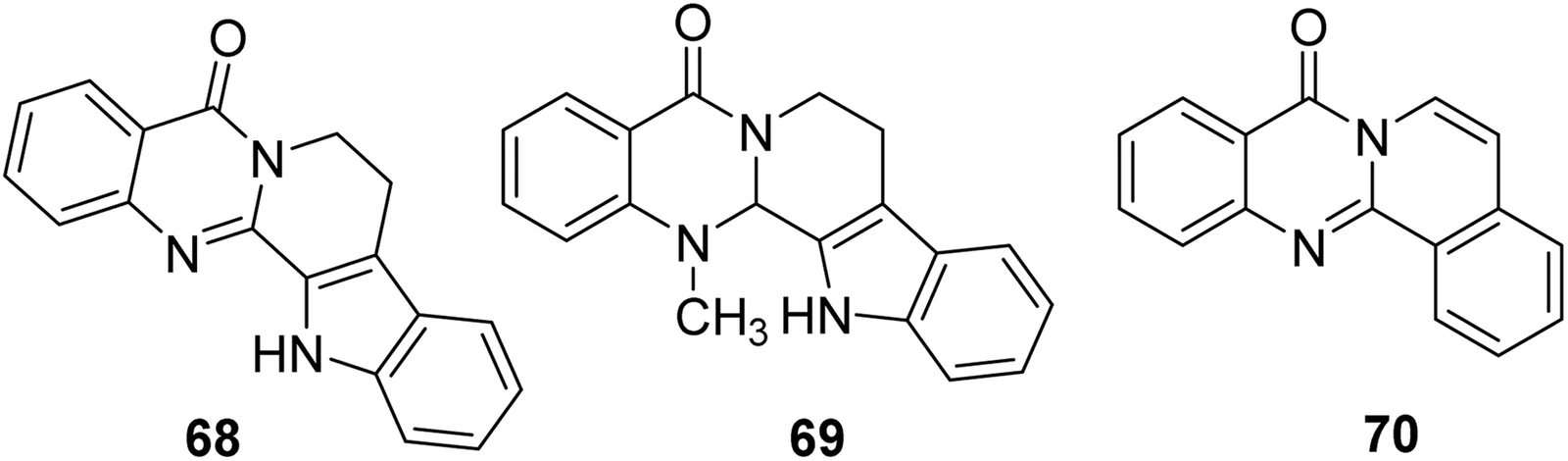
Specific quinazolinone scaffolds that are bioactive.

The substituted quinazolinones which are significant heterocyclic compounds, are of great interest to synthetic and biological chemists.^133–137^ They can be found as organic compounds, medicinally active compounds and many other natural products.^138–142^ Quinazolinone derivatives 68, 69, 70 have been synthesized using a variety of techniques, however they all require for tedious reaction processes and challenging reaction conditions (Fig. 12). As a result, 2-arylquinazolinones were cyclized with alkynes using a moderate Co^+3^-catalysed reaction in order to produce highly substituted quinazolinones .

**Scheme 15:**
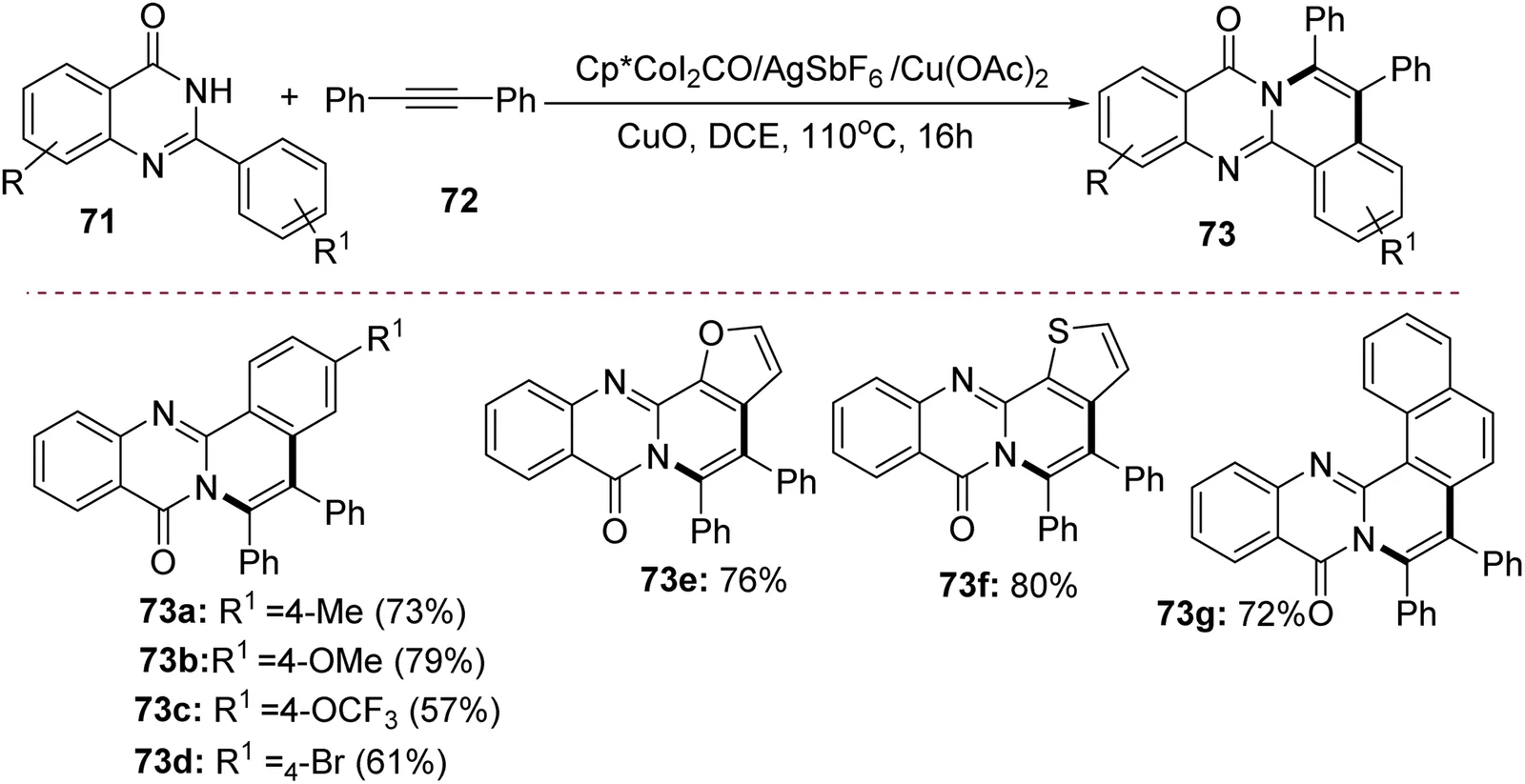
For the synthesis of fused quinazolinones.

To determine the annulation reaction's extent Kumaran *et al.*, studied the interactions between various 2-arylquinazolinones and alkynes under optimal reaction circumstances. The quinazolinones that had 4-methyl and 4-methoxy substituents on the 2-phenyl ring efficiently reacted with alkyne 72 to produce the desired products (Scheme 15) 73a and 73b. When 4-OCF_3_ and 4-Br on a 2-phenyl ring are treated with alkyne 72, the corresponding products 73c and 73d are produced. These results unequivocally demonstrate that substituents with electron-donating groups exhibited more reactivity as compared to those groups which withdraw electrons. Investigations on the effects of converting aryl groups to heteroaryl groups have additionally been carried out. Following treatment of 2-furyl- and 2-thienyl-quinazolinones with alkyne 72, the corresponding compounds 73e and 73f were obtained in 76% and 80% yields, respectively. The bulkier 1-naphthyl quinazolinones provide a 73g yield in 72% of the equivalent product.^143^

**Fig. 13:**
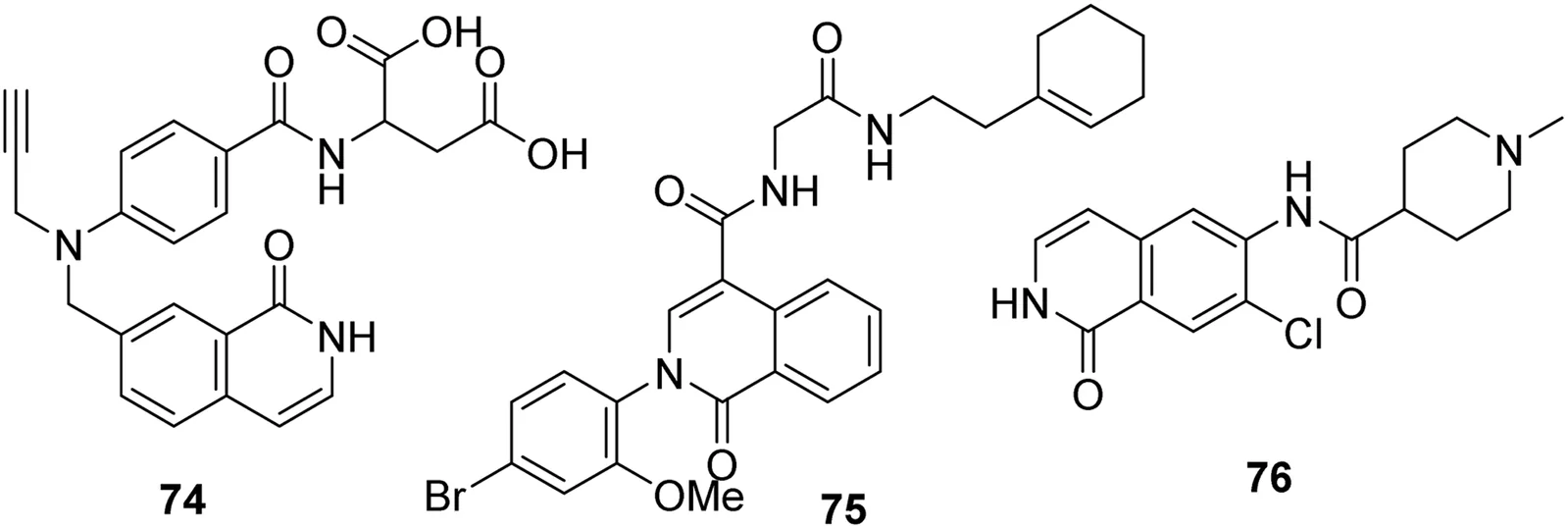
Numerous substances with the potential to be bioactive.

The class of favoured skeletons known as iso-quinolones is observed in a variety of medicines, organic compounds and biologically active compounds (Fig. 13).^144–147^ For instance, it was discovered that compound 74 had anti-tumour action and was a thymidylate synthase inhibitor.^146^ Fluconazole's antifungal efficacy against *Candida albicans* is significantly enhanced by compound 75. A possible anti-hypertensive agent, compound 76 has been identified.^147^ An effective and simple technique has been reported for the synthesis of such significant structural components by researchers which involves the annulation of amides with alkynes under the influence of transition metals.

**Scheme 16:**
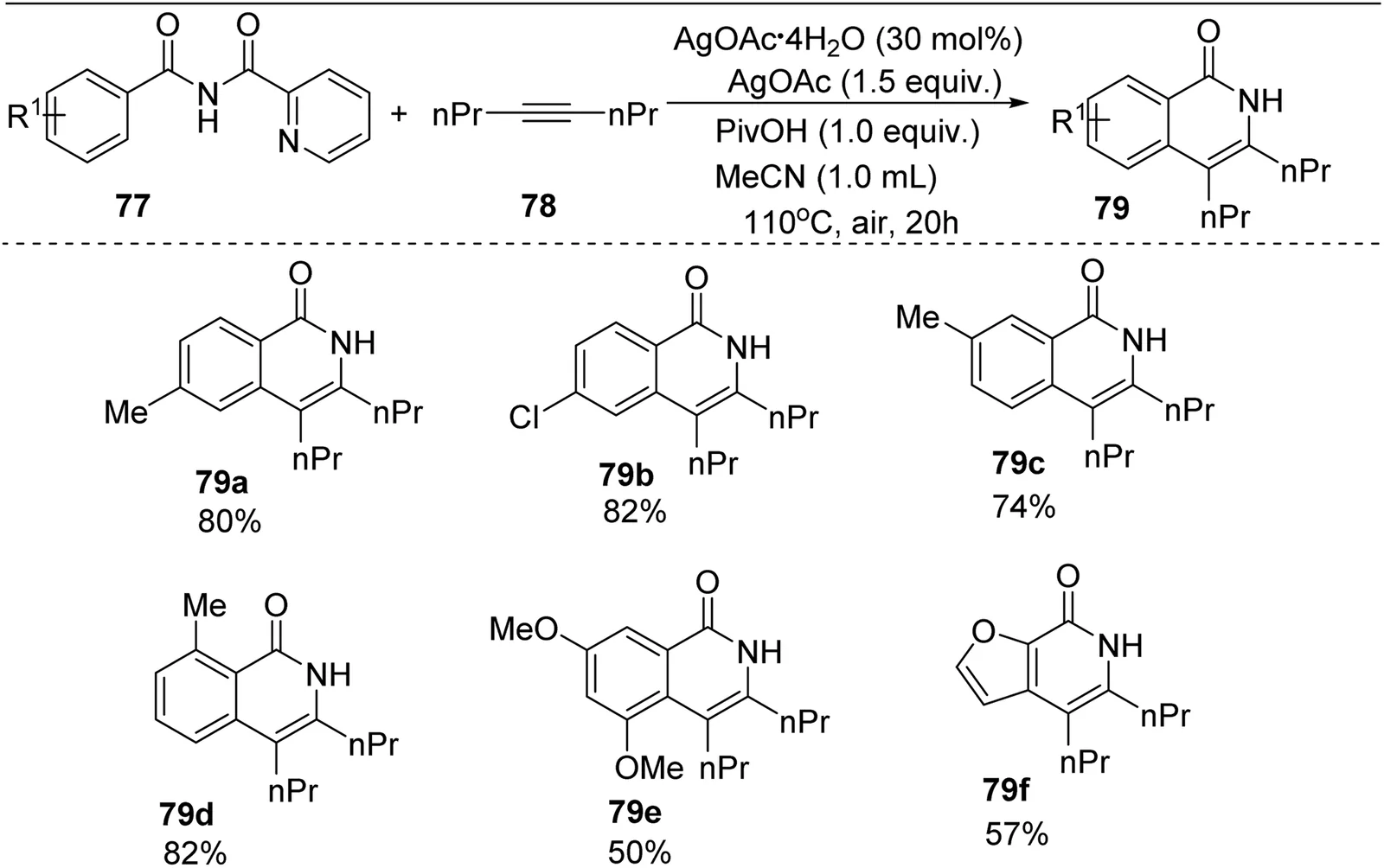
For the synthesis of (NH)-isoquinolones with alkynes.

Wang *et al.*, researched the range of *N*-aroylpicolinamides 77 in the reaction with 4-octyne 78 under optimal reaction conditions (Scheme 16). The reaction produced corresponding products 79a and 79b in good yields for *N*-aroylpicolinamides bearing either p-electron-donating or p-withdrawing substituents. It was demonstrated that when *ortho*- or *meta*-substituted compounds were reacted, large yields of the products 79c and 79d were produced. Additionally, the di-substituted *N*-aroylpicolinamide reaction went successfully and produced product 79e. A furan unit-containing substrate was effectively transformed to product 79f with a yield of 57%.

**Fig. 14:**
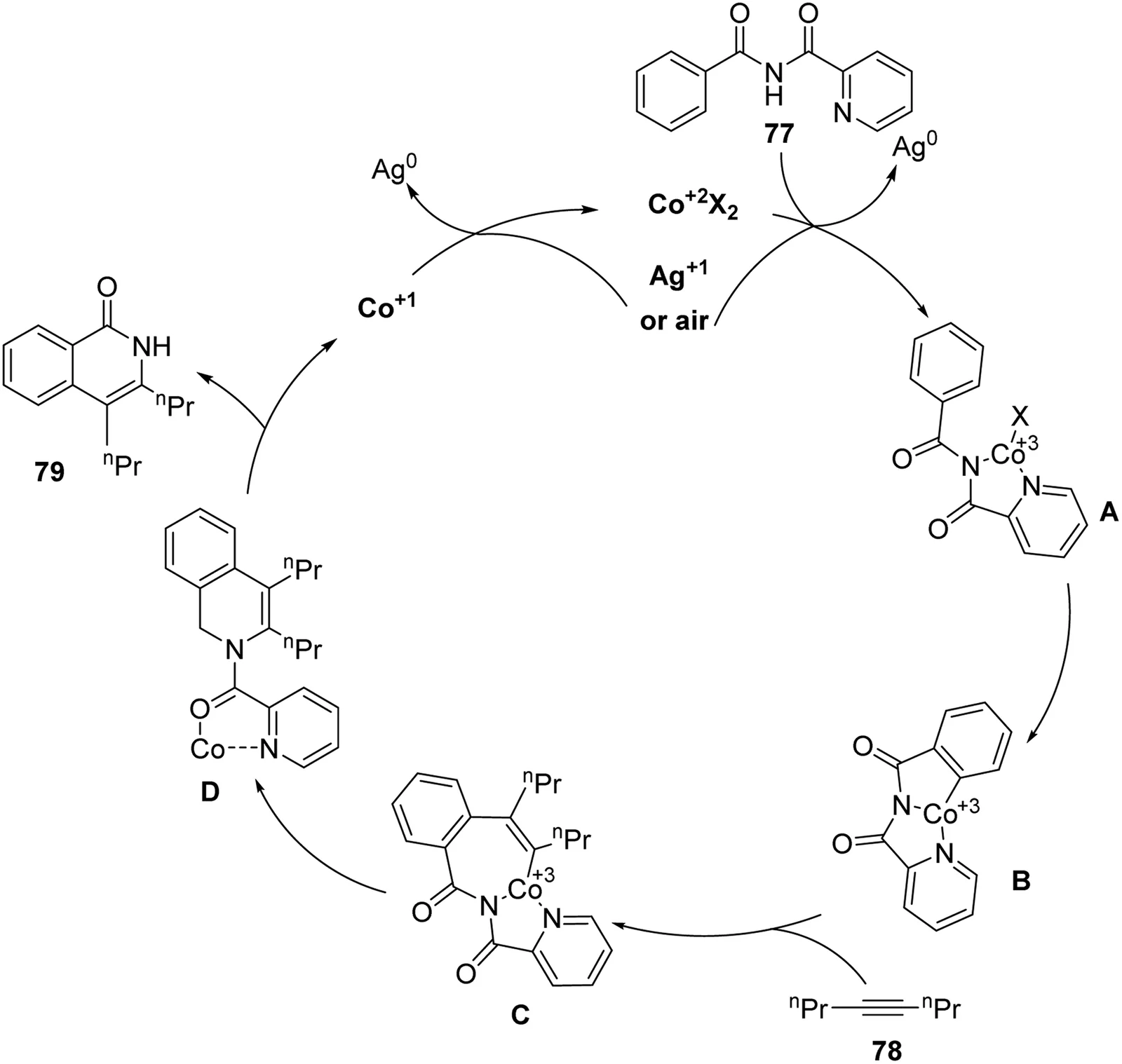
A possible reaction mechanism.

For a cobalt-catalysed C–H annulation of *N*-aroylpicolinamides with alkynes, a feasible mechanism is put forth (Fig. 14). The Co^+3^ species is initially produced by the Ag(i) salt oxidizing the Co^+2^ pre-catalyst and it combines with 77 to form the Co^+3^ complex A. The Co^+3^ intermediate B is then produced by activating the arene C–H bond in A. The Co^+3^ complex C is produced by the coordination of the alkyne 78 with the cobalt metal and subsequent incorporation into B. The Co^+1^ complex D is formed after the reductive elimination of C and it is hydrolysed in order to generate the target product 79 and liberate the Co^+1^ species. It is possible to obtain 2-picolinic acid that can act as the directing group. The active Co^+2^ catalyst is then restored for the subsequent catalytic cycle by oxidizing the Co^+1^ species with air or Ag^+1^ salt.^148^

Quinolines are a group of highly important *N*-heterocycles that are frequently used in biologically active compounds (such as anti-HIV, anti-malarial and anti-inflammation agents)^149,150^ and marketing drugs.^151^ By cyclizing 2-aminoaryl carbonyls and alkynoates, which are often exploited for the development of bioactive and therapeutical compounds, ester modified quinolines can be formed.^152^ Using a new earth-abundant N-doped ZrO_2_@C hold up cobalt nanocatalyst exhibiting consistent dispersion and strong catalytic activity in the reductive annulation of 2-nitroaryl carbonyls with alkynoates and alkynones a variety of quinolines with broad substrate scope have been synthesized.

**Scheme 17:**
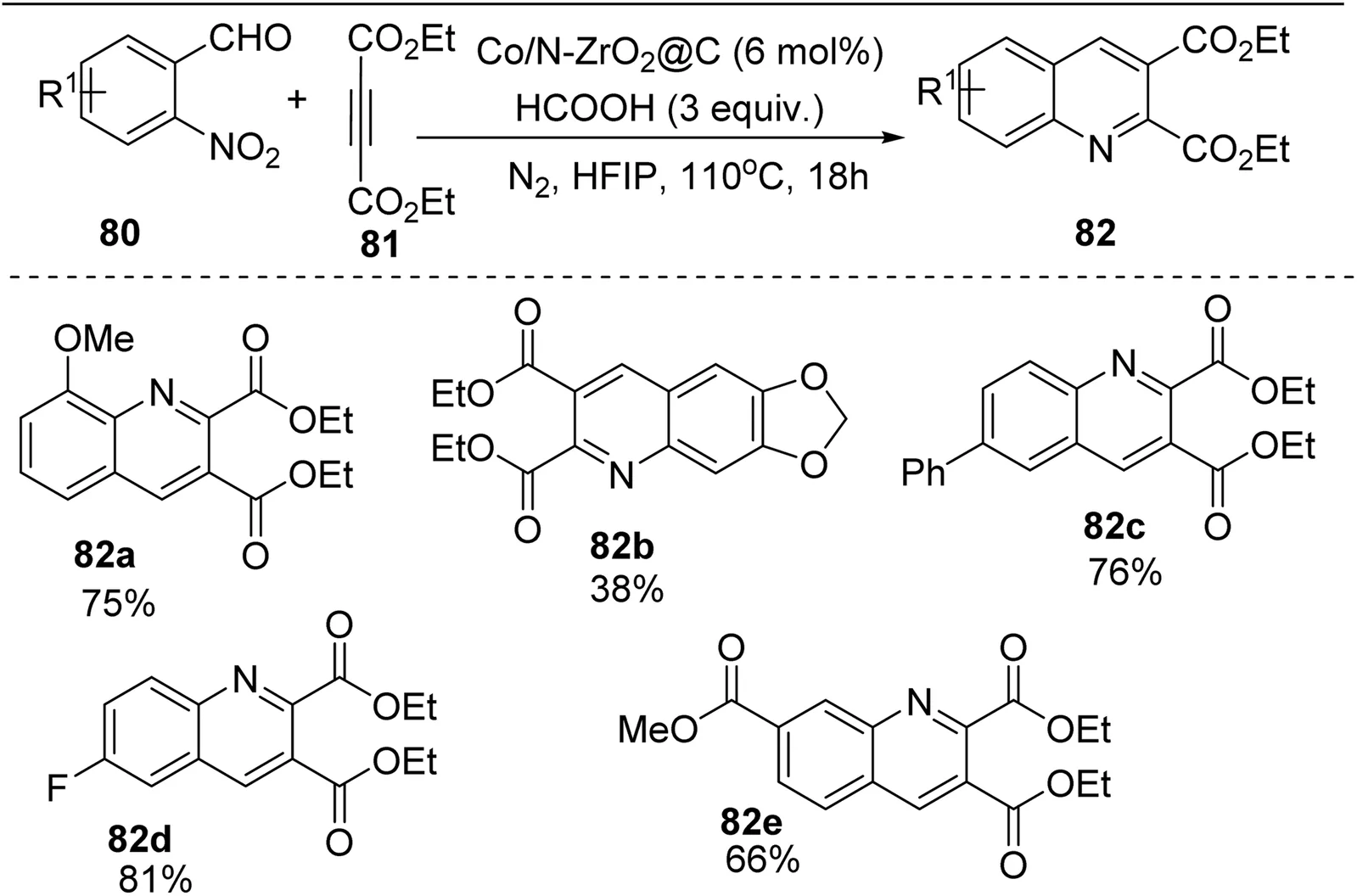
For the synthesis of functionalized quinolines.

Xie *et al.*, performed analysis of the substrate scope in Scheme 17 with regard to the optimum reaction conditions. Alkynoate 81 was combined with a number of 2-nitrobenzaldehydes 80, which were all tested. The necessary quinoline products were produced in rather excellent yields 82a, 82b and 82d as illustrated in Scheme 16 and the process overall underwent smooth hydrogenative annulation. Under optimal reaction circumstances a variety of functional groups including –Me, –OMe, –NMe_2_, –OH, –Ph, –F, –Cl, –Br, –COOMe, and –NO_2_ on substrate 80 were well tolerated. The electronic properties of these functionalities had a slight impact on the product yields. Particularly, reactants 80 bearing electron-withdrawing groups produced the desired product 82d and 82e in relative yields that are greater than those of substrates containing strong electron-donating substituents 82b. This is likely because the electron-rich 2-nitrobenzaldehydes are less beneficial to reduction of the nitro group, whereas the electron-withdrawing groups could enhance the reactivity of the carbonyl group in substrate 80, thus favouring the condensation step (Scheme 17).^153^

**Scheme 18:**
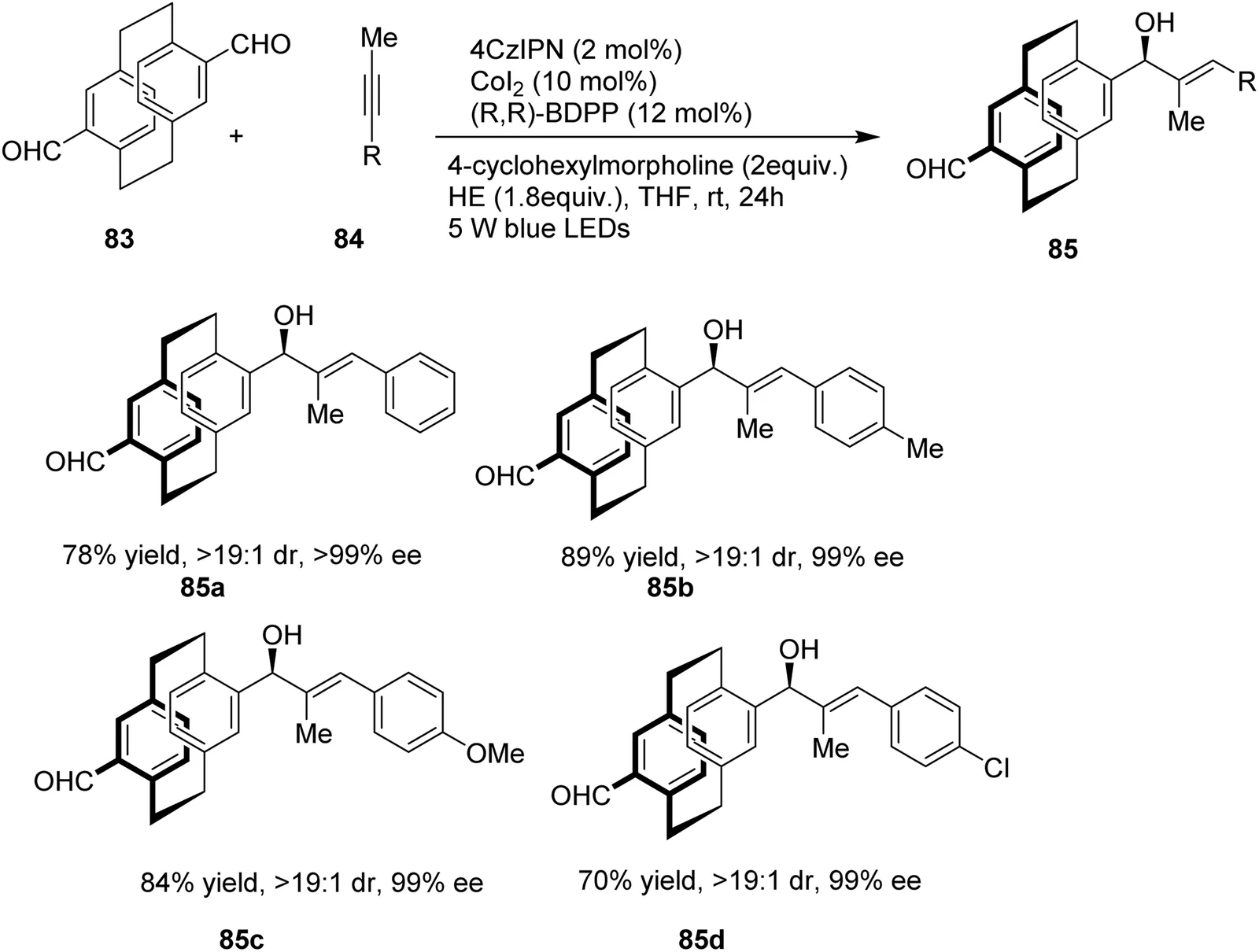
Synthesis of planar PCPs *via* de-symmetrization.

**Fig. 15:**
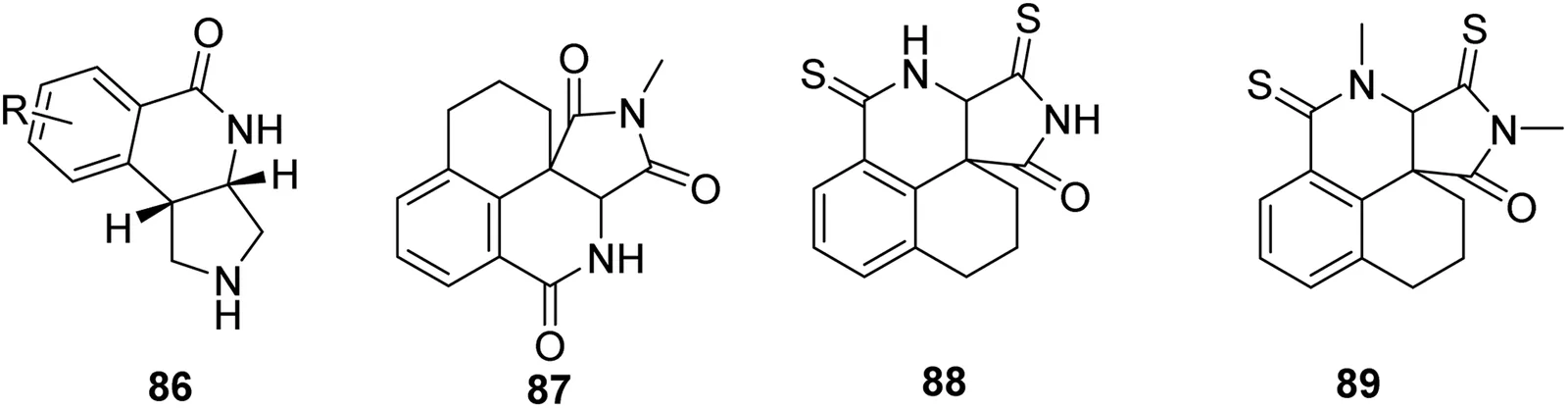
Pyrroloisoquinoline trione derivatives that are bioactive.

A photoinduced Co-catalysed asymmetric addition reaction of 83 with alkynes 84 was created by Liu *et al.*, when combined with chiral alcohols, the desymmetrization approach produced a variety of planar chiral [2,2]paracyclophanes (PCPs) (85) in good yields with outstanding enantio- and diastereocontrol. This procedure allowed for the simultaneous production of planar and central chirality using asymmetric metalloredox catalysis (Scheme 18).^154^ Maleimide derivatives are found in numerous naturally occurring compounds and molecules that are biologically and pharmaceutically active.^155–158^ These play a part in C–H activation processes and the synthesis of the relevant succinimide compounds. It is simple to transform the succinimide moieties into pyrrolidines that are relevant to biological processes, gamma-lactams and potentially useful derivatives.^159–162^ In order to produce 3-arylated succinimides, the first successful conjugate addition of maleimides *via* a C–H activation approach has been documented.^163–168^ Maleimides are also well known for 1,1-type and 1,2-type cyclization reactions^29,168,169^ as well as the base-mediated Heck-type reaction, in addition to conjugate addition reactions.^301^Fig. 15 contains a pyrroloisoquinoline trione derivatives 86, 87, 88, 89 that are biologically active.

**Scheme 19:**
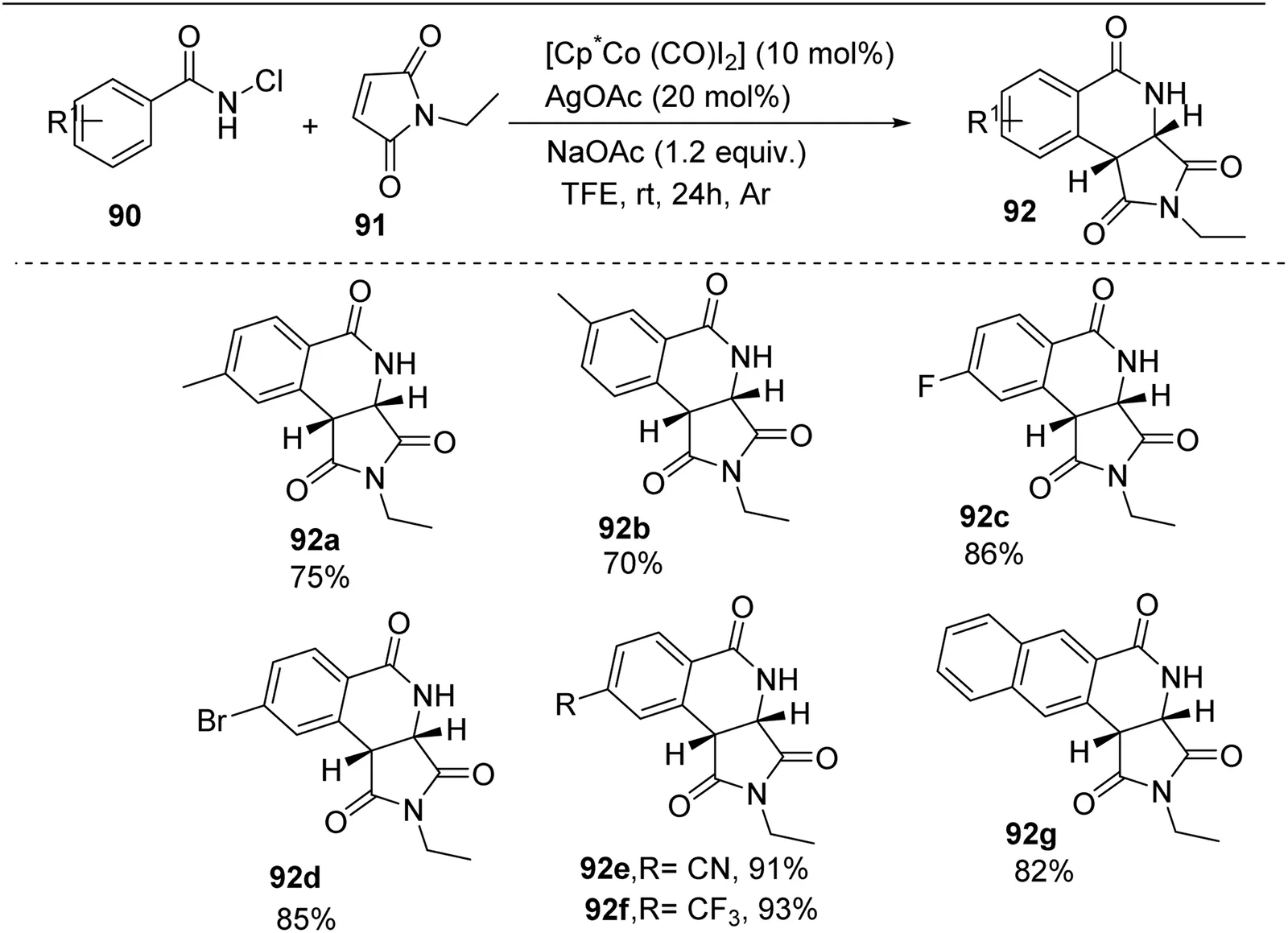
For *N*-chlorobenzamide annulation with maleimides, TFE = tetrafluoroethylene.

Muniraj *et al.*, (Scheme 19) has examined the scope of the reaction with *N*-chlorobenzamide derivatives 90 under optimal reaction conditions. When *N*-chlorobenzamides containing 4- and 3-Me substitutes were combined with *N*-ethylmaleimide, the corresponding annulated products 92a and 92b were produced in 75 and 70% yields, respectively. *N*-Chlorobenzamide exhibited reactivity in the *ortho*-position, which was sterically free. Halogenated *N*-chlorobenzamides had high reactivity with *N*-ethylmaleimide resulting in yields of 86 and 85% for the corresponding annulated products 92c and 92d, respectively. Electron-withdrawing groups like cyanide and trifluoromethyl at the *para* position of *N*-chlorobenzamide derivatives underwent a smooth reaction to obtain the corresponding products 92e and 92f with 91 and 93% yields, respectively. The corresponding annulated product 92g was similarly produced by *N*-chloroamide generated from naphthyl in an 82% yield.

**Fig. 16:**
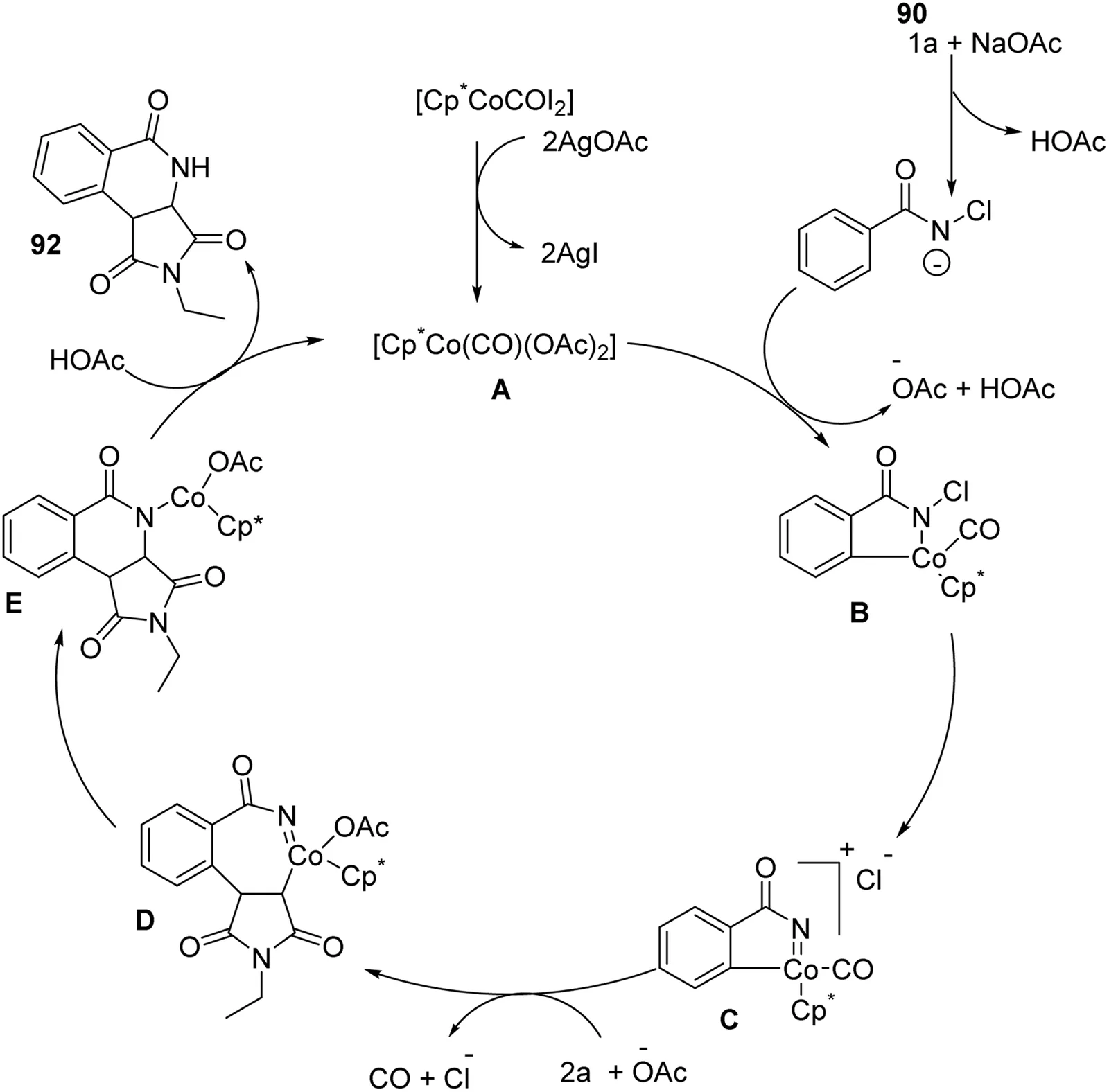
For proposed reaction mechanism.

In Fig. 16, a plausible mechanism has been put forth in accordance with the control studies and prior research. The *in situ* produced catalytically active specie A combines with the anionic form of 90 to form the cobalt acycle B, which is then oxidized to make the intermediate C by converting Co^+3^ to Co^+5^. A reductive elimination results in the formation of the Co^+3^ intermediate E after the insertion of maleimide generates the 7-membered intermediate D. The desired product 92 is produced as a result of the subsequent proto-de-metalation of species E, which also results in the regeneration of the Co^+3^ catalyst.^170^

### 2.5 Carbonylation reaction

Ethers can be found in a wide variety of medicines, agrochemicals and bioactive products.^94,171,172^ Ethers are present in many drug compounds including ether-containing medicinal. As allergy medications and antibiotics, fenofibrate and cetirizine are used to treat urticarial infections, allergic rhinitis, dermatitis and abnormal amount of lipids in blood. Ethers can be employed as carbonylation substrates which serve as important constituents in the immediate alkoxycarbonylation reaction catalysed by cobalt. The corresponding alpha-oxy esters derivatives were produced in high yield from a variety of alcohols and phenols, numerous medications and bioactive compounds are also appropriate substrates for this incorporation of carbon monoxide to the organic molecules.

**Scheme 20:**
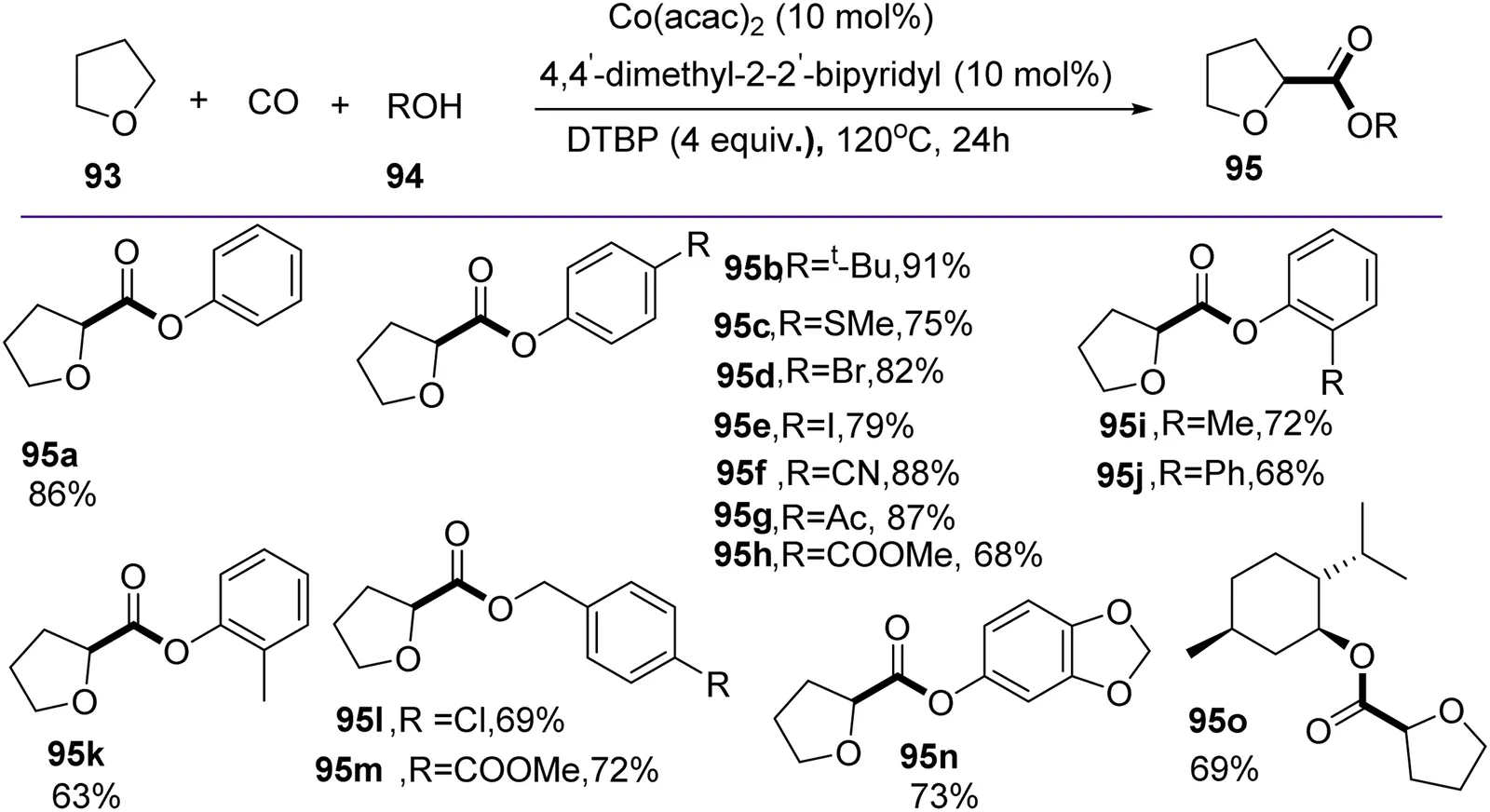
For the generation of phenolic and alcoholic α-oxy esters, DTBP = di-tertiary butyl peroxide.

Without the use of a catalyst, phenol and tetrahydrofuran (THF) react under CO pressure to produce the desired product in low yields. The target product 3′ formed 71% when Co(acac)_2_ served as the catalyst and the supportive ligand 6,6′-diMe-bpy was present. The most favourable outcomes were achieved with 4,4′-dimethyl-2,2′-bipyridine used as the ligand and Co(acac)_2_ as the catalyst. The yield of 3′ considerably increased when the amount of DTBP was increased. The carbonylation product was increased to 83% by raising the temperature while using less catalyst and ligand. Wang *et al.*, tested a variety of ethers and phenols/alcohols under optimal reaction conditions. Several phenols with groups that donate or withdraw electrons were studied in the first step. For the intended carbonylation products, yields ranging from 39 to 91% were obtained (Scheme 20). Although these substrates showed similar yields 95a–95k and the effects of the electronic environment on the reaction are minimal.

The high compatibility of the reaction was confirmed by the ability to maintain the active halogen atoms at various positions on the aromatic 95d, 95e and 96k. The target product can be produced with good yields using other substituents like –CN 95f, –Ac 95g, and –COOMe 95h. As phenol's 95i, 95j steric hindrance increased, the yield gradually decreased. Many substituted alcohols, especially benzyl alcohols 95l–95m, have yields of aliphatic esters that have been thoroughly studied. In this reaction, two naturally occurring compounds, sesamol and dl-methanol reacted favourably in terms of yields 95n–95o.^173^

**Fig. 17:**
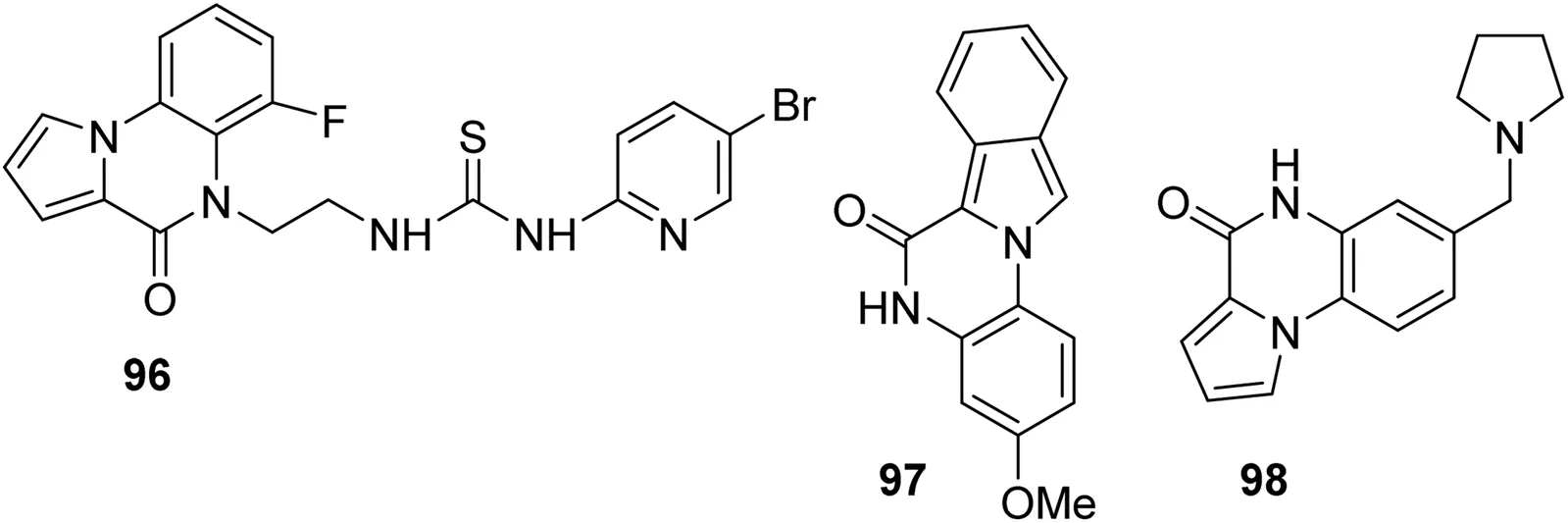
Biologically active compounds showing different activities.

A variety of medicines and bioactive substances have pyrrole[1,2-α]quinoxalin-4(4*H*)-ones as abundant scaffolds.^174–176^ Compound 96, for instance in Fig. 17 serves as an example of an anti-HIV drug by blocking non-nucleoside reverse transcriptase.^174^ Compound 97 is a potent inhibitor of the polymerization of tubulin and topoisomerase I that has a high cytotoxic effect on a range of human cell types.^175^ Compound 98 an inhibitor of poly(ADP-ribose) polymerase-1 (PARP-1) with potent enzymatic and cellular efficacy is extensively utilized in the treatment of cancer.^176^ The pyrrole[1,2-α]quinoxalin-4(5*H*)-ones due to their wide range of biological effects have attracted the interest of many scientists.^177^ For the purpose of efficiently synthesize these molecules, researchers therefore adopted this innovative approach employing cobalt-catalysis.

**Scheme 21:**
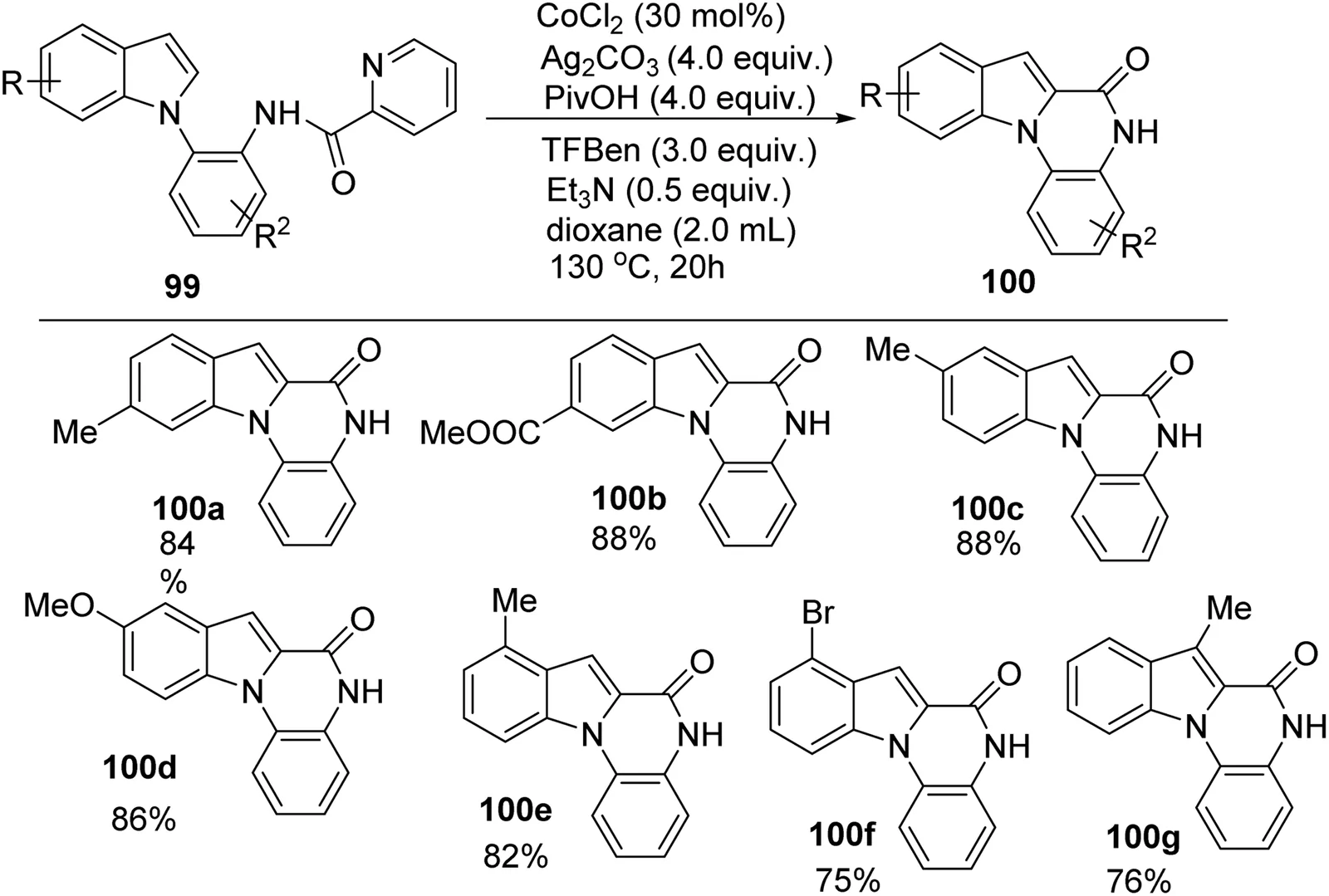
For producing free (NH)-indole[1,2-α]quinoxalin-6(5*H*)-ones, TFBen = benzene-1,3,5-triyl triformate.

Gao *et al.*, studied a number of *N*-(2-(1*H*-indol-1-yl)phenyl)picolinamides 99 under optimum reaction conditions, as illustrated in the Scheme 21. When compounds are present at position C6 of the indole ring, such as –Me and –COOMe, the reaction results in the corresponding products 100a and 100b. When C5 functional group-containing substrates were subjected to a reaction, the corresponding products 100c and 100d were generated with high yields. The smooth reaction of the C4-substituted compounds produced the product 100e and 100f in high yields. The corresponding product 100g of the C3-Me-substituted compounds were successfully synthesized with a 76% yield. However, the reaction did not produce expected product when the indole ring's C3 position was replaced with a phenyl ring.

**Fig. 18:**
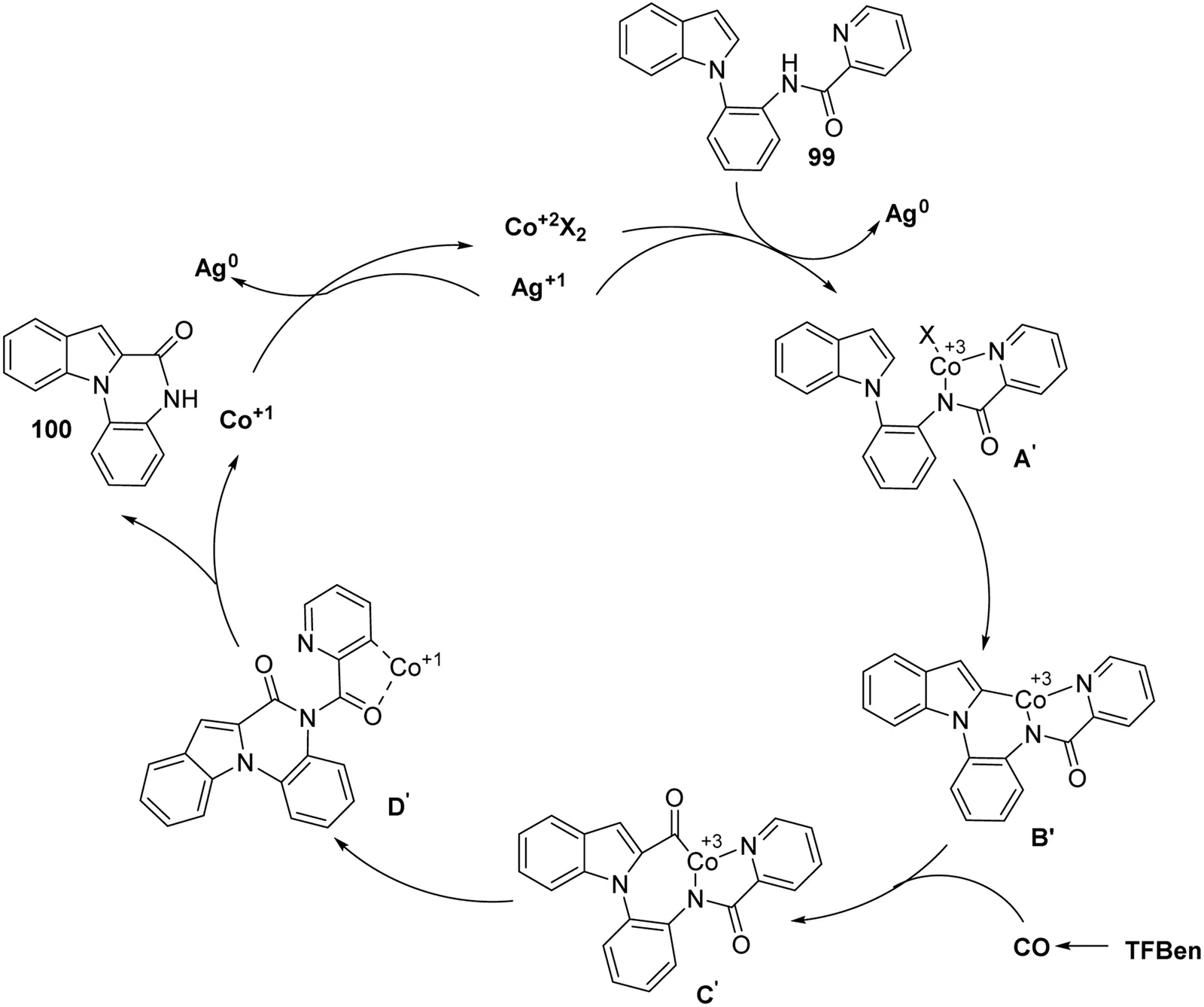
A plausible reaction mechanism.

Based on earlier studies, Fig. 18 proposes a tenable mechanism. The Co^+3^ species A′ is first formed by coordinating of *N*-(2-(1*H*-indol-1-yl)phenyl)-picolinamide 99 with the Co^+2^ catalyst and the Ag^+1^ salt's subsequent oxidation. The Co^+3^ complex B′ is then produced by activating the C–H bond at the C2 site in A′. The acyl Co^+3^ intermediate C′ is then produced by the insertion of carbon monoxide that is released from TFBen and by reductive elimination, it can be transformed into the Co^+1^ complex D′. The hydrolysis of D′ produces the desired product 100 and releases the Co^+1^ species as a result of the release of these species. By oxidizing the Co^+1^ species with the Ag^+1^ salt, the active Co^+2^ catalyst can be regenerated to enter the next catalytic cycle.^178^

**Fig. 19:**
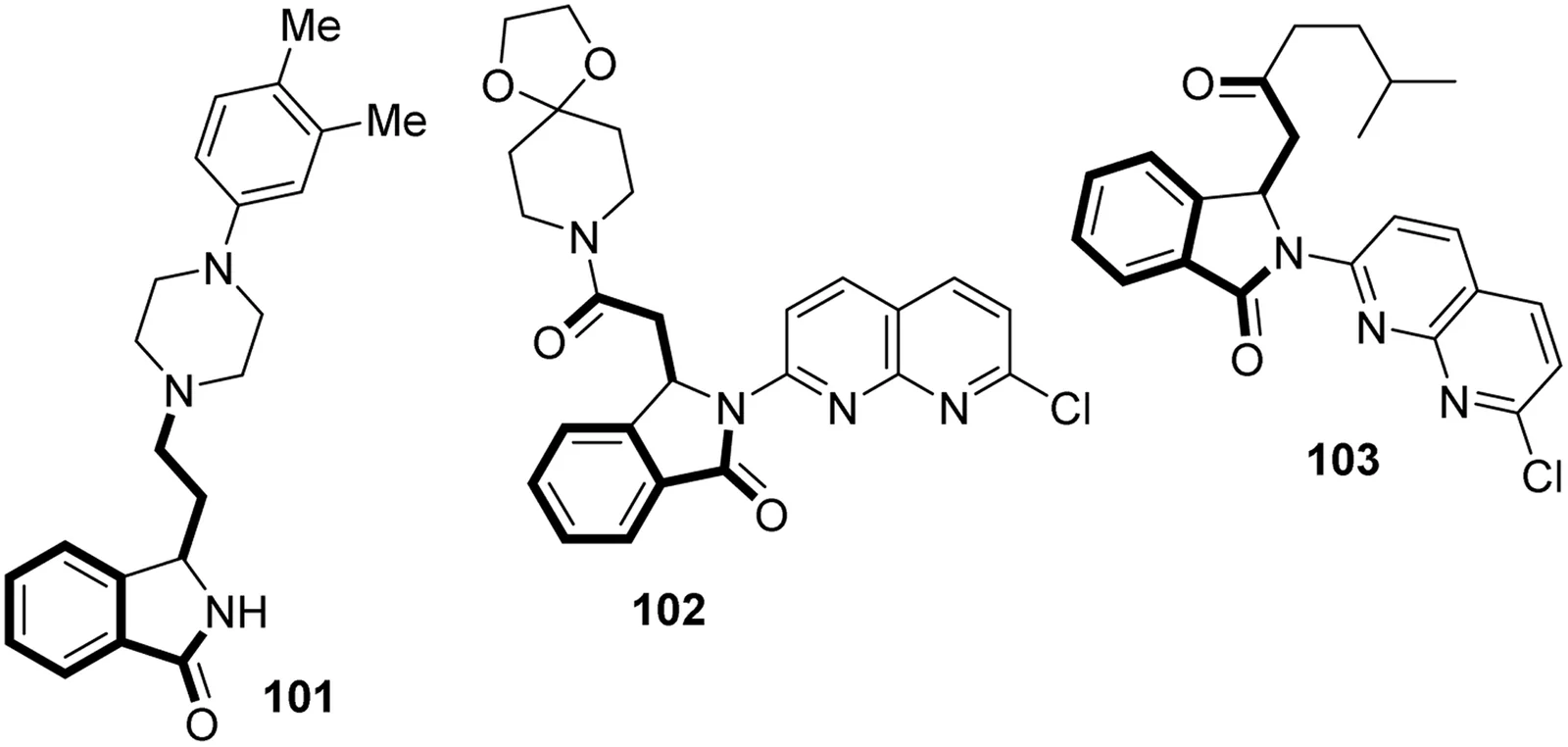
3-substituted isoindolinone compounds with biological activity.

The 3-substituted isoindolinones motif is a prevalent component in biologically active compounds such as alkaloids that are found in nature.^179,180^ There are many ways to make racemic isoindolinones having substituents as 3 position but only a handful of those ways can be used to make derivatives of 3-substituted isoindolinone that are enantiopure.^181,182^ The highly functional building blocks hydroxymethyl and 3-hydroxyethyl isoindolinones are utilized for synthesizing a variety of biologically active chemical compounds PD172939 101, pazinaclone 102 and pagoclone 103 (Fig. 19).^183–185^ Through C–H carbonylation of Phenylglycinol derivatives catalysed by cobalt, a highly effective technique for the production of 3-hydroxymethyl isoindolinones has been reported by researchers.

**Scheme 22:**
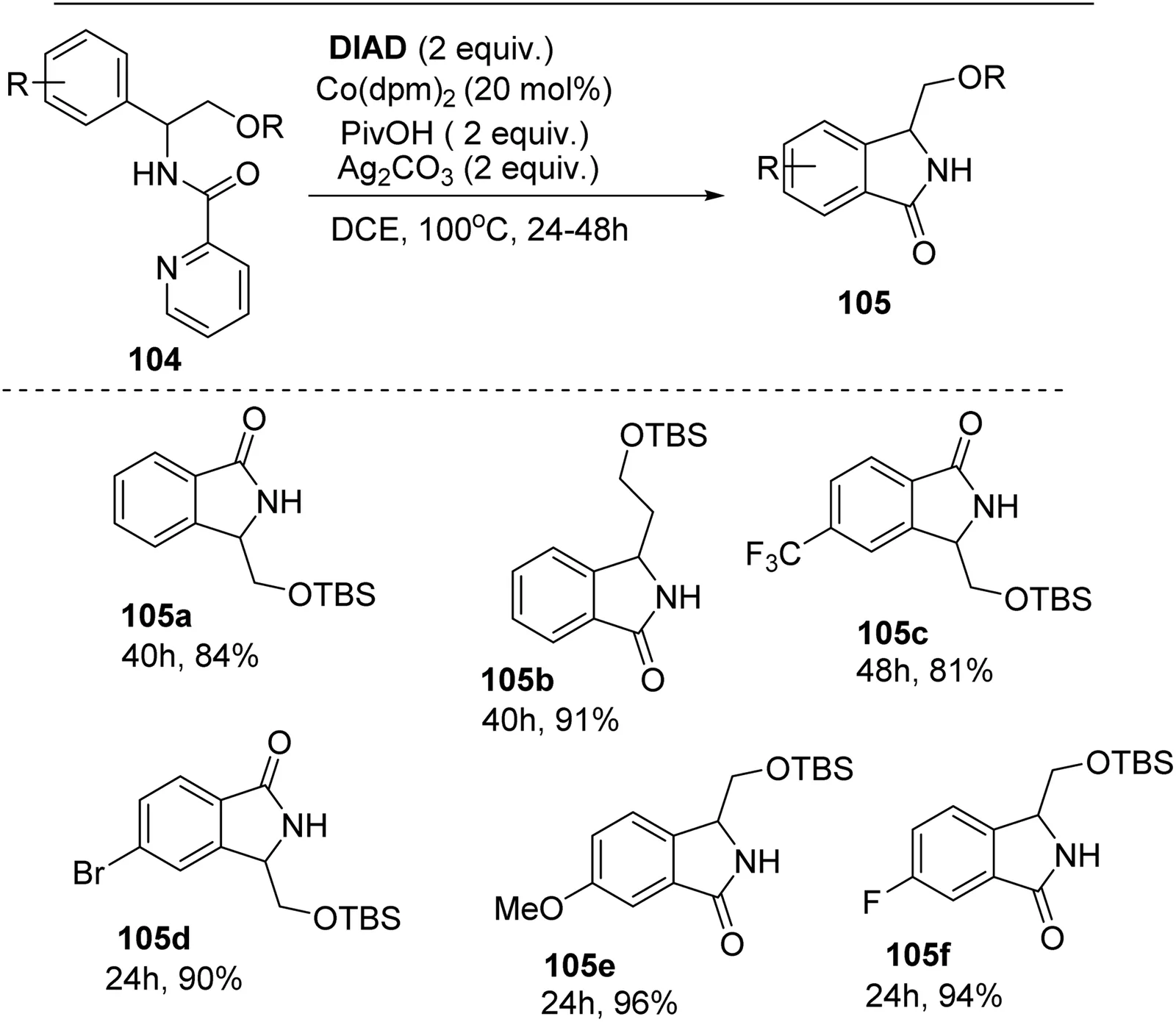
For the synthesis of 3-hydroxymethyl isoindolinones, DIAD = diisopropyl azodicarboxylate.

Lukasevics *et al.*, investigated the substrate scope under optimum reaction conditions. The equivalent isoindolinone product 105a was produced with a TBS protecting group in a very high yield (84%) The appropriate product 105b is produced in excellent yield (91%) by the reaction using the β-phenylalaninol derivative as a suitable substrate. Investigations have been performed on derivatives of phenylglycinol that have different functional groups on the benzene ring. The *meta*-substituted substrates give the equivalent product 105d from *ortho*-, *meta*- and *para*-substituted substrates with excellent regioselectivity, and the product is generated by a less hindered C–H bond's reaction. It is possible to allow a variety of functionalities including electron-donating groups like trifluoromethyl and electron-withdrawing groups like methoxy under reaction conditions to produce their corresponding products 105e and 105c, respectively. Halogenated phenylglycinol derivatives produced the required products 105d and 105f with an excellent yield (85–94%) (Scheme 22).^181^ Under cobalt-catalysis bioactive active substances are synthesized in high to excellent yield through carbonylation reactions. High functional group tolerance is exhibited by carbonylation processes carried out with cobalt-catalysis.

### 2.6 Hydro-arylation reactions

It is crucial to develop simple methods for the asymmetric production of diarylmethylamines because they are present in many biologically active compounds and medications.^186,187^ The enantioselective addition of aryl organometallic reagents to imines is now one of the most effective methods for producing optically active diarylmethylamines. This reaction is catalysed by transition metals. Researchers presented for the first time asymmetric reductive (hetero)arylation of imines utilizing aryl and heteroaryl halides, supported by a chiral cobalt-bisphosphine catalyst. With this approach, the use of organometallic reagents can be avoided and it works well with other functional groups.

**Scheme 23:**
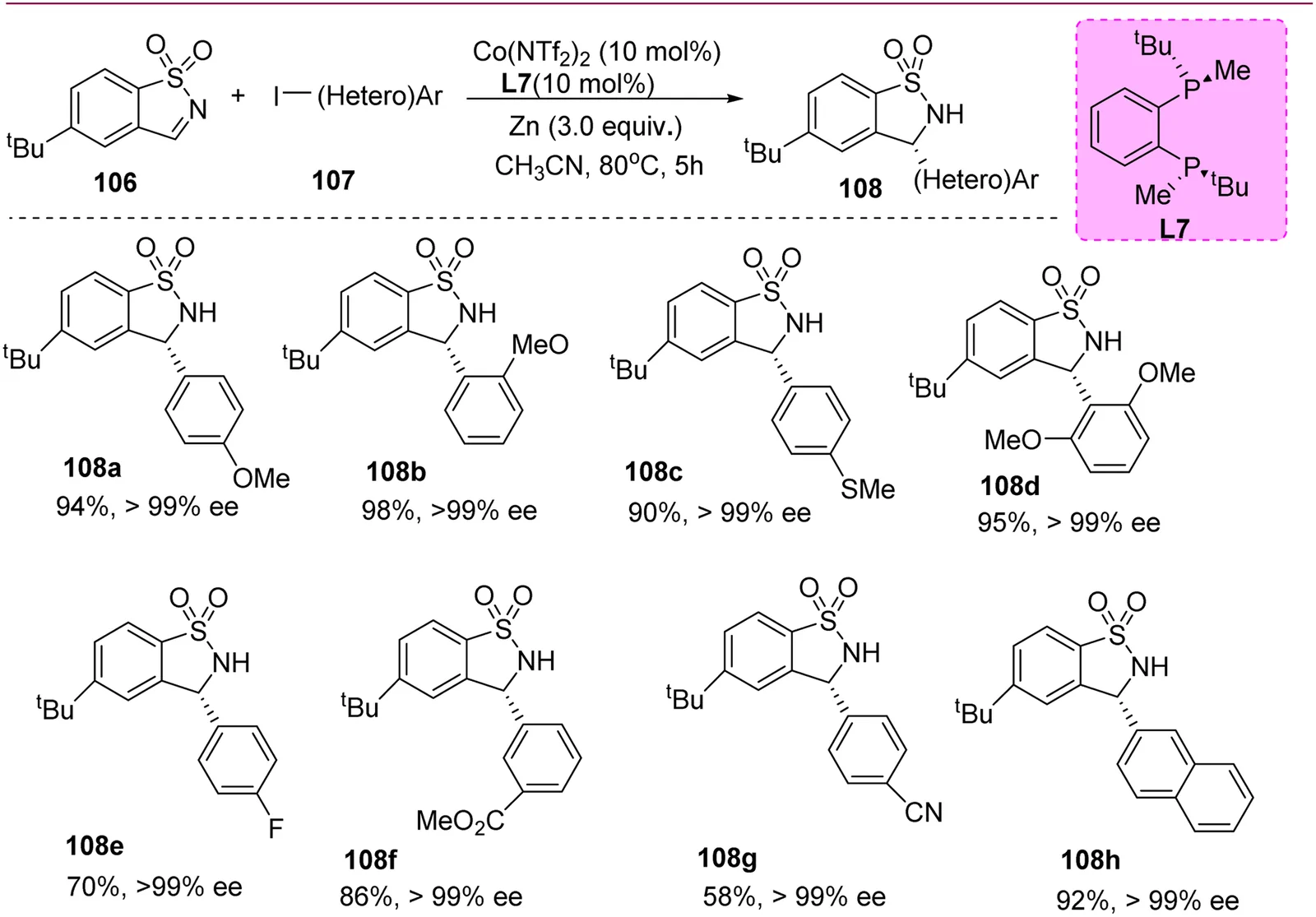
For compounds undergoing reductive arylation.

Xiao *et al.*, (Scheme 23) examined into the possibility for aryl iodides 107 to react with imines 106 after determining the optimal reaction conditions. Product 108a was produced in the presence of the ligand L7 and CoI_2_ as the precatalyst with 83% enantioselectivity but low conversion. Aryl iodide with electron-donating groups at the *ortho* and *para* positions, such as methoxy and methylthio groups, produces the desired product 108b and 108c in moderate to good yields with excellent enantioselectivities. The product 108d was produced from iodoarene that was hindered by two *o*-methoxy groups with a 90% yield and greater than 99% *ee*. Substrates containing halides easily react with imines to produce the desired product 108e. Additionally, iodoarenes with electron-withdrawing groups like ester and cyano substituents produce the compound 108f and 108g in yields varying from fair to excellent with strong enantioselectivities. The product 108h is produced with excellent efficiency by 2-iodonaphthalene.^188^

**Fig. 20:**
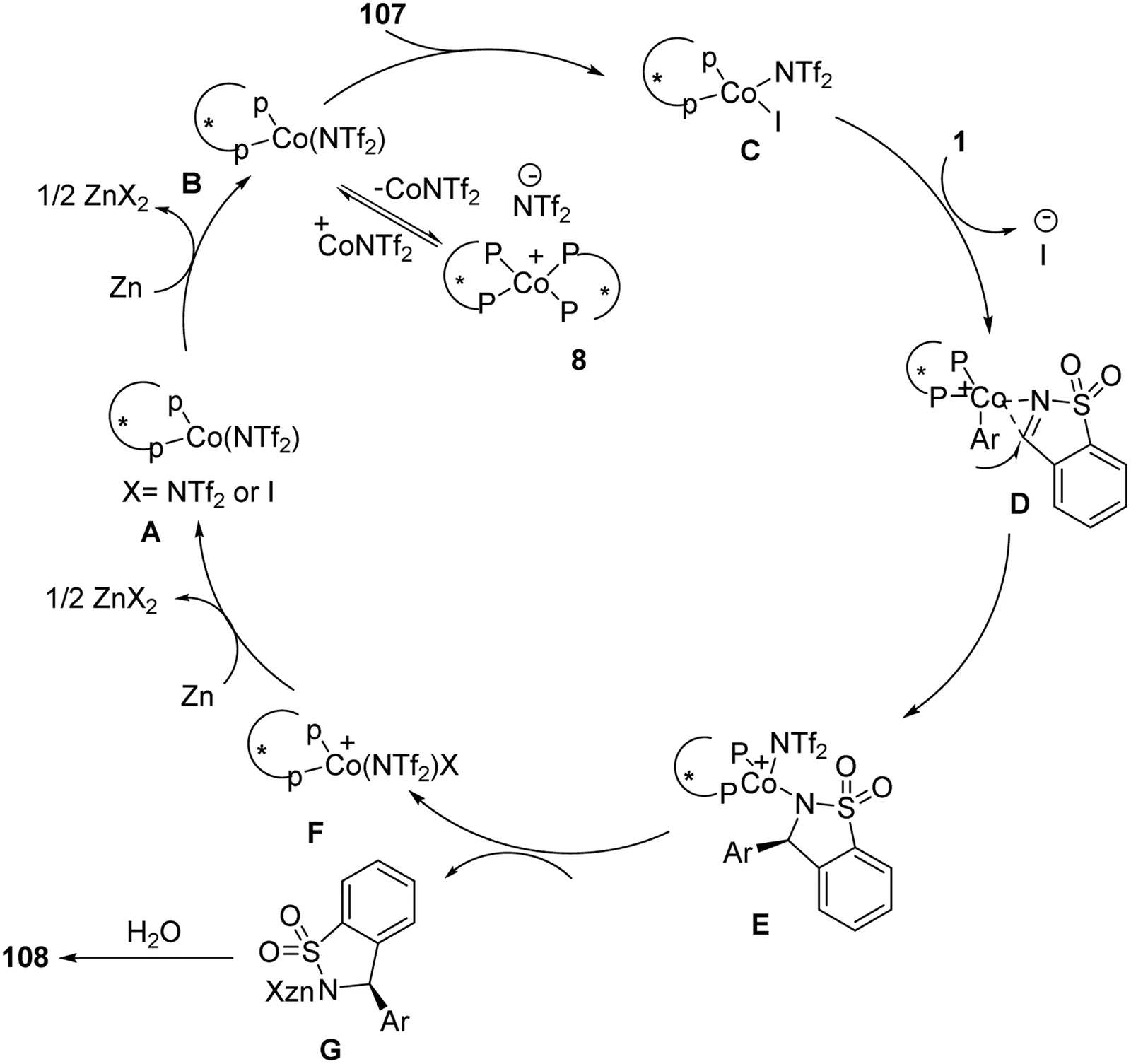
A possible mechanistic hypothesis.

Fig. 20 shows a potential mechanism that is based on findings by researchers. Co(NTf_2_)_2_ and the ligand L7 are used to form the cobalt complex A, which is then reduced by zinc by one electron to produce the cobalt complex B. Here, a coordination equilibrium is present between the species that catalyses and the more thermodynamically stable complex 8. The arylcobalt species C is produced by the oxidative addition of aryl iodide 107 to B. Compound G is released by species E during transmetallation with the *in situ* formed Zn^+2^ salt and then hydrolyses to produce product 108 as a result. When Co^+3^ species F is present, Zn engages in single electron reduction to restored catalytic species A.^188^

**Fig. 21:**
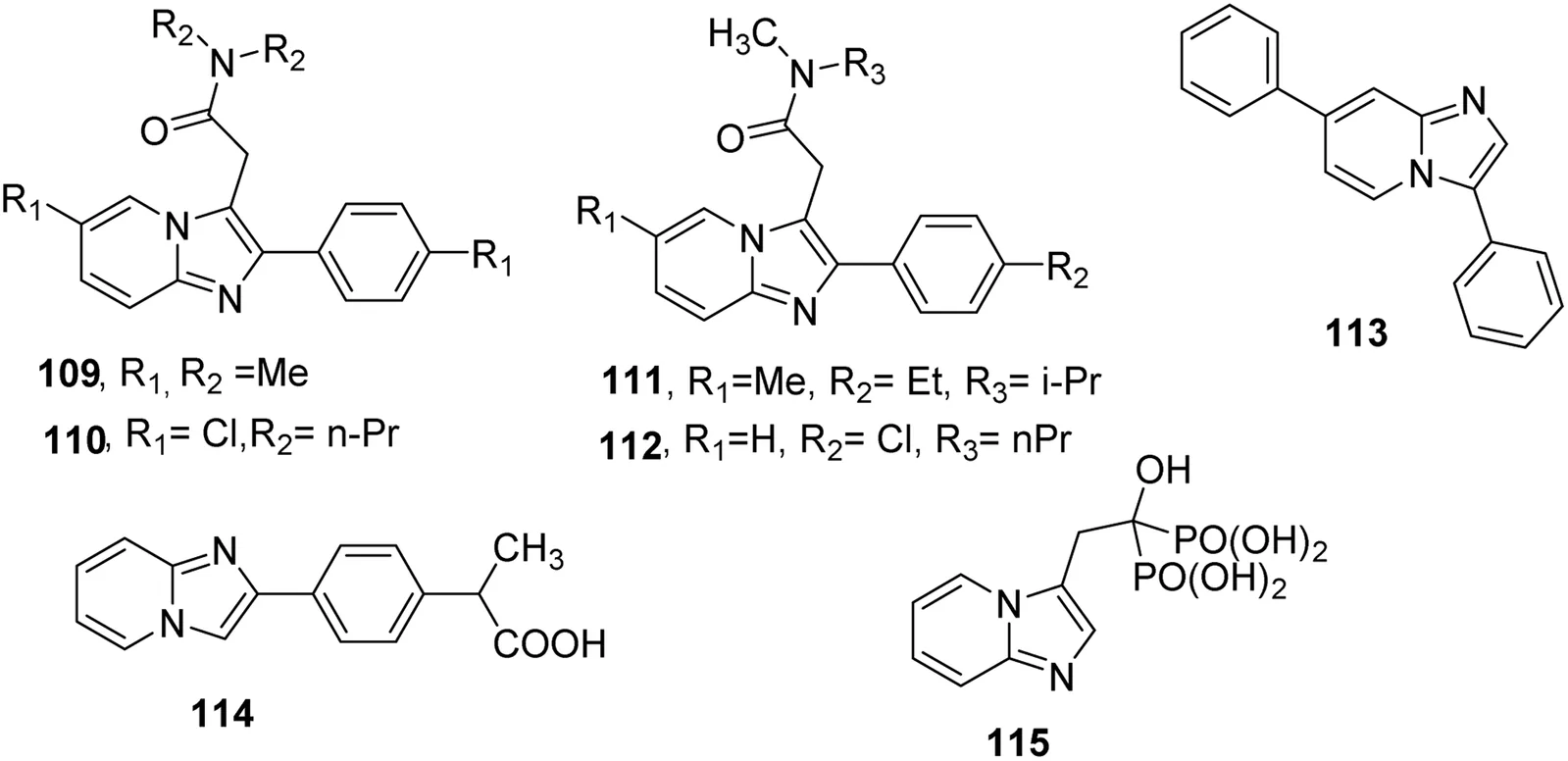
For medications and bioactive substances.

The “privileged scaffold” imidazo[1,2-α]pyridine exhibits a wide range of biological functions. Agrochemicals and other bioactive compounds contain it. Natural products and materials have practical uses.^189–192^ Particularly, substituted derivatives of these compounds demonstrated anti-inflammatory,^193^ anticancer,^194^ analgesic,^195^ antifungal,^196^ antiviral,^197^ anti-bacterial,^198^ antipyretic and other biological properties. Numerous bioactive substances and medications including alpidem 110,^199^ zolpidem 109,^199^ minodronic acid 115,^200,201^ necopidem 111,^202^ saripidem 112,^202^ miroprofen 114 and tyrosine kinase (KDR) inhibitor 113 among others include these compounds as core skeleton (Fig. 21). There are many ways for producing imidazo[1,2-α]pyridines but the most effective way is to use earth-abundant metal catalysts to synthesize arylated imidazo[1,2-α]pyridines.

**Scheme 24:**
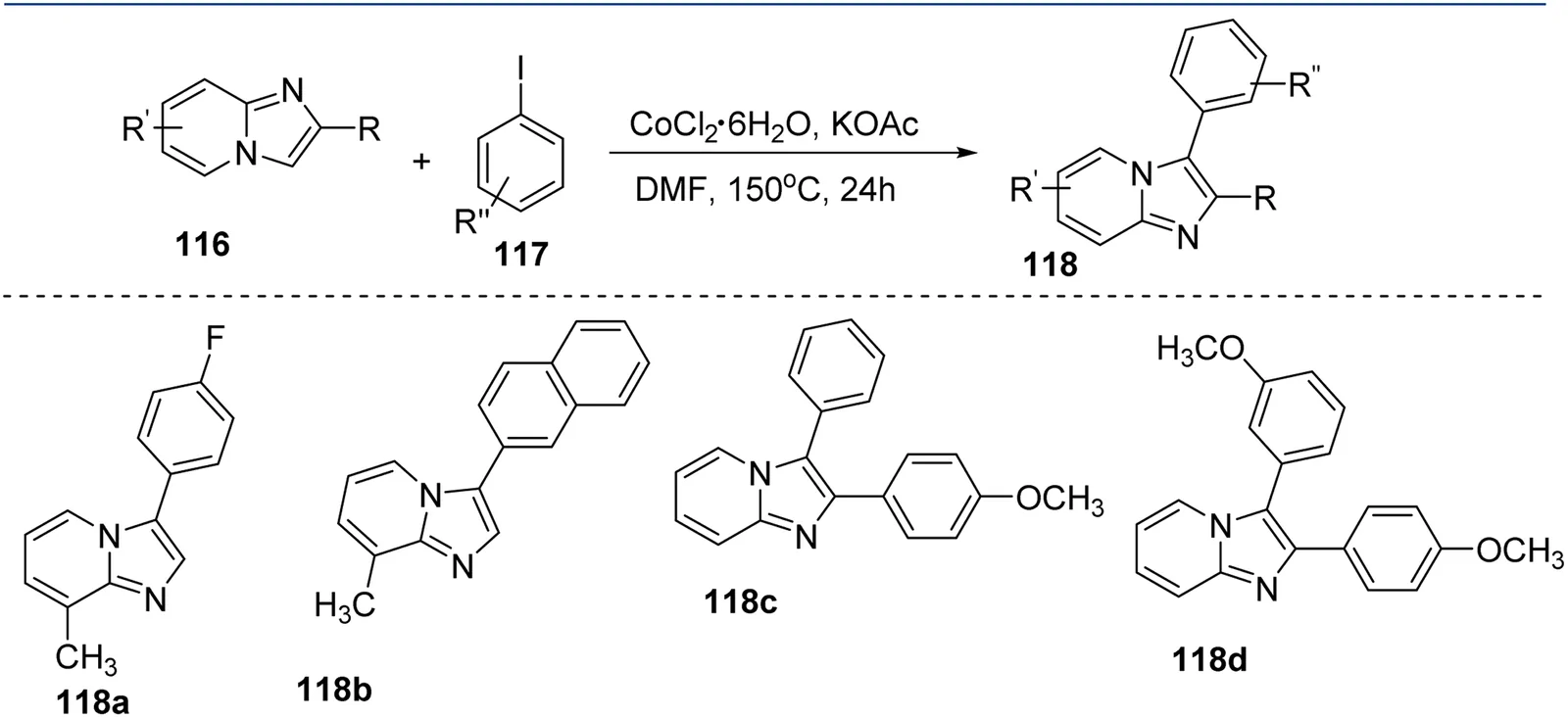
For direct arylation of various compounds.

Babar *et al.*, (Scheme 24) used a variety of substituted imidazo[1,2-α]pyridines 116 to examine the substrate scope under optimum reaction conditions. The arylated compounds 118a and 118b were produced in good yields when the electron donating groups namely –CH_3_ at the 6 or 7 or 8 position of these compounds were combined with different ArI (117). The interactions between various iodobenzenes and 2-phenyl-substituted imidazo[1,2-α]pyridines were investigated. Regardless of the substitution pattern, it provided corresponding 2,3-diphenylimidazo[1,2-α]pyridine derivatives 118c and 118d in good yields. Using this approach, the 3-naphthyl imidazo[1,2-α]pyridine 118b was effectively synthesized. In their earlier research, this compound was developed and evaluated against *Staphylococcus aureus* with a minimum inhibitory concentration (MIC) of 25 µM. The ESKAPE pathogens, which have been designated as priority pathogens by the WHO and imply a serious risk to people, include *Staphylococcus aureus*.^203^ High functional group tolerance is demonstrated by the arylation processes carried out in the presence of cobalt catalyst. The different compounds that are produced are employed as medicinal products and are biologically active. Therefore, the identification of new drugs is facilitated by the numerous arylation reactions carried out with an affordable cobalt metal catalyst, which is advantageous to humans.

**Fig. 22:**
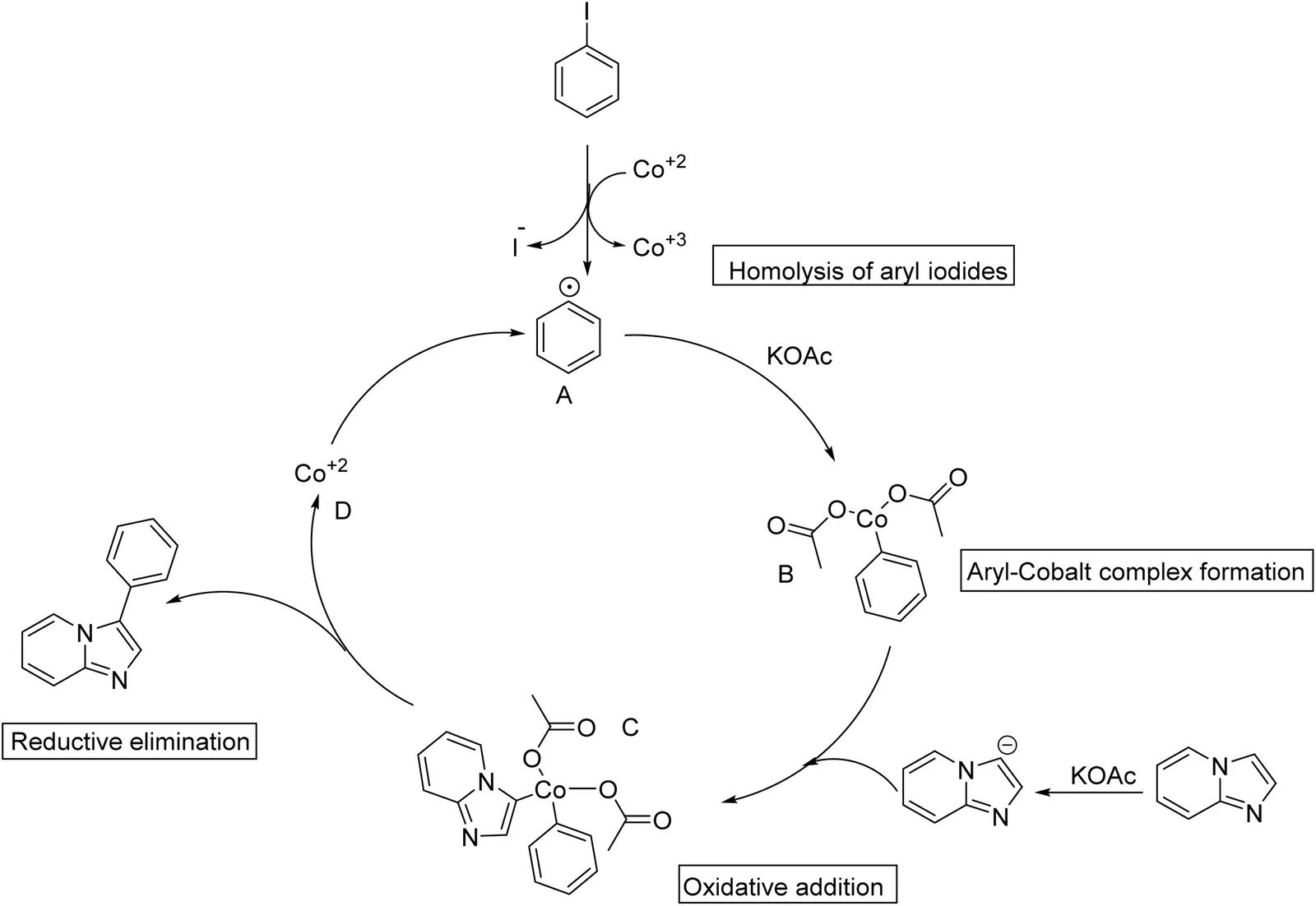
A plausible reaction mechanism.

Babar *et al.*, have developed an effective approach for the arylation of imidazo[1,2-α]pyridine with aryl iodide (Fig. 22). The formation of an Ar–Co complex B begins with the homolysis of ArI in the presence of cobalt species which produces an aryl radical. Next, species C is produced by the oxidative addition of these compounds to the aryl–cobalt complex. Finally, the reductive elimination will result in the production of cobalt^+2^ species and the 3-arylimidazo[1,2-α]pyridine.^203^

### 2.7 Isomerization reactions

There are various natural compounds and bioactive chemicals including limonene,^204^ amitorine A^205^ and phellilane L^206^ that contain chiral 1-methylcyclohexene derivatives. Limonene has a significant part in the overall synthesis of natural products as a flexible building component. Because of limitations of typical structure and derivative methods, the construction of its broad inappropriate structural library is highly desired in spite of its abundance and high availability in nature. Cobalt-catalysed one popular technique is the desymmetrizing isomerization of exo-cyclic alkenes to produce chiral 1-methylcyclohexene derivatives with good yields and enantioselectivity. Alkene isomerization is the most effective technique for specifically constructing alkenes.^207^ Several asymmetric catalytic systems were developed to attain exceptional enantioselectivity. Exo-cyclic alkene 119 is the usual substrate that had been employed to begin the investigation by researchers. Tetrahydrofuran (THF) was used as the solvent while different oxazolinyl iminoquinoline (QIQ) cobalt catalysts were tested.

**Scheme 25:**
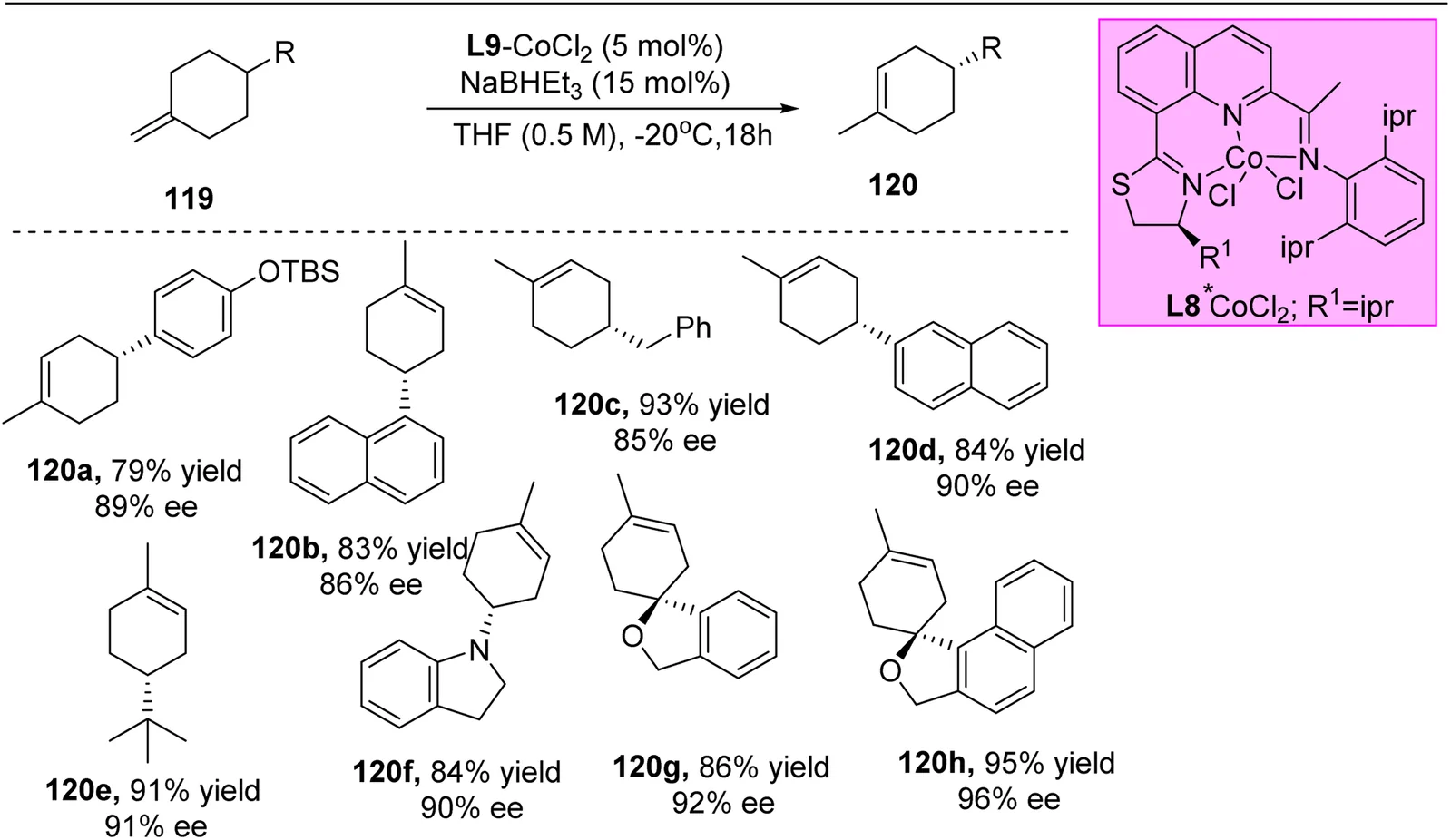
For desymmetrizing isomerization of alkene.

Liu *et al.*, investigated this isomerization's substrate scope under optimum reaction conditions. The single-crystal X-ray diffraction of 120a verified the product's exact configuration. The aryl-substituted exo-cyclic alkenes with electron-rich or electron-deficient substituents give the corresponding chiral 1-methylcyclohexene in yields of 78–95% with up to 92% *ee*. For substrates having *ortho* and *meta* substituents like methyl group, methoxy group and halogen, the reactions proceeded easily, producing a yield of up to 95% and an *ee* of up to 92%. Other polycyclic rings, such as 1-naphthyl 120b and 2-naphthyl 120d can be transformed into the desired product in 83% and 86% yields, respectively. Alkyl-substituted exo-cyclic alkene such as benzyl and ^*t*^Bu 120c and 120e substituents could smoothly undergo desymmetrizing isomerization in 91–93% yield with 85–91% *ee*. Exo-cyclic alkene 120f with heteroatom substitutions may also be tolerated in this reaction. Reactions that could generate chiral quaternary carbon centers produced the chiral spiro compounds 120g and 120h in 86% yield with 92% *ee* and 95% yield with 96% *ee*, respectively (Scheme 25).^208^

### 2.8 C–H/C–C activation reactions

Indolines are essential structural components that are frequently present in biologically active substances, including natural products and medicinal molecules.^209–212^ For example, they are present in the core skeletons of the bioactive molecules vindoline and vindorosine and serve as a synthetic precursor for the therapeutically effective cancer fighting drugs, vinblastin and vincristine.^213,214^ A subclass of these called 2-acylindolines has drawn a lot of attention because of their significance as necessary manufactured intermediates for therapeutically active substances like the potent angiotensin converting enzyme inhibitors indolapril and perindopril, the anti-cardiovascular Indolodioxane U861932 and HIV drugs like delaviridine and ateviridine.^214,215^

**Scheme 26:**
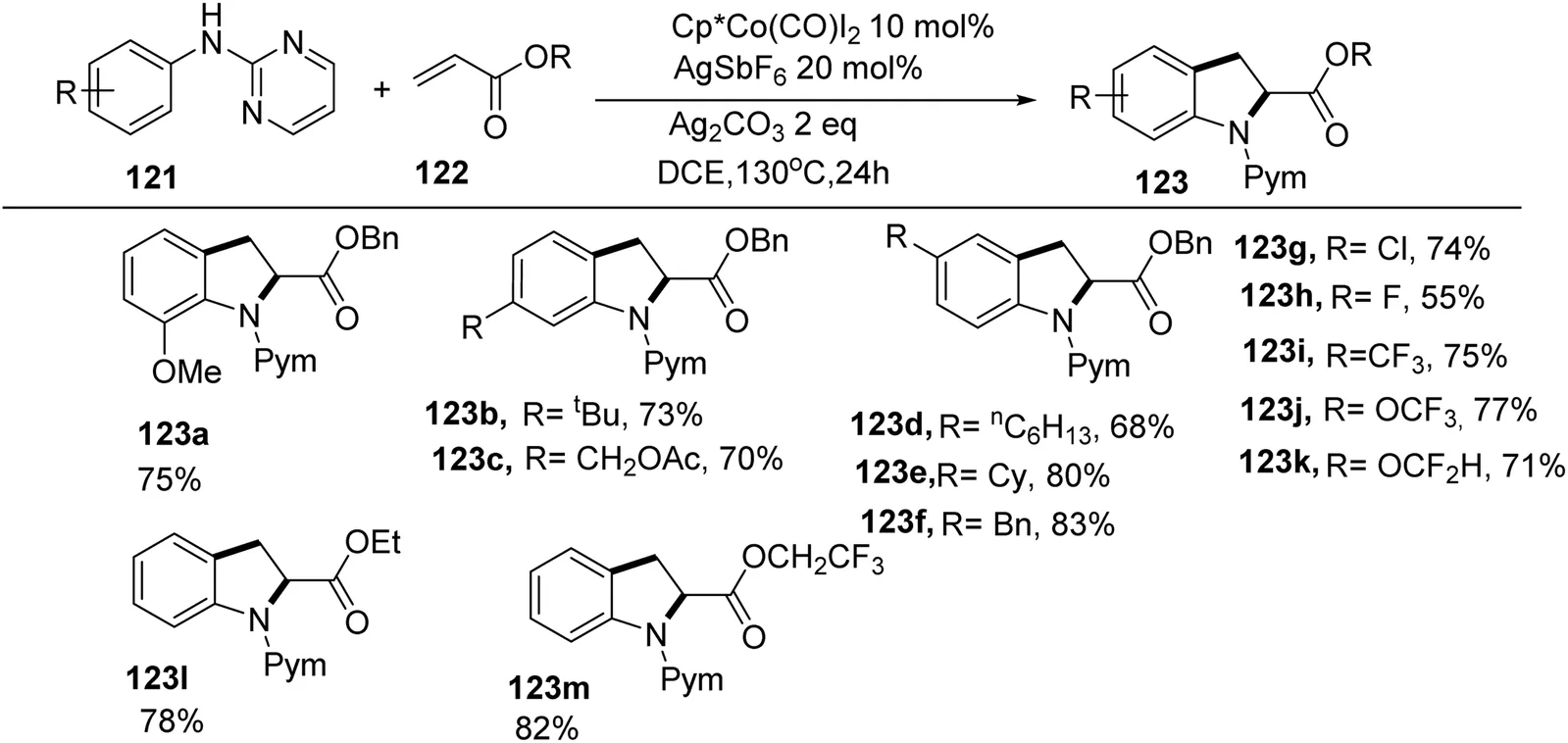
For 2-acylindolines synthesis, DCE = 1,2-dichloroethane.

The cobalt-catalysed C–H activation reaction of aniline derivatives 121 and acrylates 122 yields 2-acyl indolines 123 with a highly regioselective end product in a fast and simple manner (Scheme 26). It is possible to quickly generate functionalized 2-acylindolines using a cobalt catalyst that is abundant on earth and easily accessible pyrimidine-protected anilines. The production of 2-acylindoline products from a variety of aniline derivatives and acrylates with yields ranging from modest to good was studied by Tian *et al.*, employing optimum conditions for reaction the aniline-containing 2-OMe substrate yielded a 75% yield of annulation product 123a. The single C-6 products that were produced by *meta* substituting anilines, such as 3-^*t*^Bu and 3-CH_2_OAc, were 123b and 123c, which had yields of 73% and 70%, respectively. The *para*-substituted anilines with electron-donating substituents such as alkyl groups [4-*n*-hexyl, 4-cyclohexyl, 4-*n*-dodecyl and 4-Bn] were converted in 68–83% yields to the 2 acylindolines 123d, 123e, and 123f. Products 123h and 123g were produced when halogen groups were substituted for anilines with yields of 55% and 74% respectively. The compounds 123i, 123j and 123k*etc.* were produced by substituting anilines with fluorine-containing groups. Researchers explored further at the various acrylates. Products 123l and 123m were produced with yields of 78% and 82% using ethyl acrylates and trifluoroethyl acrylates.

**Fig. 23:**
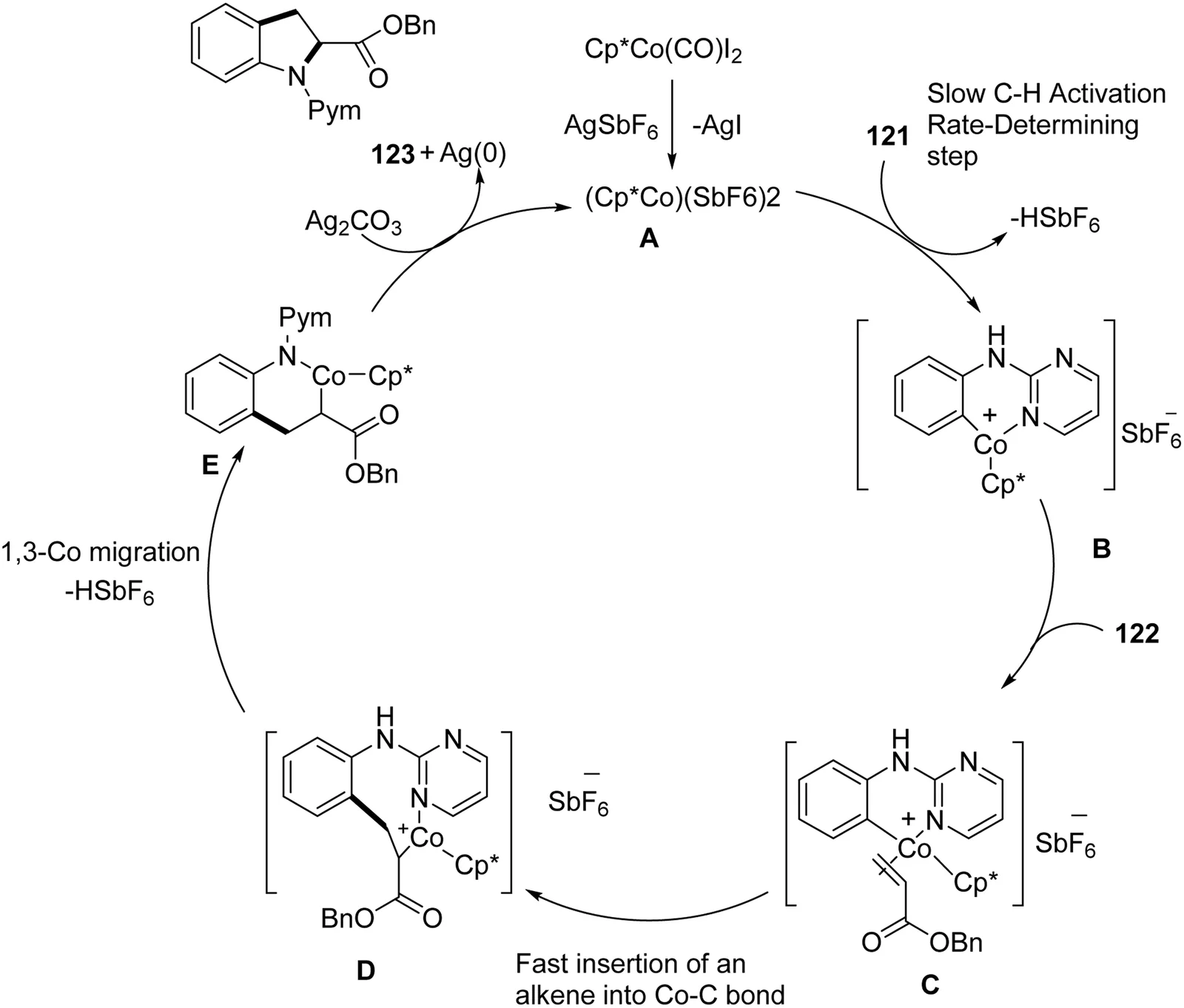
Suggested mechanism of reaction for the synthesis of 2-acylindolines.

Tian *et al.*, proposed a mechanism for this C–H activation reaction based on earlier research (Fig. 23). Cp*Co(CO)I_2_ was activated by AgSbF_6_ to produce catalytic intermediate A. The reaction's rate-determining step, intermediate B, is then produced by A gradually activating substrate 121. While acrylate 122 combined with B to form C a quick insertion into the Co–C bond created intermediate D. A 1,3-Co migration was observed in the stable D after it was obtained. By reducing and eliminating E, the product of interest 123 and Co^+1^ which Ag_2_CO_3_ oxidized to Co^+3^ were produced in between six-membered rings. The next catalytic cycle is subsequently initiated by Co^+1^.^216^

Sulfoximines function as essential structural components in a range of substances with biological activity.^217–219^ Sulfoximines and their derivatives can now be synthesized using a variety of techniques.^219–222^ The benzo-thiadiazine-1-oxides are among the many cyclic and acyclic sulfoximines that are particularly significant in medicinal chemistry.^223–226^ Conventionally, 1,2-disubstituted benzenes with the proper sulphur and nitrogen functionalities are used to synthesize benzothiadiazine-1-oxides.^227–229^

**Scheme 27:**
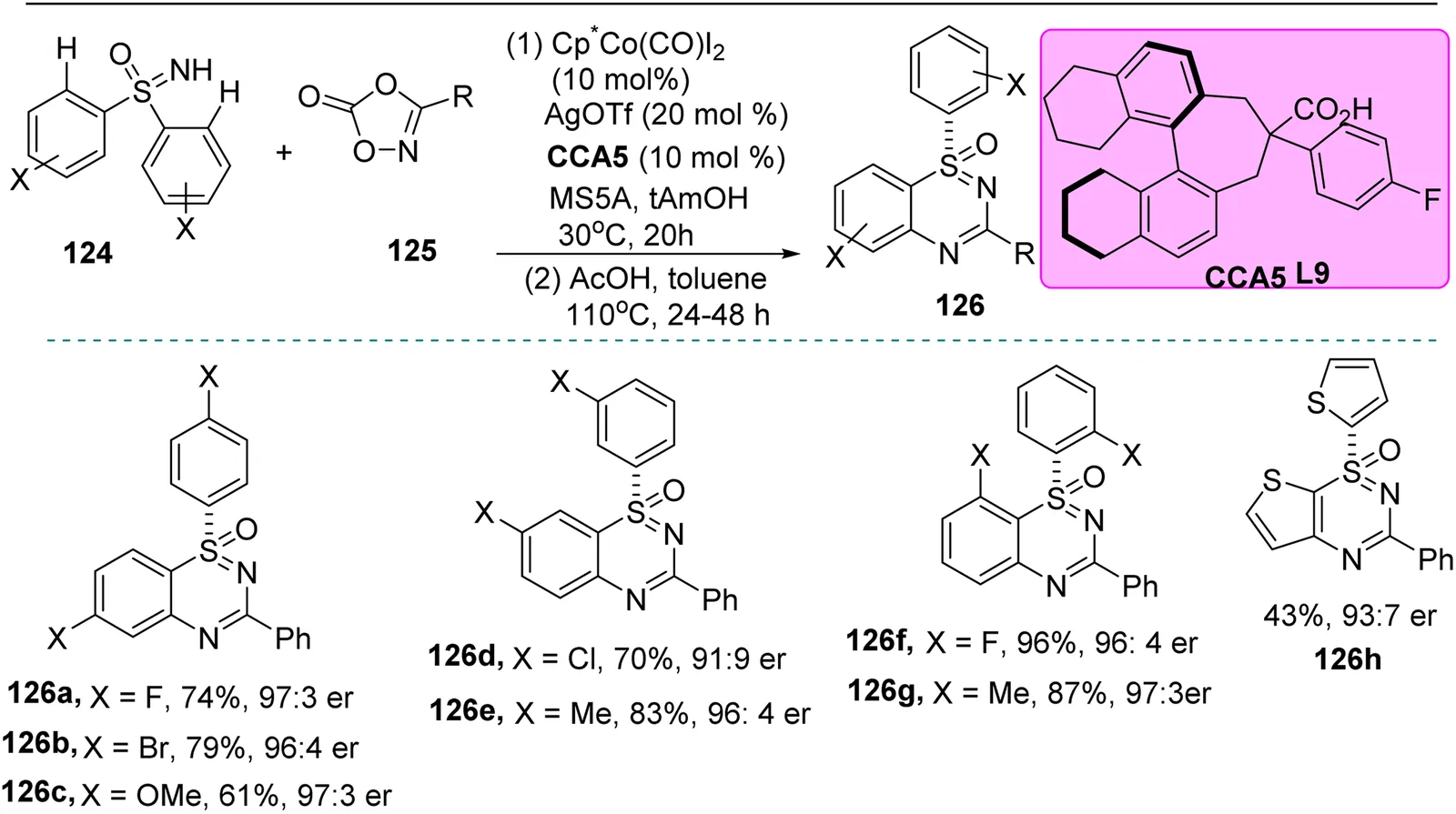
For the synthesis of benzothiadiazine-1-oxides.

Hirata *et al.*, studied the range of substrates for diarylsulfoximines under optimal reaction conditions. Except for a *m*-chlorosubstituted sulfoximine (126d, 91 : 9 *er*), the sulfoximines 124 containing an electron-donating or electron-withdrawing group at the *ortho*-, *meta*- or *para*-position provided the required products 126a, 126b, 126c, 126e, 126f, 126g with greater selectivity (96 : 4 to 97 : 3 *er*). Selective functionalization was achieved at the less sterically hindered site 126d and 126e using *meta*-substituted sulfoximines. With a modest yield and selectivity 126h a sulfoximine with two 2-thienyl groups produced the target product as an invincible combination with an overly-amidated product (Scheme 27).^230^

**Fig. 24:**
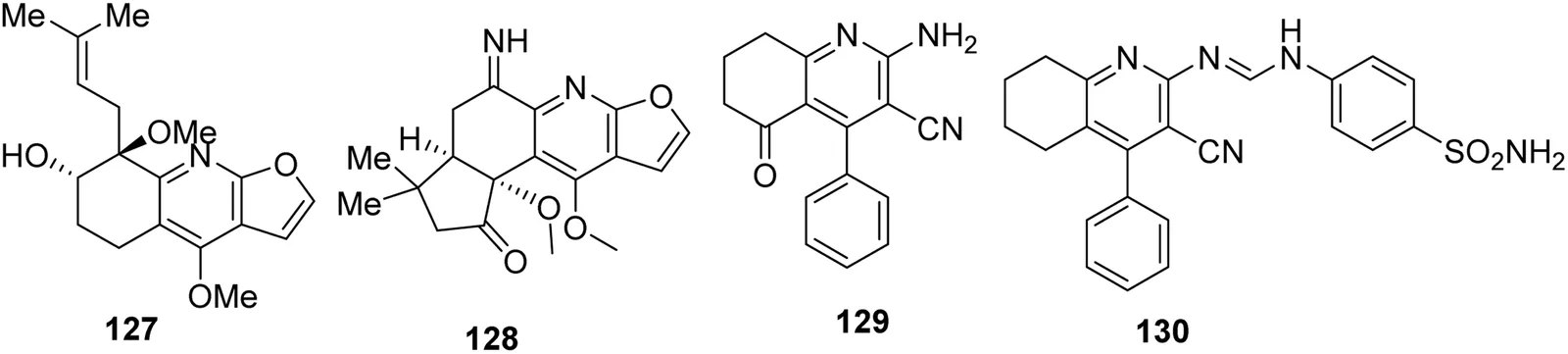
A tetrahydroquinoline moiety contained in a bioactive molecule, as well as other organic compounds.

The multisubstituted pyridine (tetrahydroquinoline) is found as a key structural component in many naturally occurring biologically active compounds such as haplophyllidine 127 (ref. 231) and megistosarcimine 128 (ref. 232) and heterocycles containing nitrogen. Compounds containing the tetrahydroquinoline moiety exhibit biological activity like antifungal 129 (ref. 233) and anticancer 130 (ref. 234) properties (Fig. 24). Bioactive compounds are dehydropregnenoloned using this annulation technique. Co(iii)-catalyst has been used to create multisubstituted pyridines in an effective and appropriate manner .

**Scheme 28:**
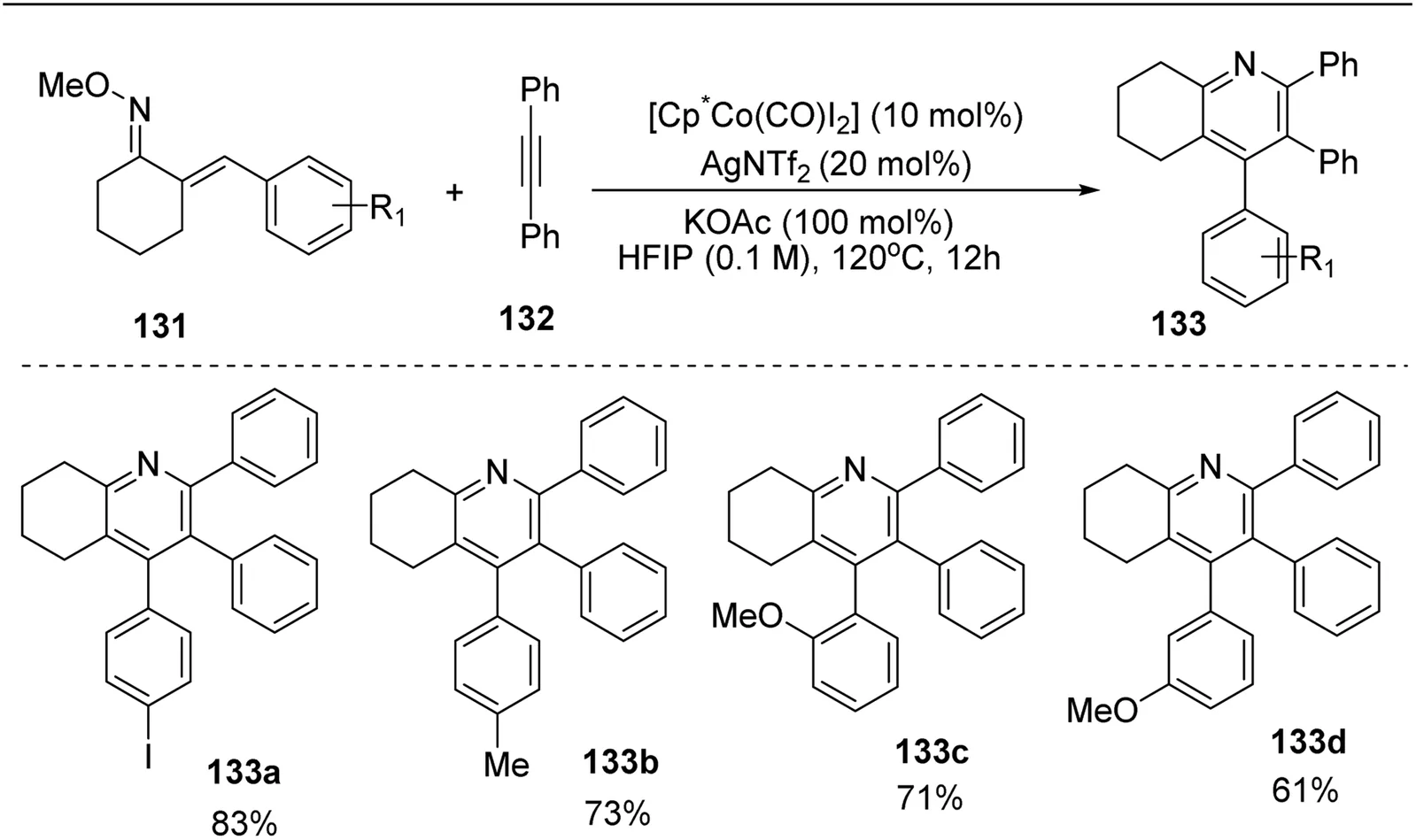
For synthesis of multisubstituted pyridine, HFIP = hexafluoroisopropanol.

The substrate scope of the general reaction has been investigated by Mohanty *et al.*, under optimal reaction conditions. The yield of the intended product from the unsubstituted oxime ether is excellent (88%). For this reaction, the *para*-halogen substituted α,β-unsaturated oxime ether (–I) is appropriate and it produces the corresponding product 133a in excellent yield. When an oxime ether has an electron-donating group at the *p*-position (–Me), a high yield of the desired product 133b is produced. However, when an electron-withdrawing group is present a low yield of the comparable product is produced. Regarding *ortho*-Br-substituted oxime ether, very little product is generated, this may be because the bulky bromide group close to the site of reaction acts as a steric hindrance. However, the desired product 133c is produced in 71% yield when methoxy group is present at the *ortho*-position. A 60% yield of the analogous product 133d is produced using the *meta*-substituted oxime ether (*m*-OMe) (Scheme 28).^235^

**Scheme 29:**
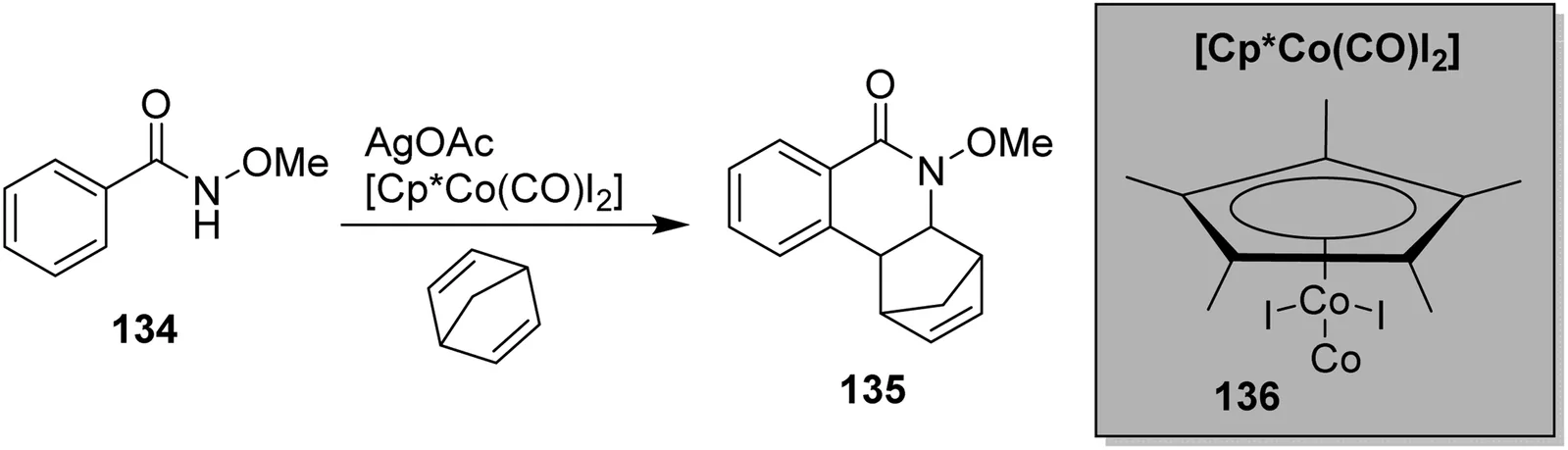
Synthesis of dihydroisoquinolones *via* cobalt catalyst.

Vega *et al.*, synthesized 3,4-dihydroisoquinolones 135 using silver acetate (AgOAc) as an oxidant and a cobalt metal complex, [Cp*Co(CO)I_2_] 136 to activate a previously inert C–H bond, changing it to a C–C bond and concurrently generating a heterocycle composed of methoxybenzamide and the strained hydrocarbon, norbornadiene (Scheme 29).^236^ Because it can improve features like charge distribution, lipophilicity and metabolic stability among others, the addition of fluorine to ordinary organic compounds is extremely important for medical purposes.^237–240^ Over 20% of commercially available agrochemicals and authorized therapeutic medicines were fluorinated organic molecules.^239,241^ The significance of organofluorine compounds as well as the fact that homoallylic alcohols are frequently found in many bioactive compounds and are very useful building blocks in complex molecule synthesis.^242,243^ Therefore, an effective fluorination method for replacing the C–H bond of homoallylic alcohols with a C–F unit had been established.

**Scheme 30:**
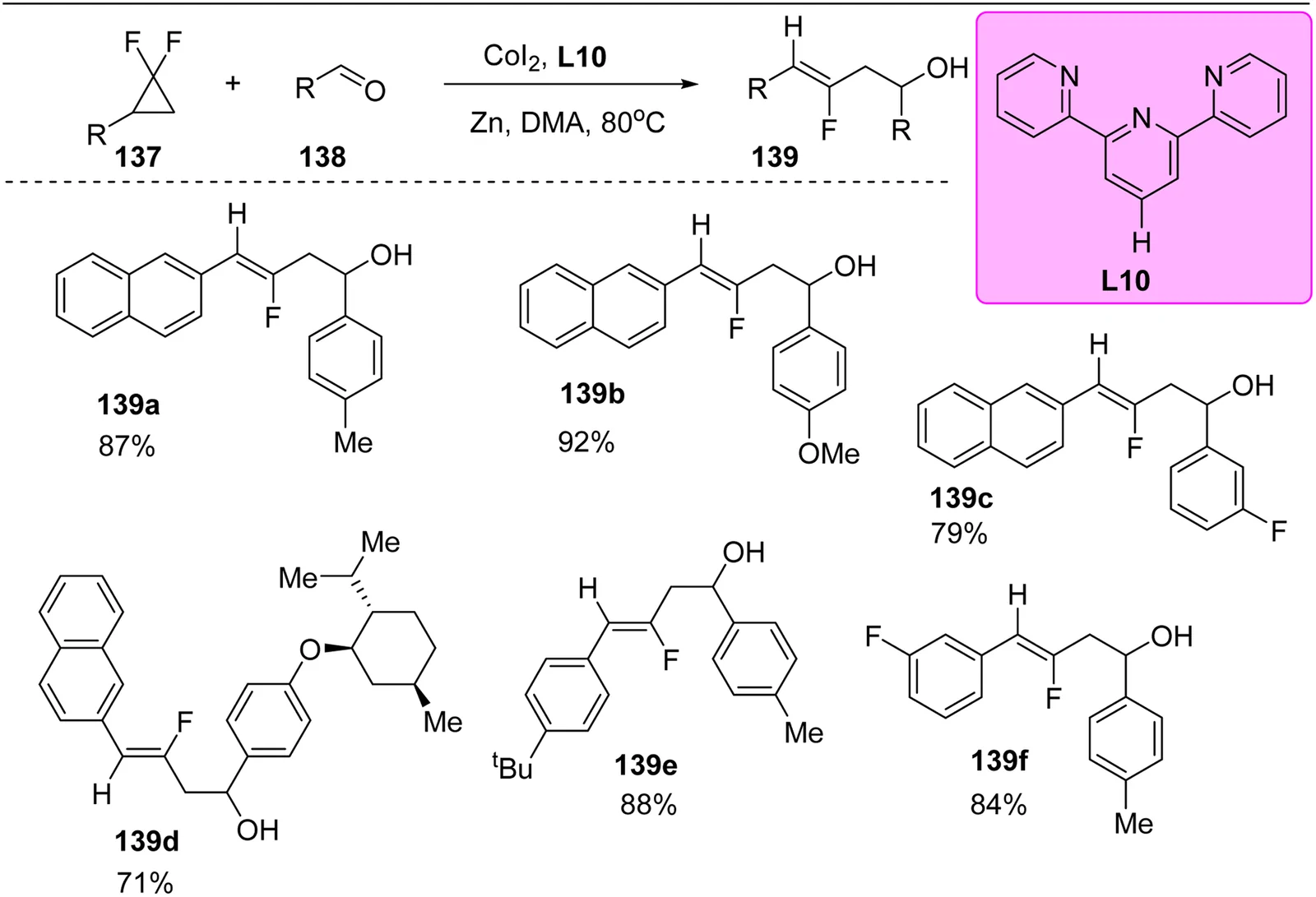
For carbonyls undergo fluoroallyllation through C–C activation.

Ai *et al.*, used various substituents on the functional groups of aldehydes to examine the substrate scope under optimum reaction conditions. The yield and *Z*/*E* selectivity were found to be slightly affected by altering the electron-donating groups such as –CH_3_ and –OCH_3_ on the *p*-position of the phenyl ring 139a and 139b. On the other hand, phenylaldehyde containing an *N*,*N*-dimethyl group produced increased *Z*-selectivity though with a low yield which is probably due to more effective thermodynamical control because of the low reaction rate given by the aldehyde's weaker electrophilicity. Numerous substituents including fluorine and those at the *m*-position of the benzene ring were likewise tolerated and produced the desired results 139c in good yields. The process used arylaldehyde generated from l-menthol as a substrate and produced the desired product 139d with a 71% yield. Unfortunately, the side reaction of aldehydes being converted to alcohols prevents 4-formylbenzonitrile, methyl-4-formylbenzoate, 4-(trifluoromethyl)benzaldehyde and cinnamic aldehyde from being compatible with the catalytic system. The substrate scope with regard to *gem*-FCPs was then investigated. On *gem*-difluorocyclopropanes, a variety of electronic or steric-biased substituents could be introduced with ease. On the benzene rings, both electron-donating groups like ^*t*^butyl and electron-withdrawing groups like fluoro were permitted, producing the corresponding products 139e and 139f. However, the catalytic reactivity of the alkyl-substituted *gem*-FCPs are not strong.^244^ High regioselectivity has been observed in the C–H activation reactions carried out under cobalt catalysis. This reaction's drawback is that it is not atom-economical and produces less regioselectivity when other metal catalysts such as Pd and Cu are used (Scheme 30).

**Fig. 25:**
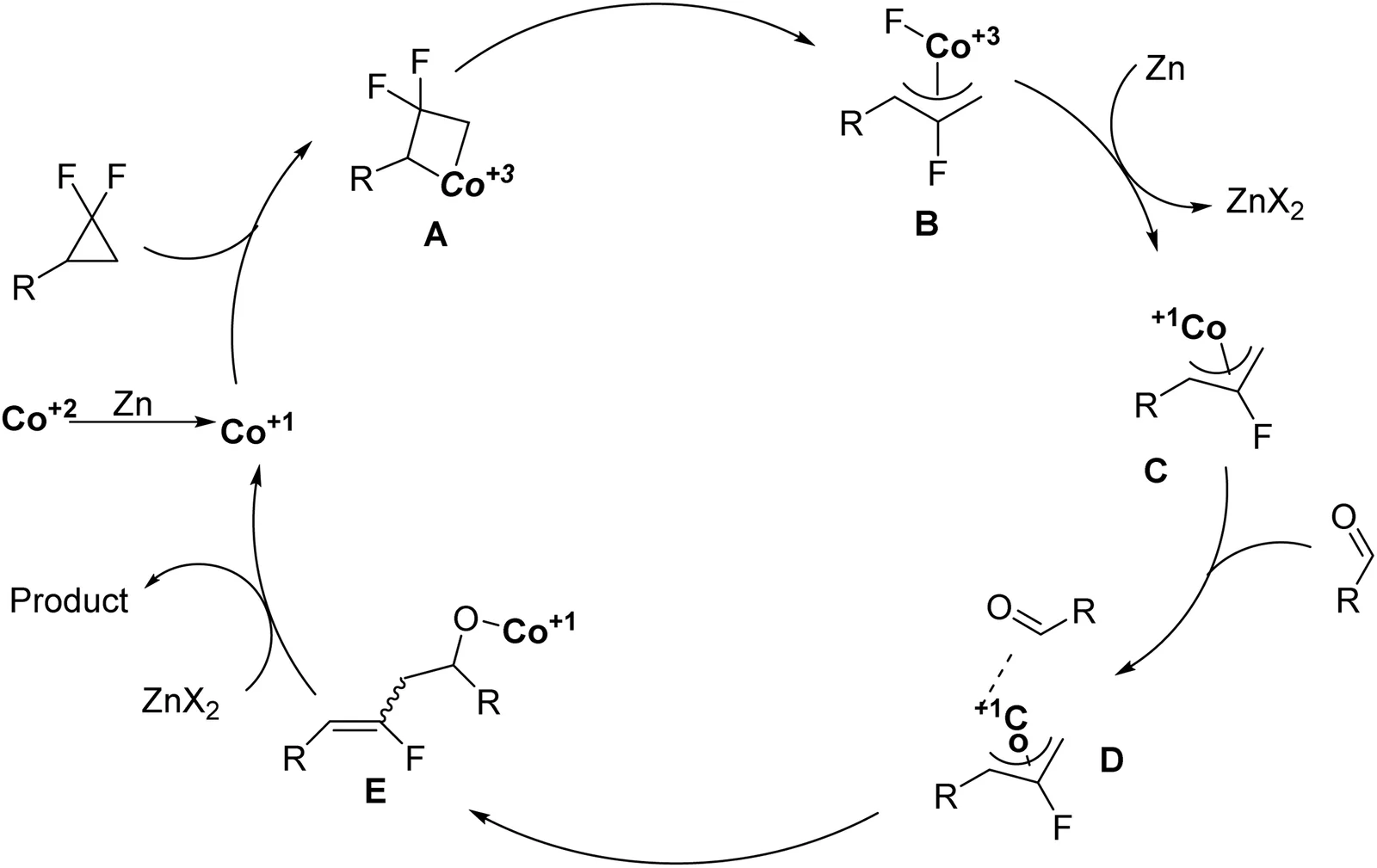
For possible reaction mechanism.

In Fig. 25, a proposed mechanism has been described. Following the zinc additive's initial reduction of Co^+2^ to Co^+1^, a four-membered alkylcobalt(iii) species A was produced by the C–C oxidative addition of *gem*-FCPs which was then followed by strain-release-driven β-F elimination. After zinc reduction, the nucleophilic intermediate of allylcobalt(i) was produced by the Umpolung reaction which took place with the electrophilic allylcobalt(iii). Finally, the substituted fluorinated homoallylic alcohol and Co^+1^ species were obtained by simply adding the nucleophilic intermediate to the aldehyde and protonating it. The low *Z*/*E* selectivity during the γ-addition phase was most likely mediated by the π–σ–π interconversion process between *syn*-π-Co^+1^ complexes and anti-π-Co^+1^ complexes.^244^

### 2.9 Radical hydroamination reaction

In the synthetic community, the hydroamination of alkenes in the presence of a suitable catalyst is one is among the effective and swiftly applied techniques for developing compounds that contain nitrogen which are extensively distributed in many biologically active natural products and pharmaceutical agents.^245–249^ Due to its high chemoselectivity, vigorous reactivity, excellent functional group sufferance and straightforward construction of a congested carbon centre, alkenes underwent radical hydroamination which was facilitated by a transition metal has emerged as a multifaceted and captivating method for developing the necessary C–N bonds in recent years.^250,251^ This method has been employed for making chiral amines in large numbers.

**Scheme 31:**
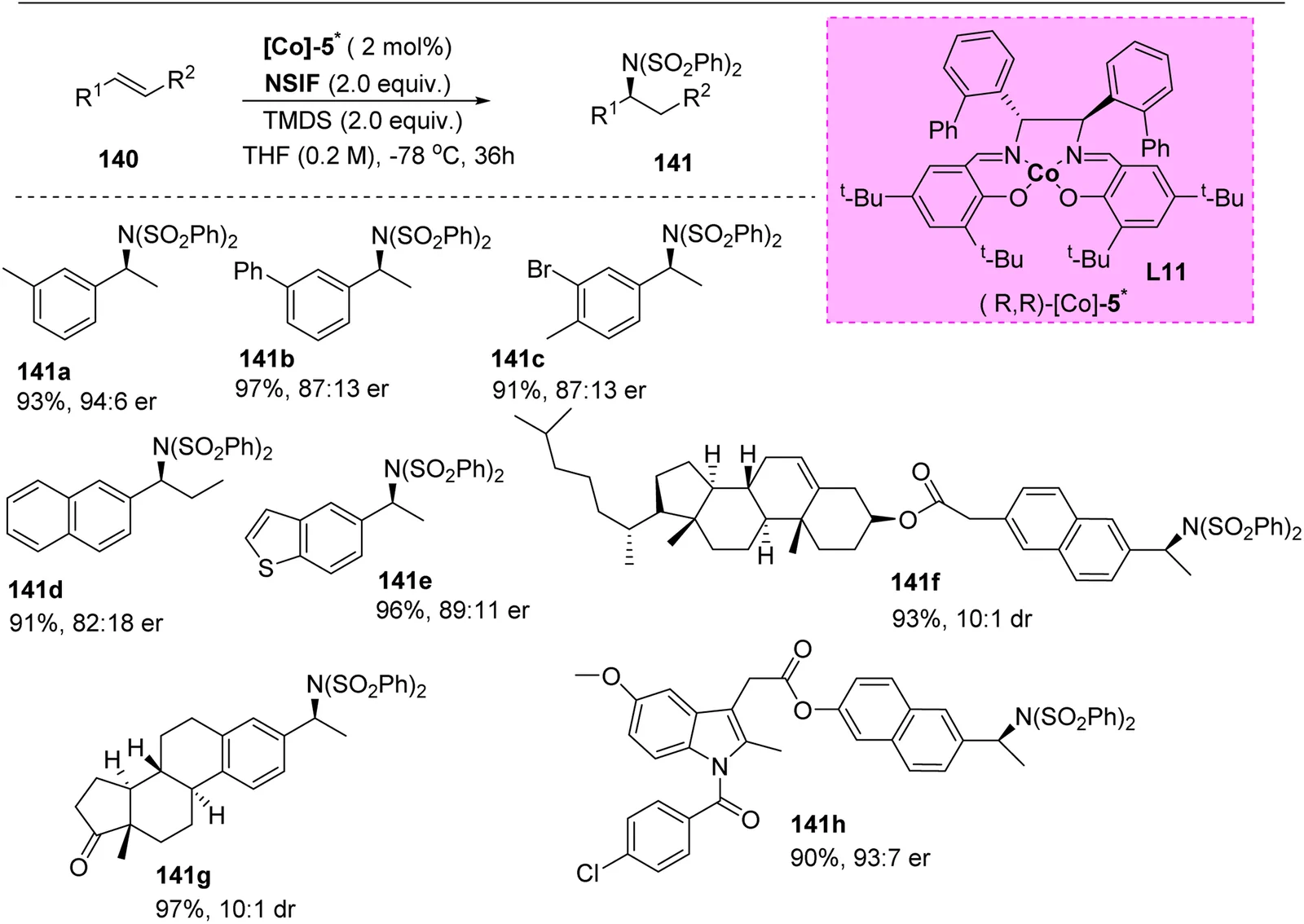
For radical hydroamination of alkenes, TMDS = tetramethyldisiloxane.

Qin *et al.*, looked into the range of substrates for various alkenes 140 using optimum reaction conditions (Scheme 31). Despite having similar reactivity to *ortho*- and *para*-substituted styrenes, the substituents on the *meta*-position of the aromatic ring in styrenes were useful for improving enantiocontrol. In order to produce their corresponding chiral amines 141b and 141c with outstanding yields and good levels of enatioselectivities several styrenes containing multiple substituents (for example, -aryl and –Br) at *meta* position have been investigated. Single-crystal X-ray diffraction was specifically used to identify the absolute configuration of product 141a. This transformation of the internal alkene could result in a 91% yield of the chiral amine 141d that corresponds to it. Numerous heteroaryl substituted alkenes, including 5-vinylbenzo[*b*]-thiophene, hydroaminate easily with great yield and acceptable enantiocontrol 141e. This approach works well for the final stages of functioning of complex molecules, as evidenced by the easy conversion of some bioactive molecules, including cholesterol and the structurally more complex estrogen and indomethacin, to their corresponding chiral amines 141f, 141g and 141h.^252^

**Fig. 26:**
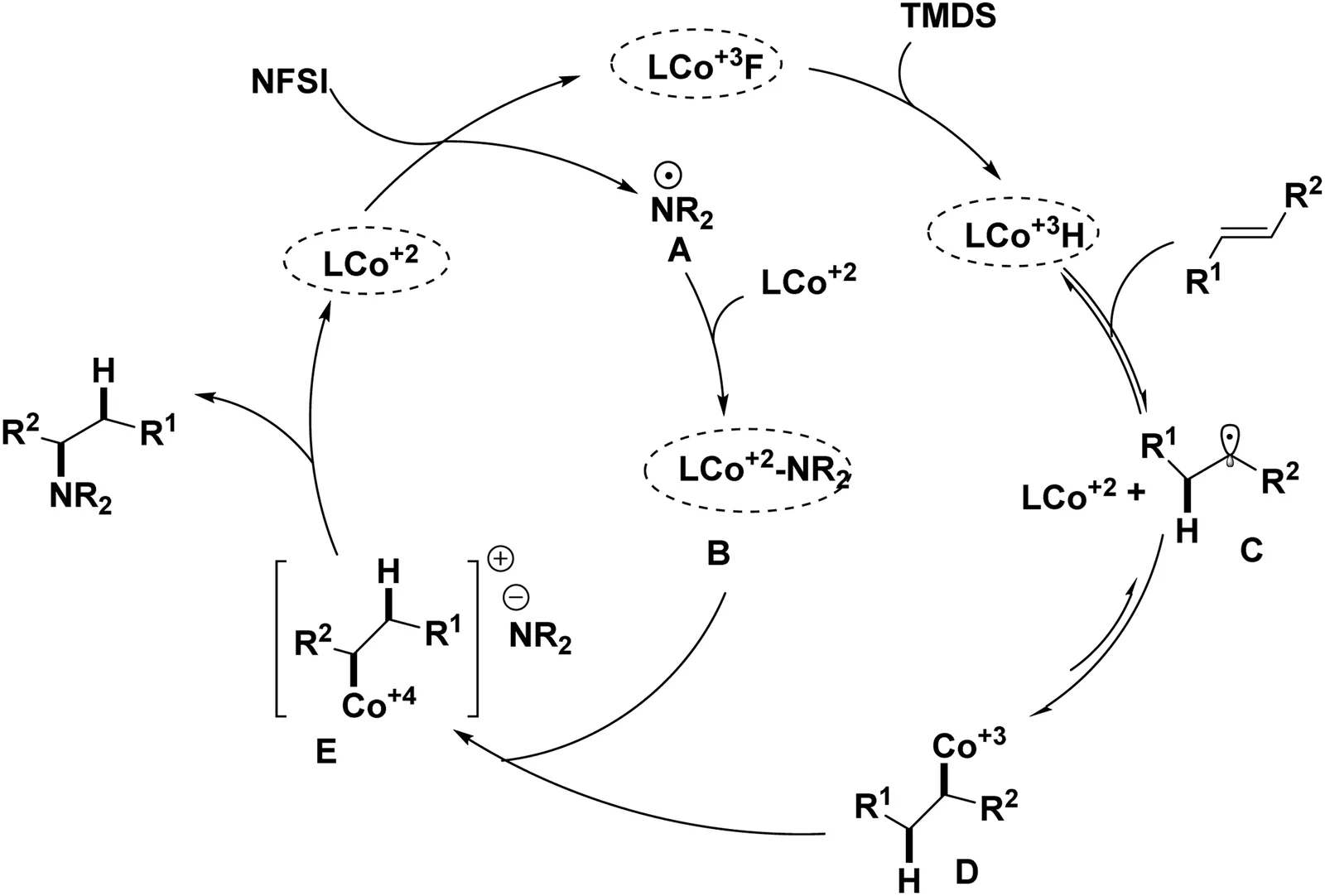
Proposed mechanism for radical hydroamination of alkenes.

Fig. 26 depicts a potential Co^+3^H-catalysed radical hydroamination route. The LCo^+2^ species are first oxidized by NFSI to produce the LCo^+3^F species and benzenesulfonimide radical A. Next, the LCo^+3^F species are transmetalate using TMDS to produce the LCo^+3^H species. High-resolution mass spectrometry (HRMS) has identified the LCo^+3^-NR_2_ species B as the result of the radical A being trapped by Co^+2^ species. The alkylcobalt(iii) species D would therefore be provided by and LCo^+3^H-mediated reversible HAT with alkene *via* the intermediary of C. The crucial radical cationic alkyl CO^+4^ intermediate E would then be formed *via* a single electron transfer between the resulting alkylcobalt(iii) complex D and Co^+3^-NR_2_ species B with the counter anion -NR_2_.^252^

**Fig. 27:**
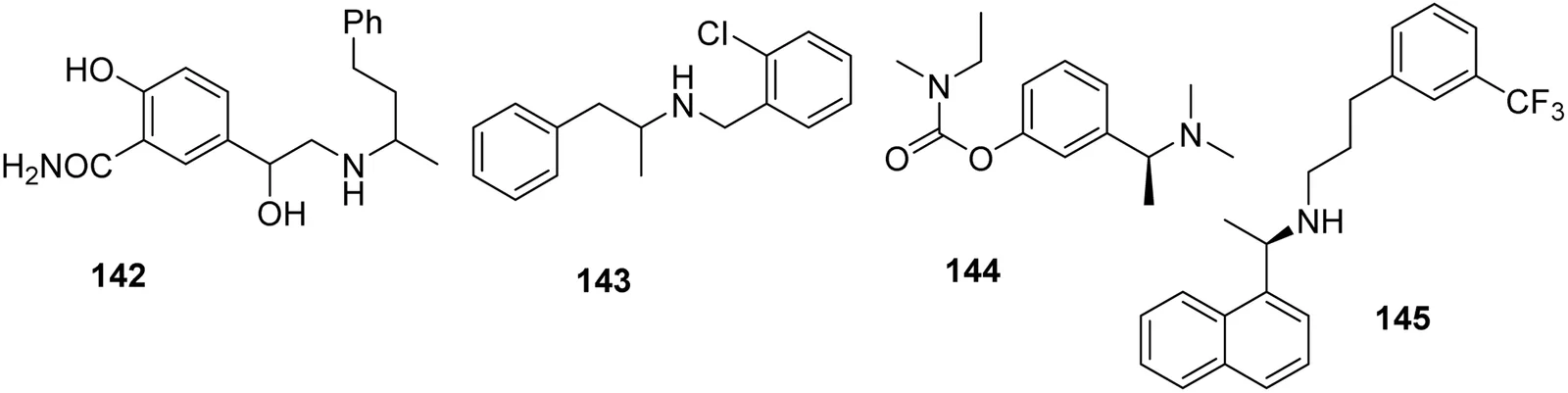
Some examples of biologically and pharmaceutically active amine derivative chemical compounds.

Numerous natural products, medicines, and biologically active substances contain amine and its derivatives (Fig. 27).^253,254^ Therefore, it is particularly interesting to synthesize amines and their derivatives from widely available starting materials. One of the most effective ways to create compounds containing nitrogen is by hydrofunctionalizing easily accessible alkenes containing nitrogen sources using metal catalysts. Among the several activation techniques utilized for the alkene hydroamination, the metal-catalysed hydrogen atom transfer (HAT) reaction demonstrates excellent markovnikov selectivity and chemoselectivity.

**Scheme 32:**
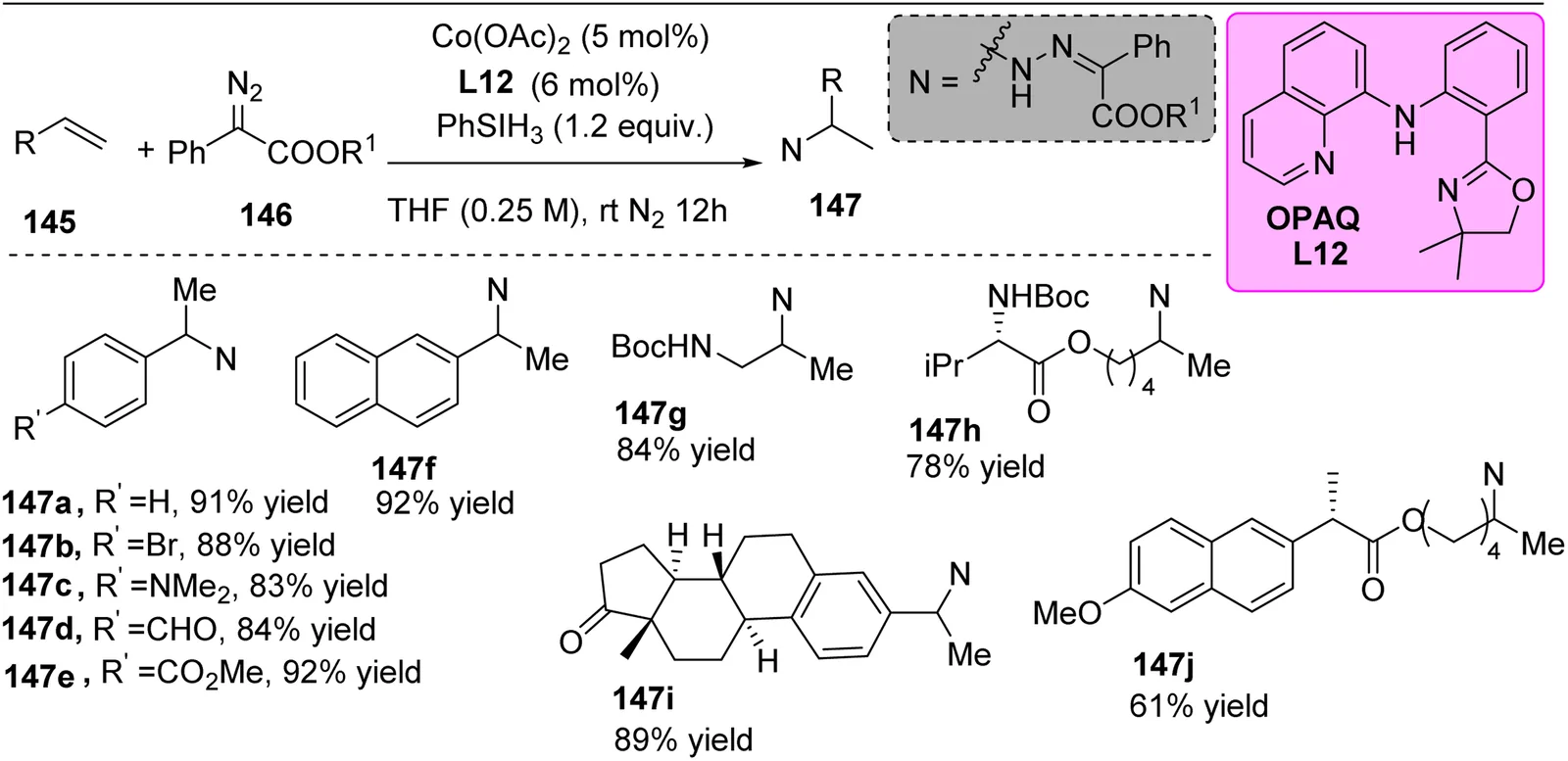
For alkenes subjected to radical hydroamination.

Under optimum reaction circumstances, Shen *et al.*, examined the substrate scope (Scheme 32). Various styrenes having *para*-, *meta*- or *ortho*-position electron-donating or electron-withdrawing substituents on the phenyl ring underwent the reactions to produce the corresponding benzylamine derivatives 147a, 147b, 147c, 147d and 147e in yields ranging from 83 to 92%. In a 92% yield 2-naphthyl could be changed into the corresponding product 147f. Alkyl-substituted terminal alkenes may react without difficulty. The diamine 147g might be produced by reacting the derivatives of allylic amine in an 84% yield. Naturally occurring substances with a terminal alkene moiety like amino acids and estrogen, may be used to produce products 147h and 147i in yields of 78 and 89% demonstrating the suitability of this technique for the late-stage functionalization of complex compounds. Naproxen an anti-inflammatory medication was transformed from a terminal alkene to 147j in a 61% yield.^255^

**Fig. 28:**
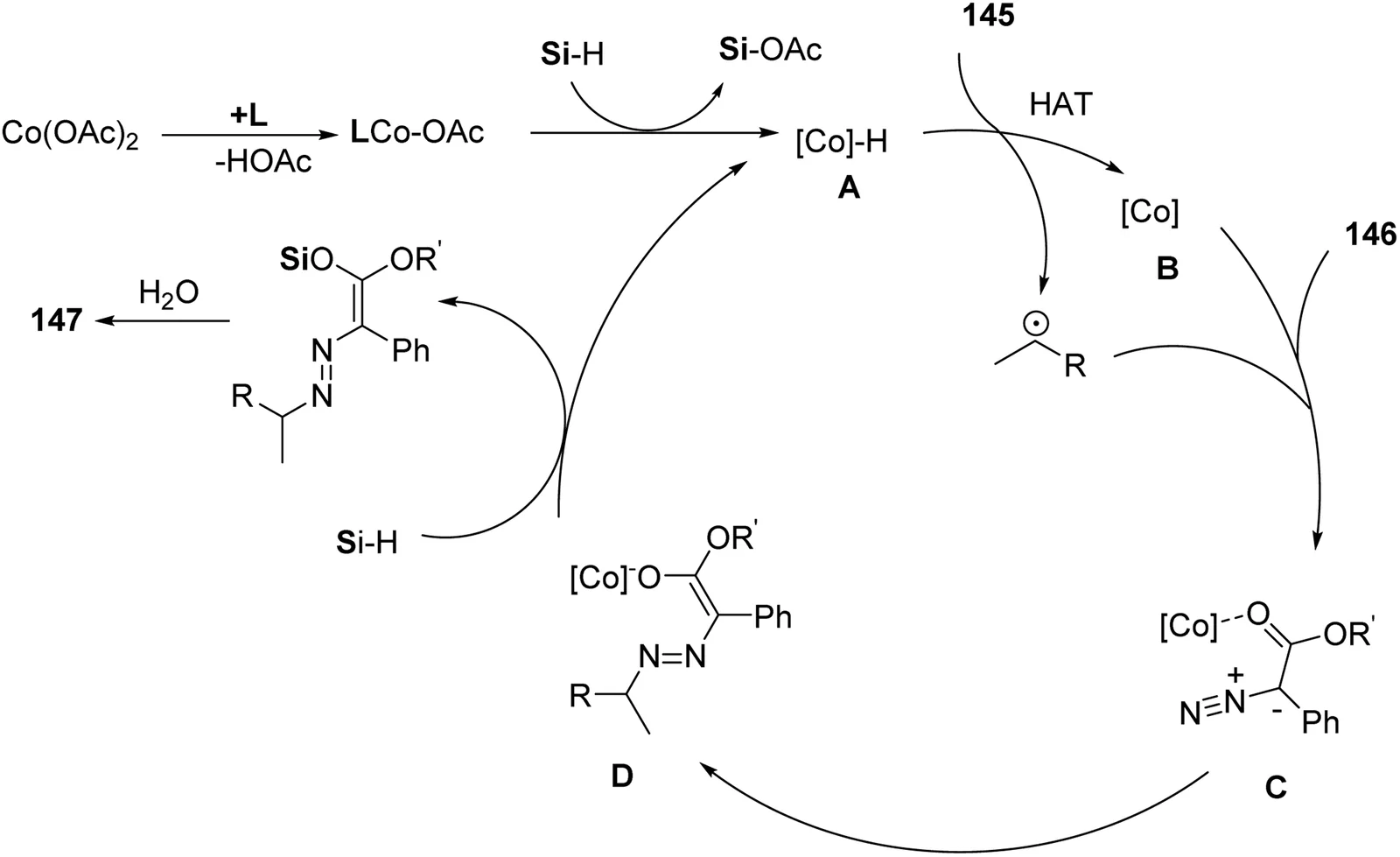
A proposed reaction mechanism.

A potential mechanism was depicted in Fig. 28 based on experimental research and previously published literature. The metal hydride HAT method can produce the carbon radical intermediate and cobalt species B from the cobalt hydride species A that is produced when Co (OAc)_2_ reacts with ligand and silane. The chemical valence of cobalt was inconsistent because of the ligand's potential redox non-innocent feature. In order to produce the cobalt–carbon species C, the cobalt species B may combine with the diazo compound and go through one electron oxidation with the carbon radical intermediate. The cobalt–carbon species C may then go through alkyl group migration from the cobalt to the nitrogen atom to produce the cobalt oxide species D. It was impossible to rule out the probability that the cobalt coordinated diazo complex was directly attacked by a carbon radical. The cobalt species D might interact with hydrosilane to produce the cobalt hydride species A again and the intermediate vinyl silyl ether which could then go through hydrolysis and isomerization to produce the final product.^255^

Nitrogen^241,253,256^ and fluorine^257–259^ are always existing in both manufactured intermediary substances found in organic compounds and bioactive active substances used in biological research. Thus, a result of highly effective and focused amination^260–262^ and fluorination^263–265^ methods is important. The *N*-fluorobenzene sulfonimide (NFSI), the most versatile amination agent,^266,267^ fluorination agent,^268,269^ as well as amino-fluorination agent^270,271^ has been utilized for this purpose. The highly effective method for differential amino-fluorination and di-amination of styrene with cobalt as the catalyst has been developed.

**Scheme 33:**
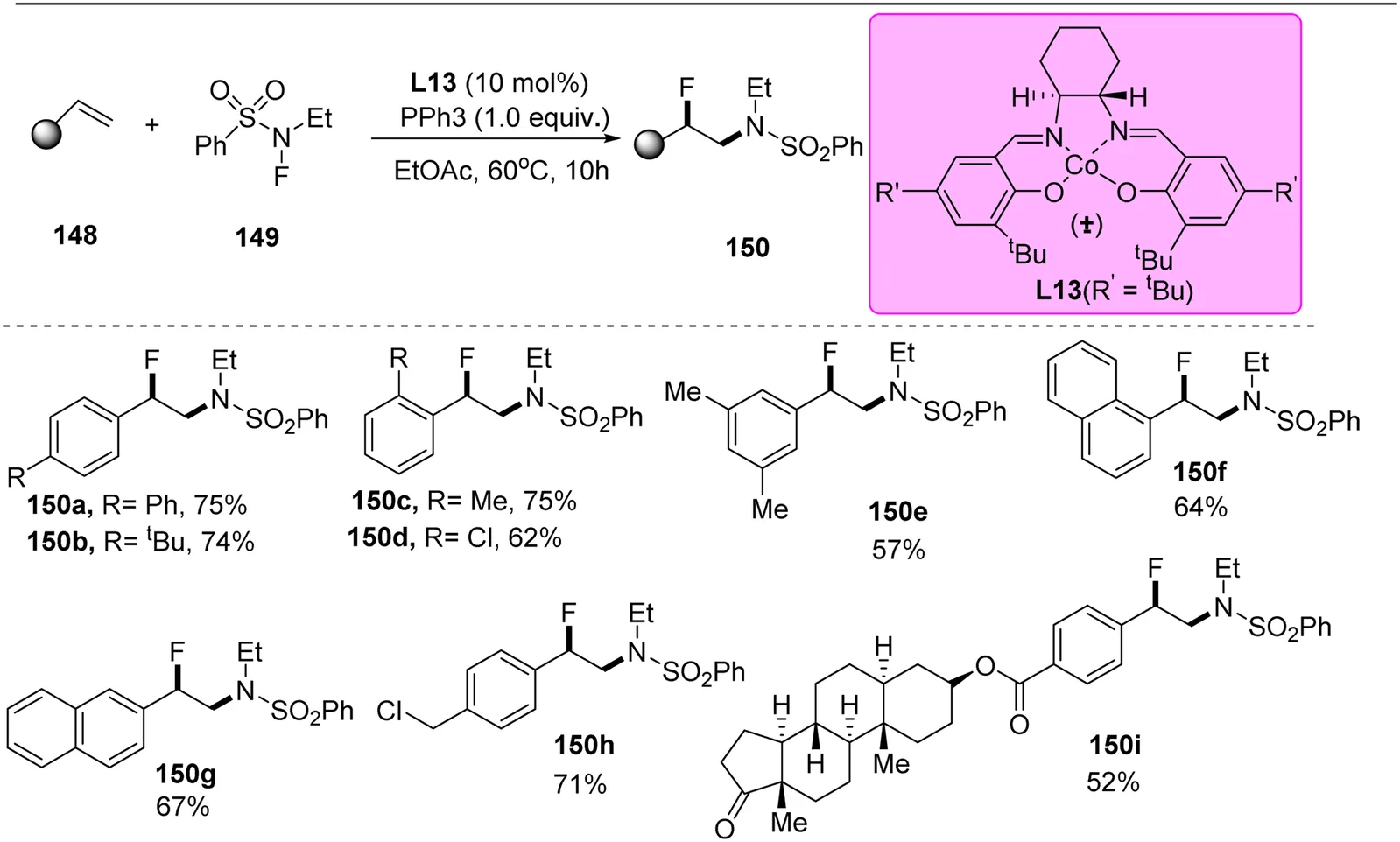
With regard to styrene di-amination and amino-fluorination.

Guo *et al.*, has been explored the range of the intermolecular amino-fluorination 149 reaction of alkenes 148 catalysed by cobalt under optimum reaction conditions (Scheme 33). The corresponding product 150a, 150b, 150c, 150d, 150e are produced when substituents that donate and withdraw electrons are present at the *ortho*, *meta* or *para* positions of styrenes. These reaction conditions are applied to both the α- and β-vinyl-naphthalenes to produce their respective products 150f and 150g. The chloro-methylene group that has been substituted with styrene is transformed into the equivalent product 150h. Complex alkenes' late-stage functionalization generated from epiandrosterone could also be accomplished using this catalytic amino-fluorination technique 150i.^50^

**Fig. 29:**
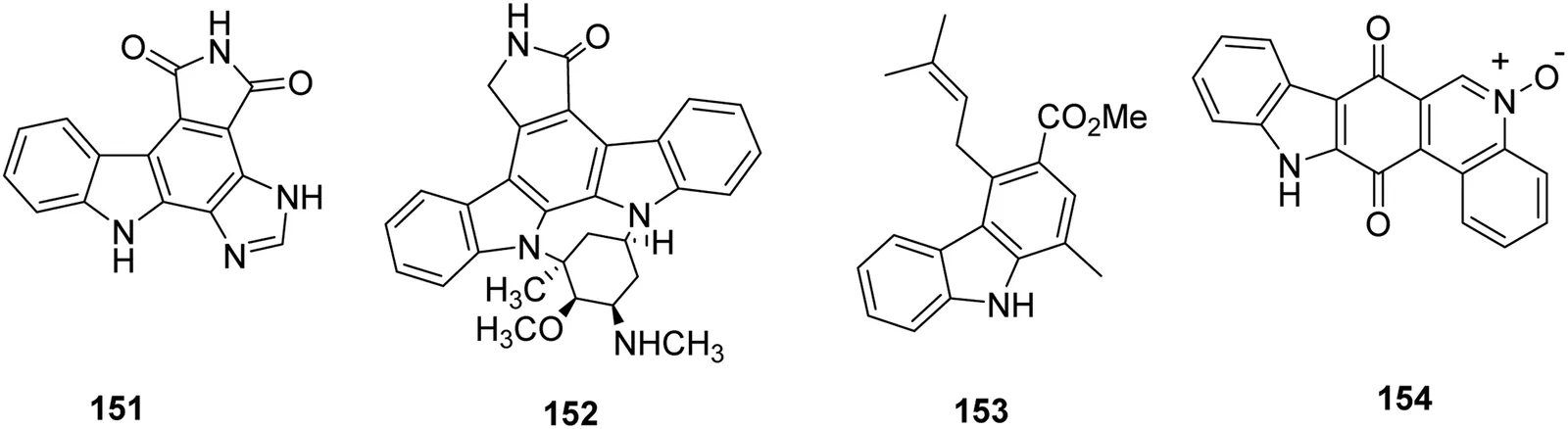
*N*-heterocycles with various constituents that are biologically active.

The *N*-heterocycles are an important component of natural products, medications and structural materials.^272–274^ Widely present in nature and endowed with a variety of biological properties are polycyclic *N*-heterocycles.^275–277^ The representative drugs are the polycyclic *N*-heterocycles quinone-fused calothrixin A 154 (anti-poliferative and effective antimalarial),^278^ staurosporine 152 (kinase inhibitor),^279^ clausamine D 153 (Epstein–Barr virus (EBV) inhibitor)^280^ and the indole-fused carbazole granulatimide 151 (checkpoint kinase 1 inhibitor) (Fig. 29).^281^ The most suitable approach to yield polycyclic *N*-heterocycle compounds is to use Co^+2^ to catalyse the cyclo-amination reaction of indolyl-quinones with various (hetero)aromatic amines.

**Scheme 34:**
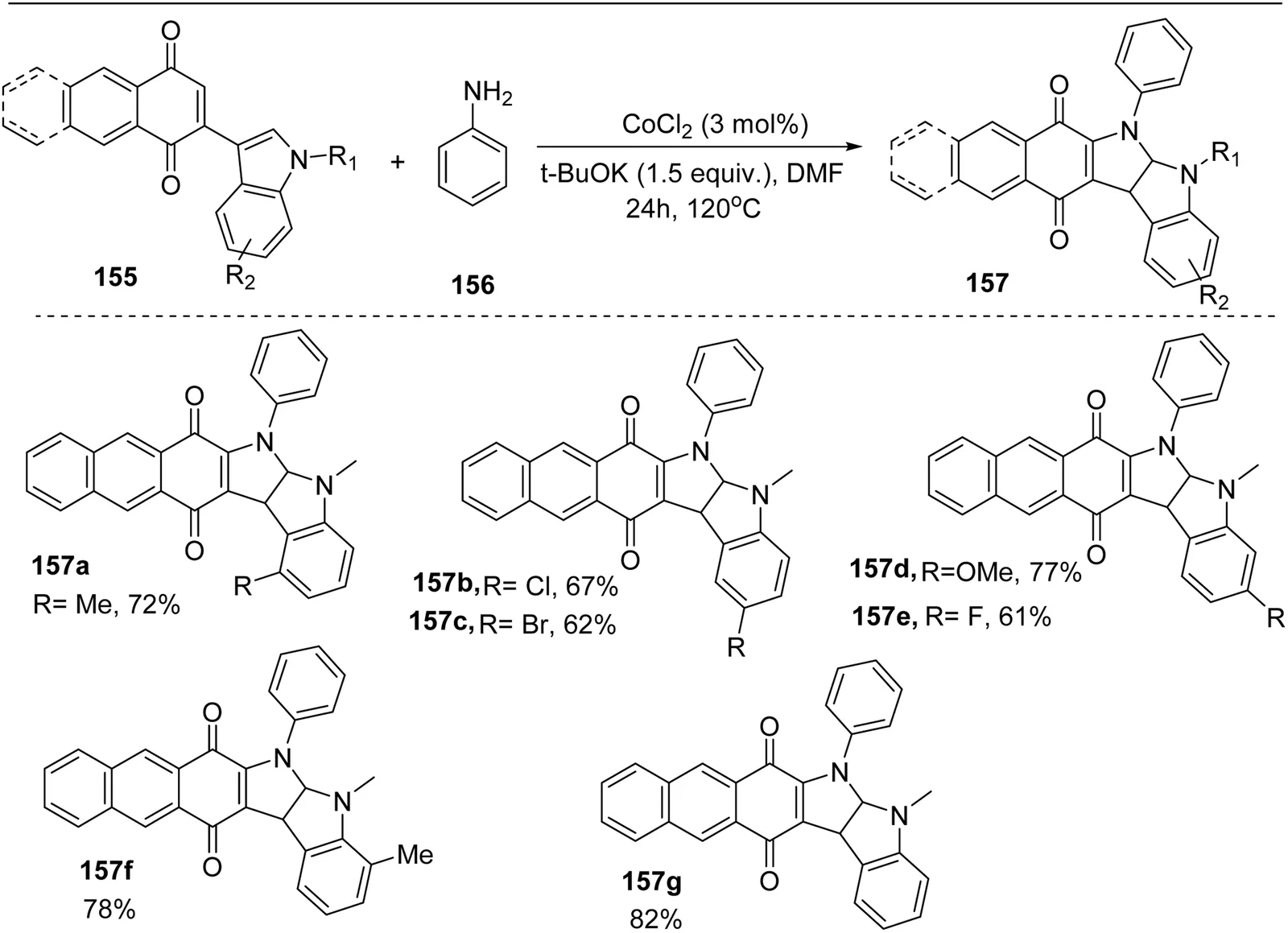
Regarding the synthesis polycyclic *N*-heterocycles, DMF = dimethylformamide.

Dong *et al.*, evaluated a number of substituted indolyl-naphthoquinones in an optimal reaction condition (Scheme 34). The desired polycyclic *N*-heterocycle products 157a and 157d can be obtained from the reaction in a range of indole moieties carrying substituents that either donate electrons or take them away. Additionally, the reaction exhibits good compatibility with many functional groups, including –F(157e), –Cl(157b) and –Br(157c). Due to their frequent usage in later modifications, the tolerance to the halogen atoms was noteworthy. Additionally, the position of the various substituents on the indole moiety had no discernible effect regarding the yield of the reaction and substitutions at the C4-(157a), C5-(157c), C6-(157d) or C7-157f positions were all permitted to react. The 1,4-anthraquinone-substituted indole also underwent these reaction conditions to yield the similar product 157g in 82% yield.^282^

The reactivity of the catalysts is the main determinant of the different cobalt-catalysed processes for the production of biologically active compounds. The reactivity of the cobalt catalysts is dependent on a number of factors, including its capacity to promote hydrogen overflow, crystal phase, reaction circumstances, Co-particle size and dispersion. For example, within the cobalt optimal particle size range of 6–8 nm catalyst activity and product selectivity is increased. Cobalt catalysts have many benefits over other precious metals but they also have some drawbacks. Some of the limitations of cobalt catalysis include its poor surface contact and potential for gasification during the reaction. The surface oxidation of cobalt catalysts is a common occurrence that diminishes their activity. Furthermore, the surface carbon coupling mechanism is often followed by cobalt catalysts, which leads to the production of surface carbon clumps and catalyst deactivation. Cobalt catalysts are challenging to use in reactions that require high temperatures and pressures because cobalt catalysts work well under mild reaction conditions.

### 2.10 Amidation reactions

**Fig. 30:**
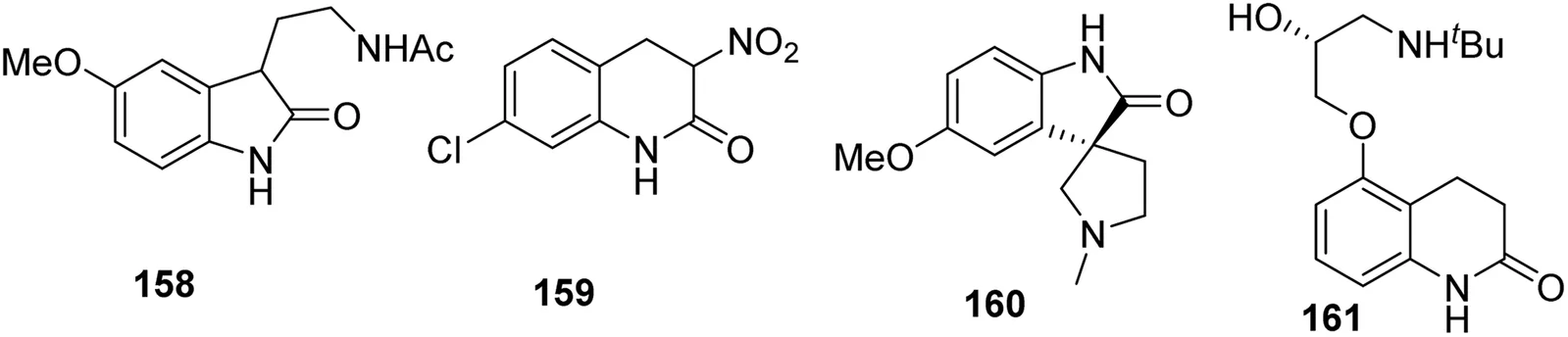
For various compounds that are biologically active.

The Benzofused lactams particularly five-membered indolin-2-one and six-membered dihydroquinolin-2-one, are found as significant heterocyclic frameworks in many bioactive substances and natural products.^283^ They have significant synthetic importance due to their potent biological activities, which include NMDA (*N*-methyl-d-aspartate) antagonist 159 and anticancer, antiviral, antibacterial, analgesic 160 and anti-hypertensive effects^284–288^ in Fig. 30. They were also utilized as fundamental structural building blocks in the formation of extremely complex compounds.^289^ Indolin-2-one and dihydroquinolin-2-one derivatives are made using a variety of conventional ways but the most effective and simple method is the first intramolecular C–H amidation catalysed by Cp*Co(CO)I_2_.

**Scheme 35:**
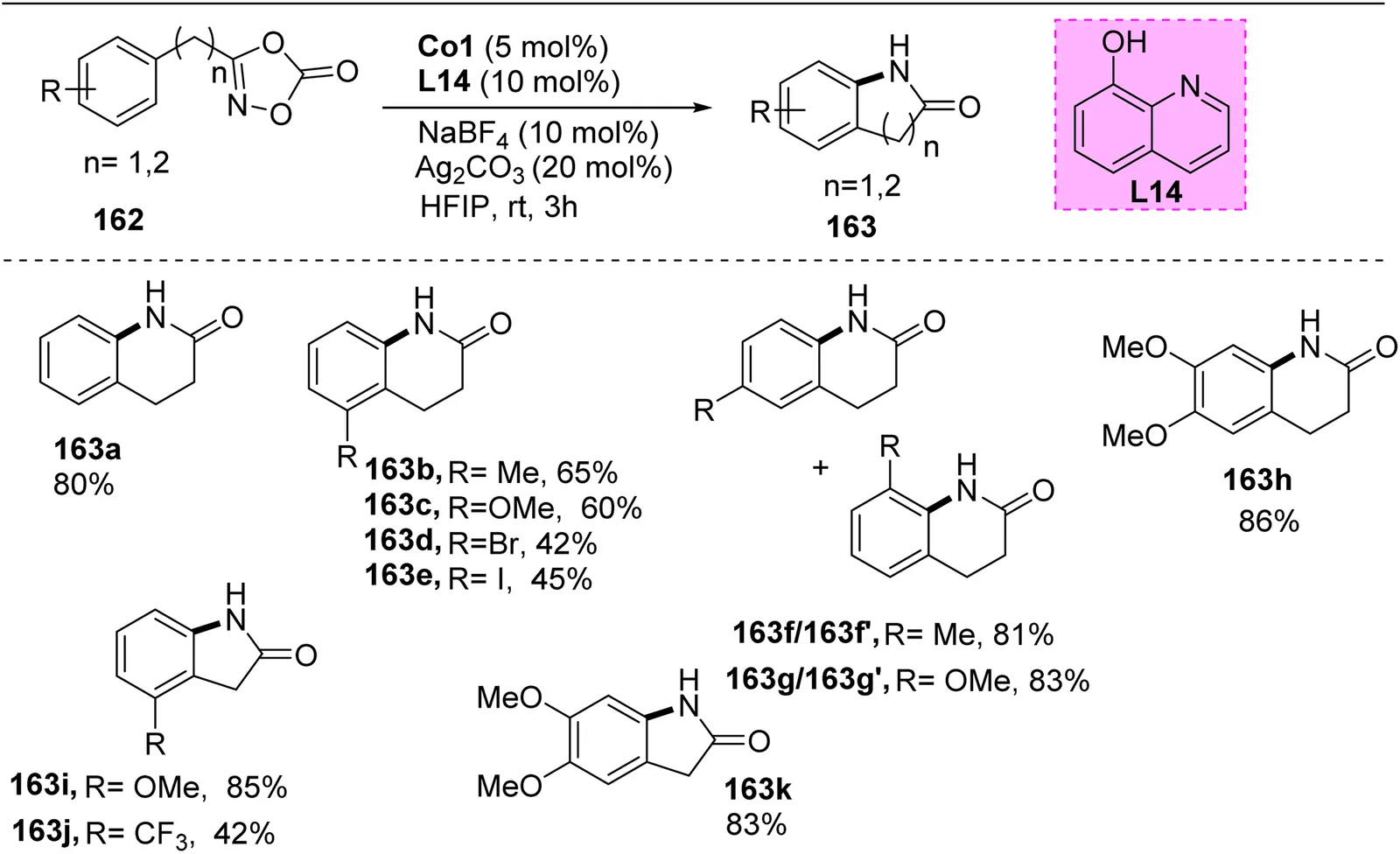
For the synthesis of benzo-fused lactam.

Tian *et al.*, investigated the scope of the intramolecular C–H amidation employing a range of 3-phenethyl-1,4,2-dioxazol-5-ones and 3-benzyl-1,4,2-dioxazol-5-ones 162 under optimum reaction conditions (Scheme 35). The target product 163a was produced with an 80% yield by 3-phenyl-1,4,2-dioxazol-5-one through intramolecular C–H amidation for the synthesis of six-membered dihydroquinolin-2-one derivatives. While electron-withdrawing substituents produce their corresponding products 163d and 163e electron-donating substituents produce the desirable products 163b and 163c in 65% and 60% yield, respectively. The desired isomeric products 163f/163f′ and 163g/163g′ were produced in a mixture with yields of 81% and 83%, respectively from *m*-substituted substrates. The 3,4-dimethoxy-containing substrate works well as a reactant for the C–H amidation, yielding the corresponding product 163h in 86% yield. The formation of five membered indolin-2-one derivatives was the focus of Tian and his co-workers next investigation. The desired products 163i and 163j are produced when *o*-substituted electron-donating (OMe) and electron-deficient (CF_3_) groups are present on a benzyl substrate. The intended product 163k was produced by the substrate containing 3,4-dimethoxy in an 83% yield.^290^

**Fig. 31:**
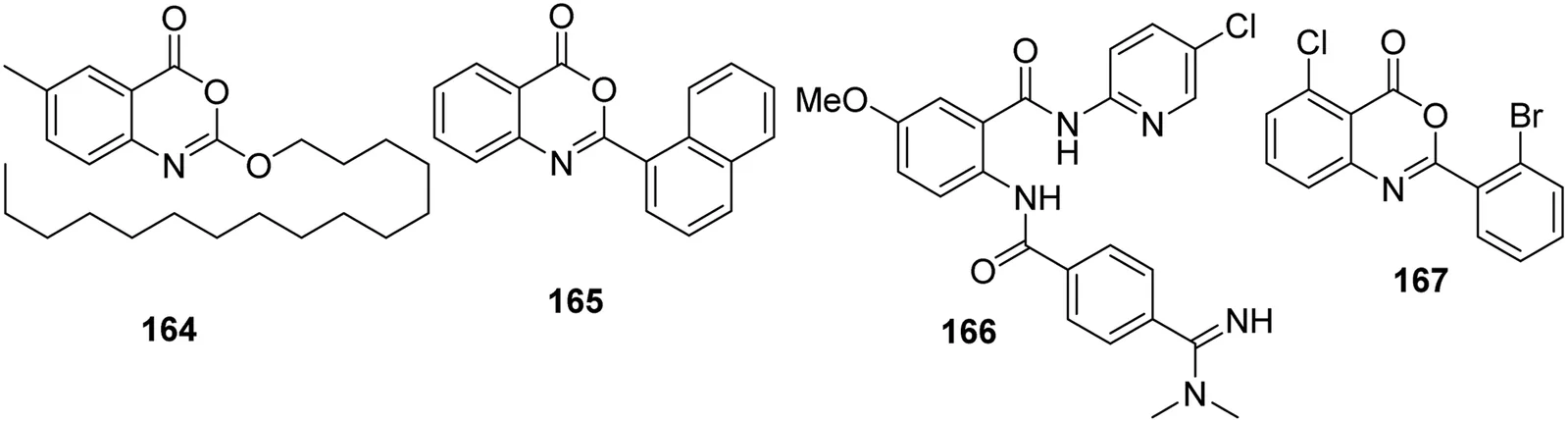
Skeletons of some bioactive 2-formylaniline.

Because many pharmacological drugs, natural compounds and synthetic intermediates contain nitrogen molecule having C–N bond formation, which is crucial in synthetic chemistry.^291–294^ There are many ways to generate C–N bonds but converting an inactive C–H bond to the matching C–N bond has shown to be the most effective approach with the least amount of atom and step waste. The abundant skeletons of biologically active compounds and organic materials can be built using *o*-amino benzaldehyde and its derivatives (Fig. 31).^295,296^

**Scheme 36:**
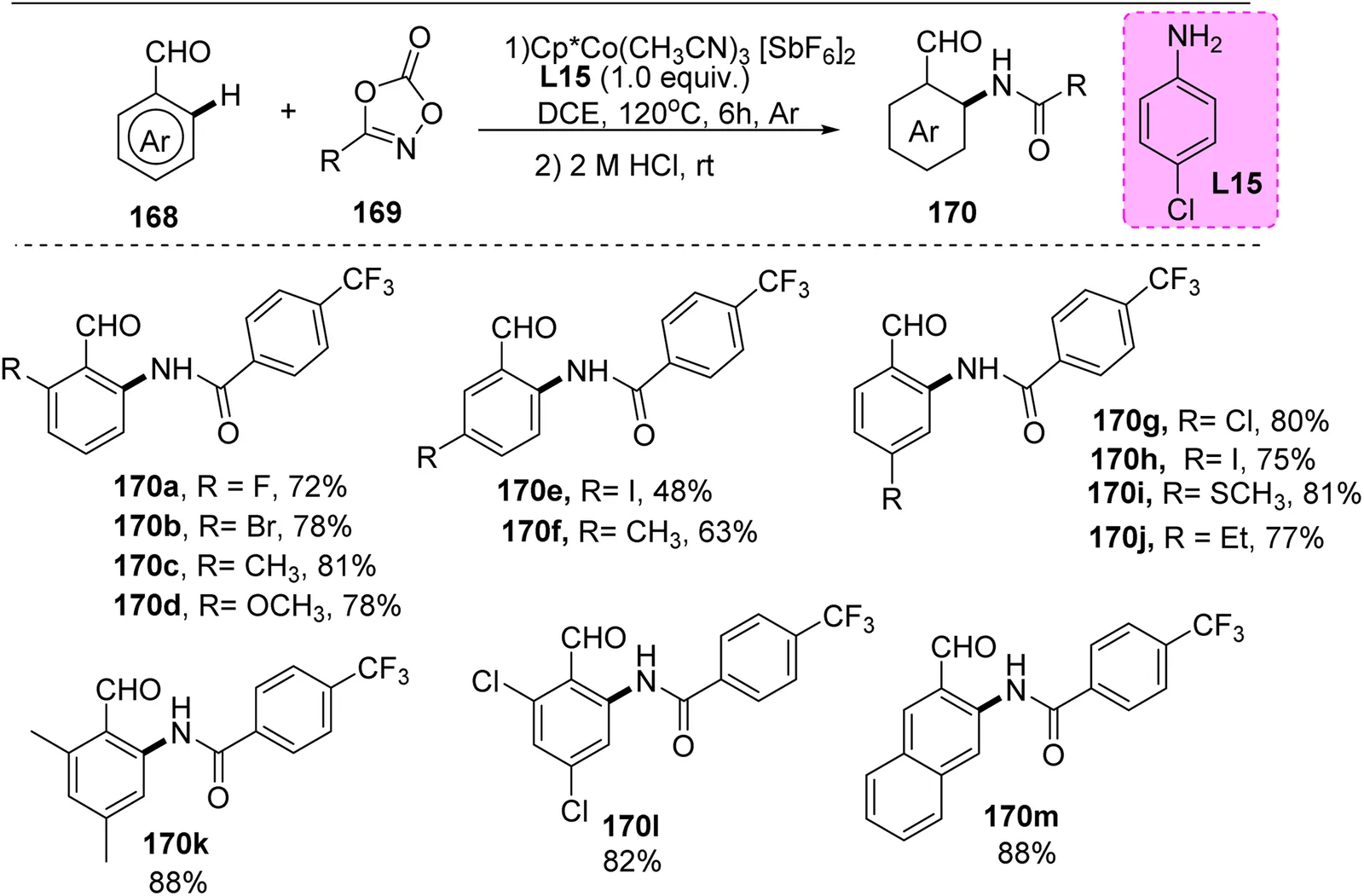
Benzaldehyde amidation with dixazolones.

Huang *et al.*, have studied the scope of benzaldehyde 168 with di-oxazolone 169 under optimal reaction conditions. Their Co-catalysed system tolerated a wide range of benzaldehydes and produced *ortho*-amidated benzaldehydes in good to excellent yields 170a, 170b, 170c and 170d (Scheme 36). Alkyl, –OMe and other electron-donating groups on benzaldehydes were found to be well-tolerated and to give good to excellent yields of the amidation products. The powerful electron-withdrawing benzaldehydes (CN and NO_2_*etc.*) significantly lessen the reactivity of C–H amidation. Only the regioisomers 170e and 170f were produced, less hindered *ortho*-C–H was activated and *meta*-substituted benzaldehydes showed significant regioselectivity. The mono-amidated products 170g, 170h, 170i and 170j were produced and the method exhibits strong mono-selectivity. Benzaldehydes with several substituents also produced the desired C–H amidation products 170k, 170l and 170m.^296^

**Fig. 32:**
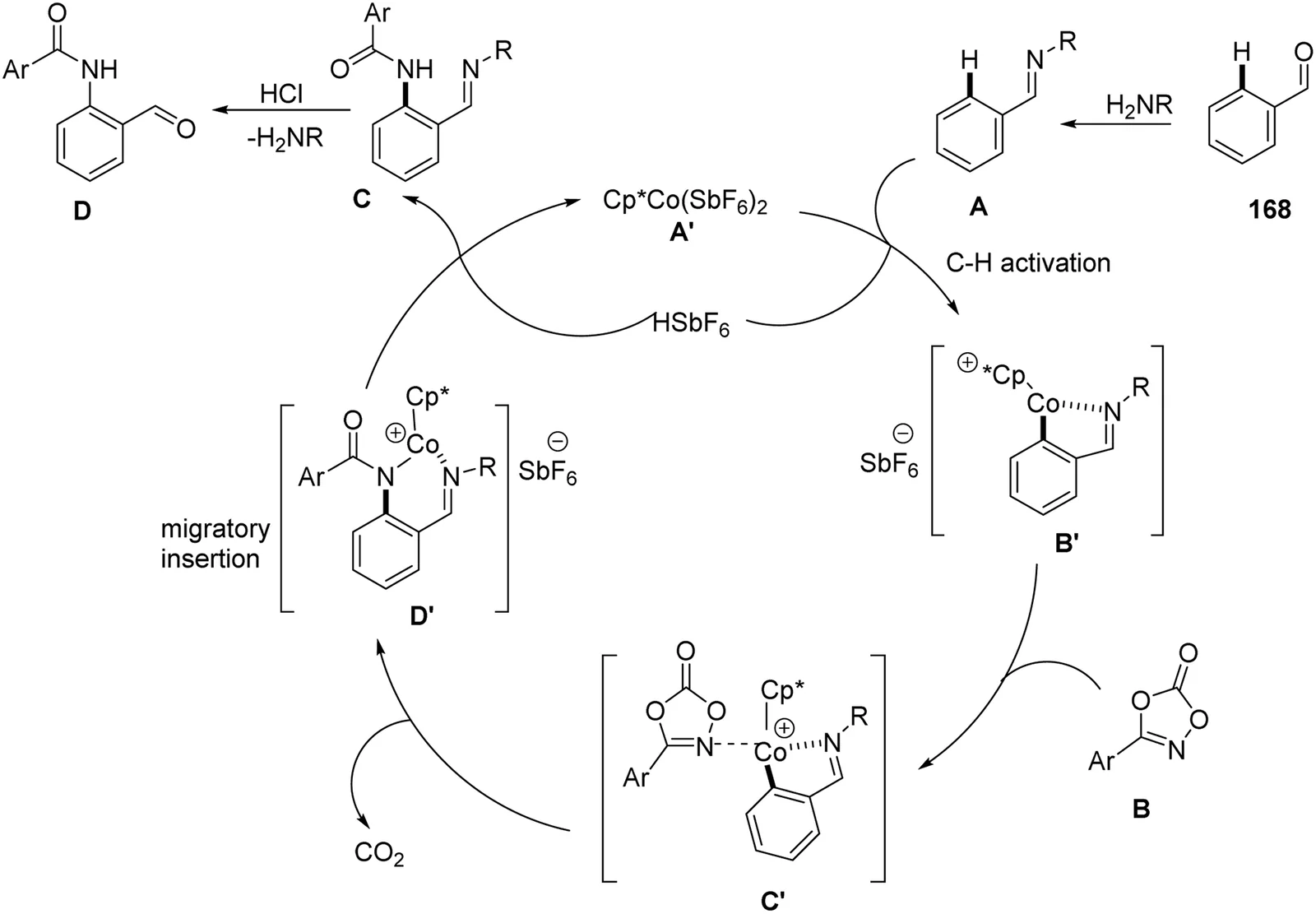
For possible reaction mechanism.

There has been proposed a possible reaction mechanism (Fig. 32). Imine A is initially produced by condensation between 168 and arylamine. The intramolecular C–H concerted metalation deprotonation (CMD), which is facilitated by acetate, then occurs when the Co catalyst and imine combine to form intermediate B′. The intermediate C′ is then formed by the coordination of the di-oxazolones B and it goes through intermolecular migration insertion to produce the intermediate D′ with CO_2_ extrusion. Finally, the Co catalysts A′ and C are regenerated by the protonation of intermediate D′ by newly generated HSbF_6_.^296^

### 2.11 Hydroboration reaction

**Fig. 33:**
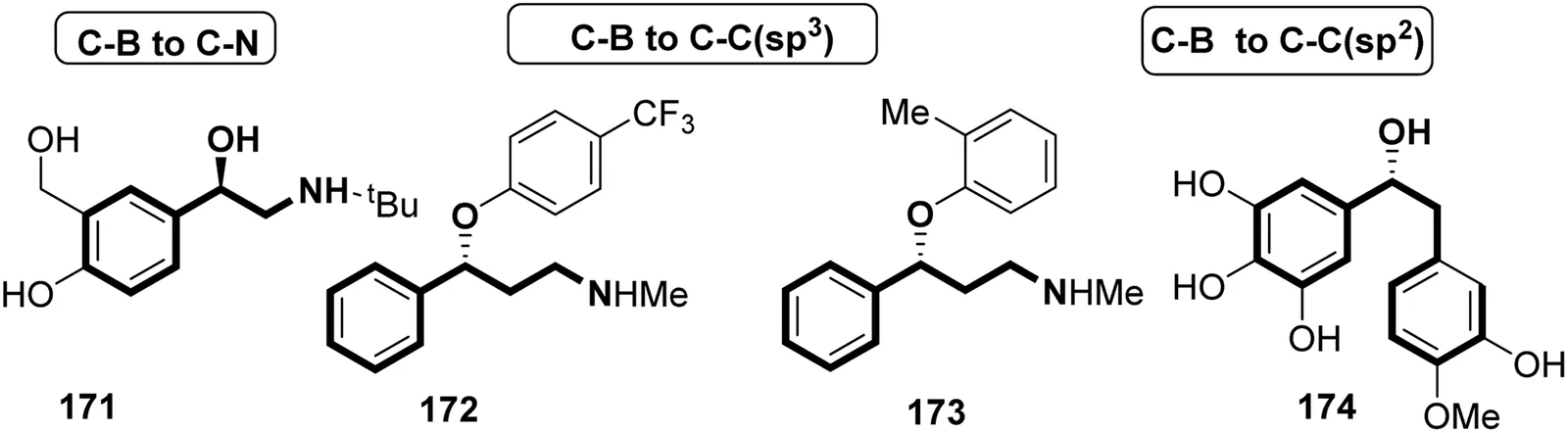
For conversions of β-boryl ethers that are biologically active.

The organoboron compounds are present as important structural components in many biologically active molecules. Additionally, essential in synthetic chemistry and the study of materials are organoboron compounds.^297–301^ Asymmetric synthesis relies heavily on the stereospecific conversion of C–B bonds into C–C or carbon–heteroatom bonds for the formation of complex compounds (Fig. 33).^302–304^ Enantioselective hydroboration of silyl enol ethers produced from ketone was carried out using the most earth plentiful and economically advantageous cobalt catalyst and chiral ligand. This would provide easy, quick access to chiral β-borylethers, which are privileged motifs that are widely present in natural products, medicines and agrochemicals. These compounds can also be transformed easily into amino alcohols and diols.

**Scheme 37:**
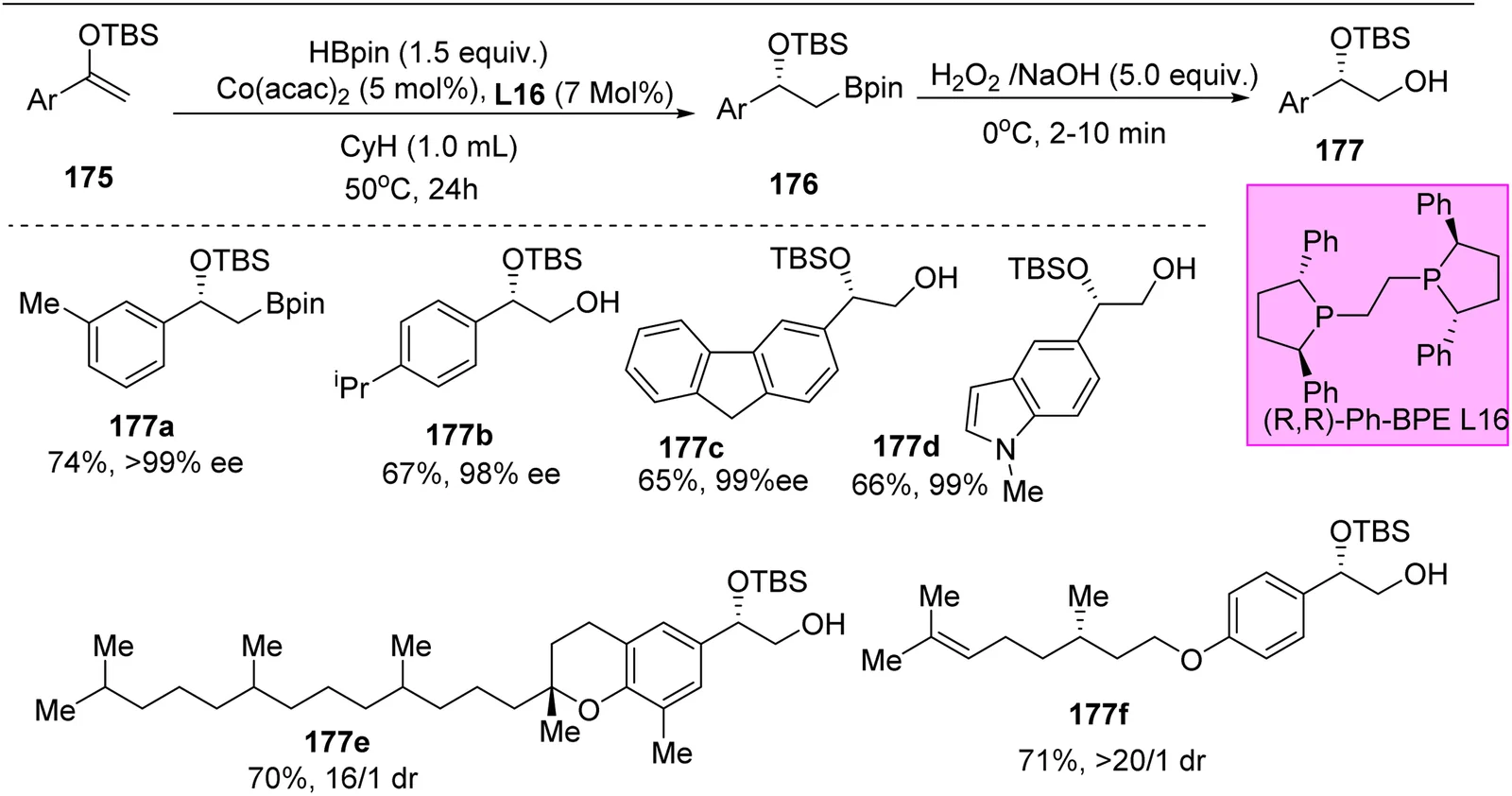
For the hydroboration reaction.

Dong *et al.*, have investigated the range of substrates for Co-catalysed asymmetric hydroboration using silyl enol ethers 175 (Scheme 37). This enantioselective hydroboration procedure involved a variety of Ar-substituted silyl enol ethers that produced the desired products in good yields and with excellent enantioselectivities. For the purpose of determining *ee* and in certain cases making separation easier, the boronic esters 176 were oxidized to the corresponding diols 177. All of the substrates, irrespective of the substituent's position on the –Ph group underwent a smooth reaction to produce the appropriate β-hydroxy boronic esters or diols in good yields and with excellent enantioselectivities 177a and 177b. A variety of functional groups could be used with the mild reaction conditions, including electron-donating groups and electron-withdrawing groups, such as Ph, CH_2_OBn, morpholine, OMe, OBn, *tert*-butyldimethylsilyl ether, CF_3_, F, acetal, Bpin and TMS, and they all produced the corresponding products in 97–99% *ee*. Other aromatic substituents, besides phenyl groups, could also be effectively introduced into the products 177c and 177d including fluorene and indole. The approach was applied to a variety of silyl enol ethers produced from complex molecular structures in order to further illustrate its applicability. They felt happy when they realized that the silyl enol ethers containing citronellol and δ-tocopherol underwent this enantioselective hydroboration, yielding the diol products 177e and 177f in good yields and dia-stereoselectivities.^305^

**Fig. 34:**
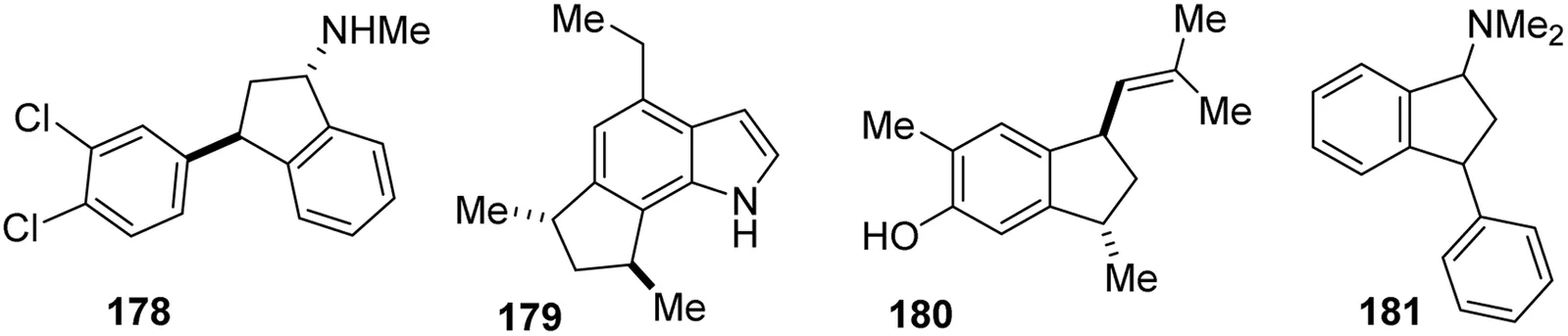
Bioactive molecules and pharmaceutical components that contain indanes.

A number of medications, bioactive substances, and natural products contain the indanyl unit as a significant, recurrent substructure (Fig. 34).^306–310^ The synthesis of indanes involves techniques including direct C–H activation, Michael-type cyclization, ring-enlargement or reduction in size *etc.*^311–316^*Trans*-1,3-disubstituted indanes can be produced effectively using metal-catalysed hydroboration of 3-substituted indenes.

**Scheme 38:**
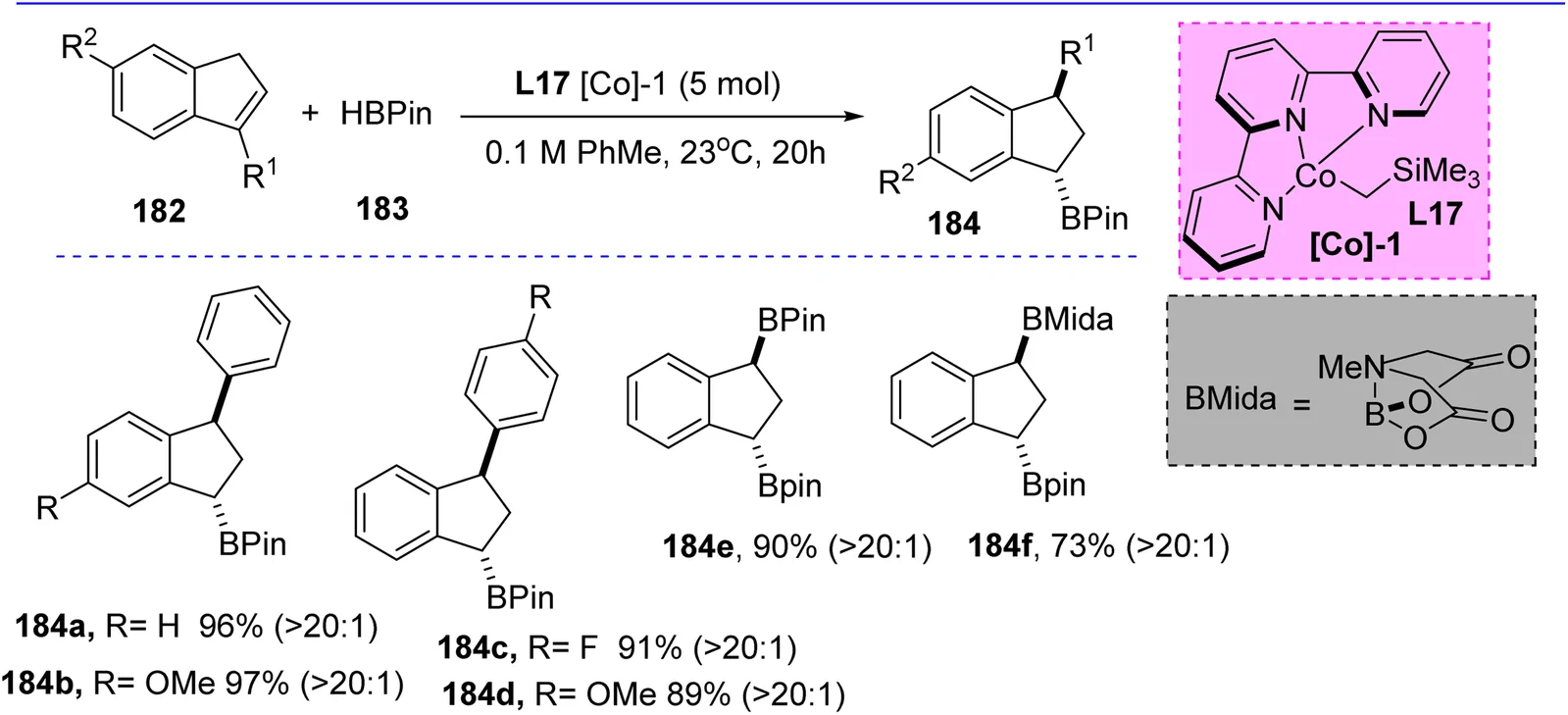
For the synthesis of stereodefined 1,3-difunctionalized indanes.

The results of these studies of Léonard *et al.*, which looked into the range of the isomerization–hydroboration reaction are shown in Scheme 38. Under standard conditions, 1.05 equiv. of HBPin was added to a 0.1 M solution of indene in toluene with 5 mol% [Co]-1. Alkyl-substituted indene isomerization and hydroboration were carried out with good yields and diastereo-selectivity using cobalt as the catalyst. The boron's selective anti-arrangement was further structurally confirmed by X-ray crystallography of the Ph-substituted product 184a. The catalytic approach was likewise compatible with Ar-substituents having electron-withdrawing 184c and electron-donating 184d groups at the 4-position. Additionally, hydroboration was performed on a substrate 184b that had a methoxy group substituted at the indene's 6-position without reducing the isolated yield of the required indenes. Heteroatom-substituted indenes 184e produce a 1 : 1 mixture of diastereomers, probably as a result of the group –Me directing influence acquiring the reaction's basic diastereo-selectivity. Indenes 184f with a boron substitution were also investigated. The 1,3-disubstituted indanyl diboron compounds with boron substituents make intriguing construction elements for the quick assembly of intricate the structures of molecules.^317^

**Fig. 35:**
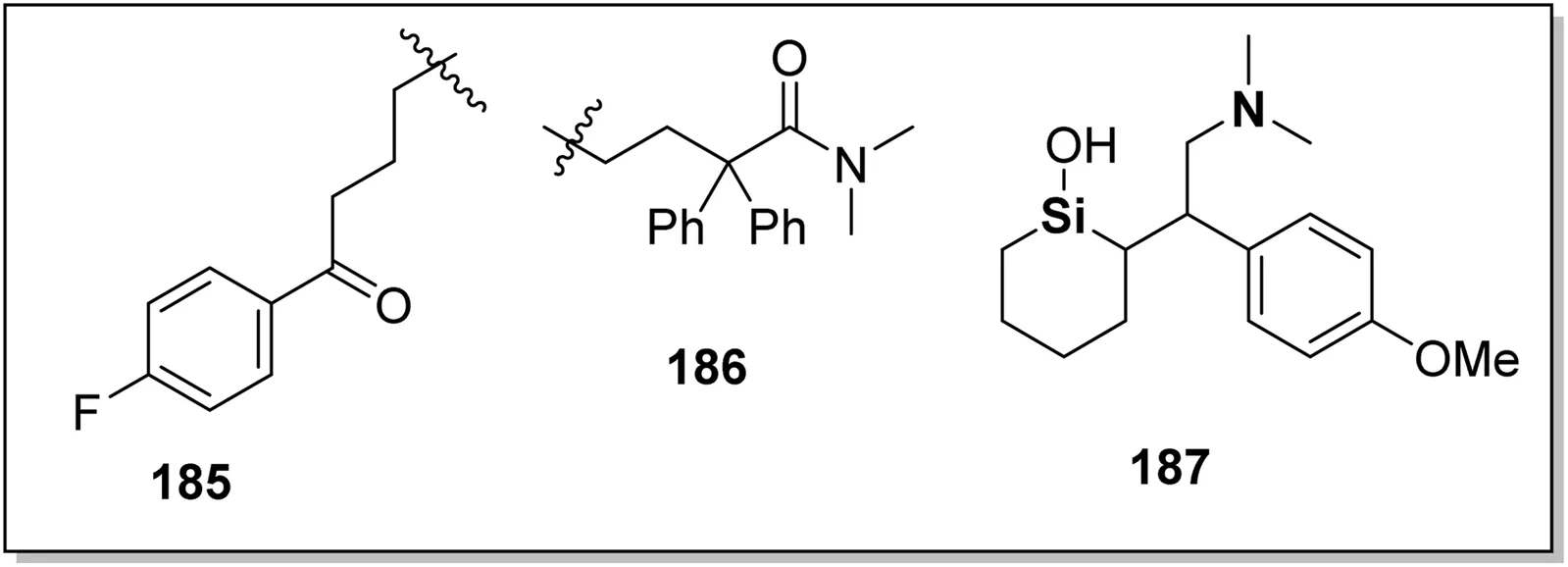
For cyclic and acyclic compounds with potential medicinal uses.

Organosilicon compounds have a significant role in medicinal chemistry due to minor physical and electronic modifications, such as silasubstitution or bioisosteres of carbon or other moieties.^318,319^ Many biologically active substances including sila-haloperidol 185, sila-loperamide 186 and sila-venlafaxine 187, have vicinal amino and silyl (1,2-N, Si) molecules that simultaneously bear amino and silyl groups (Fig. 35).^320–322^ Co-catalysed ligand-controlled catalysis using hydrosilanes and diazo compounds to hydrosilylate and hydro-hydrazidate terminal alkynes in a chemo- and regioselective manner has been reported as the most effective and suitable method for producing vicinal amino and silyl products bearing both an N–H bond and a Si–H bond.

**Scheme 39:**
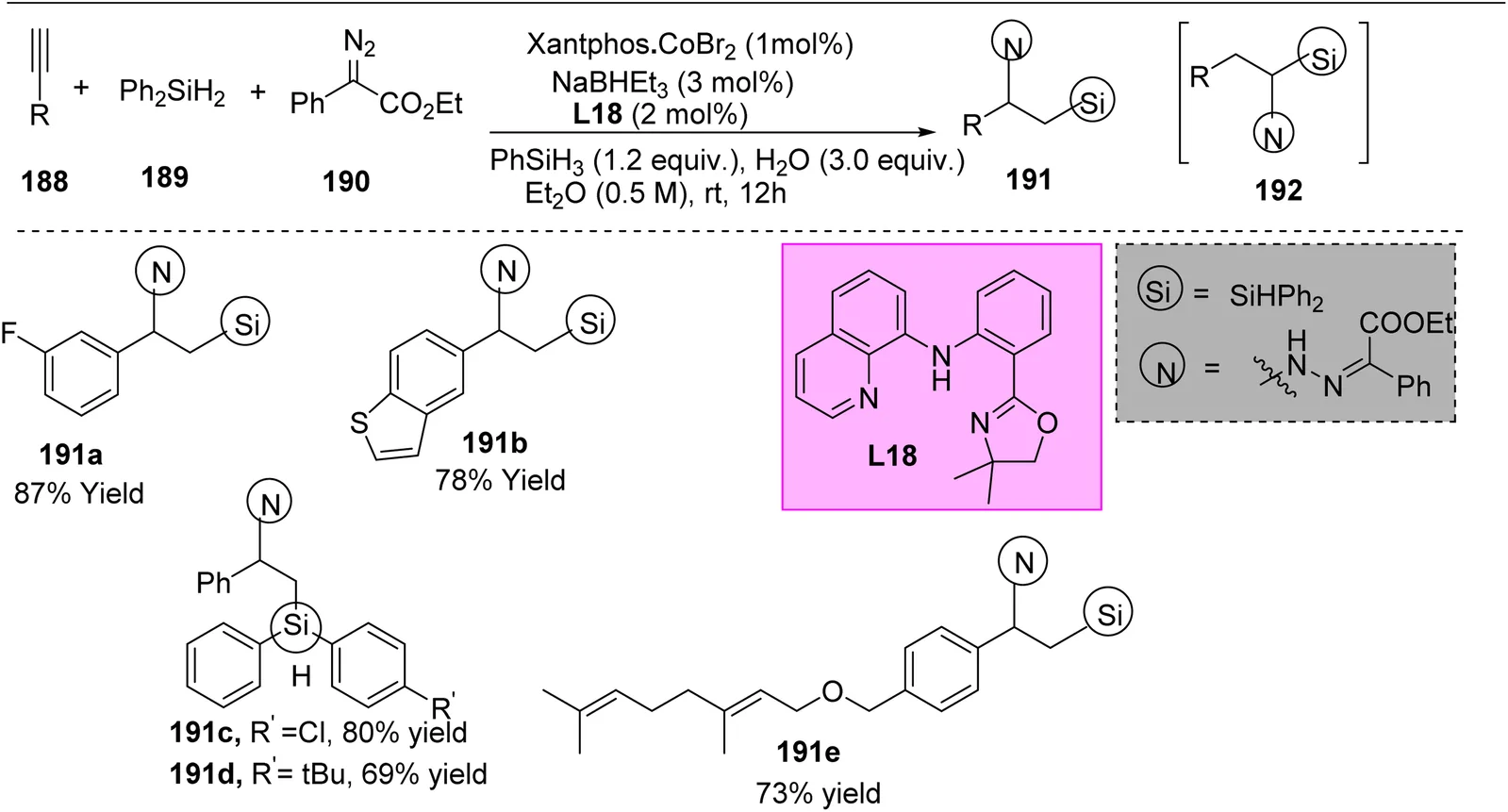
Terminal alkynes undergo hydrosilylation and hydrohydrazidation.

Sun *et al.*, have examined the substrate scope under optimum reaction conditions (Scheme 39). On aryl rings substitution at *ortho*- and *meta*-positions can be easily put up 191a. The benzo-thiophene-containing substrate is also a suitable substrate for this reaction which produces the desired product 191b with a 78% yield. It was difficult to obtain these compounds because it was difficult to observe the alkyl substituted 1-amino-2-silylalkanes during the amino-silylation of alkenes or the ring-opening C(sp^3^)–Si cross-coupling of aziridines. Geraniol and other bioactive skeleton-containing aryl alkynes go through these reaction conditions to produce an analogous product in 191e in 73% yield demonstrating the possibility of late-stage modification of complex compounds. This reaction could involve various hydro-silanes, *p*-chloride and *t*-Bu substituted diphenyl silanes, and phenylsilanes to efficiently produce the respective products 191c and 191d in yields of 69–80%.^323^

**Fig. 36:**
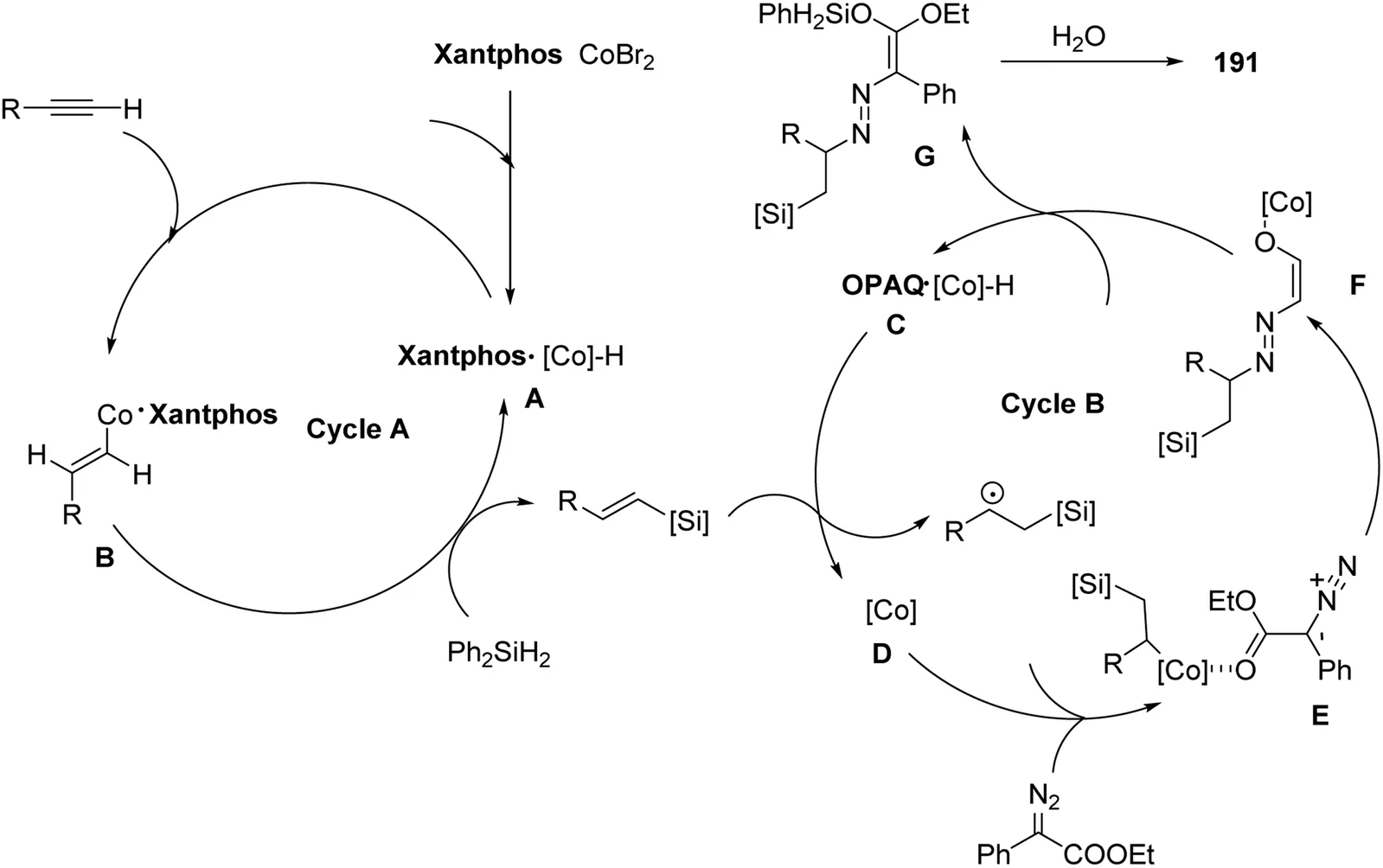
For the proposed reaction mechanism.

In Fig. 36, a potential reaction mechanism has been put forth. NaBHEt_3_ first activated Xantphos CoBr_2_ to produce intermediate A, a Co–H, which would coordinate with the alkyne. The β-vinyl cobalt intermediate B was then produced by a further anti-Markovnikov type alkyne insertion into the cobalt hydride species, which could then undergo σ-bond metathesis with diphenyl-silane to produce vinyl silane. A ligand exchange procedure would be performed on the regenerate intermediate A to provide the OPAQ cobalt hydride species C. The most important step during the ligand transfer mechanism is choosing the proper a ligand that can be interchanged for the one that was previously used. Here, OPAQ, a tridentate anionic *N*-ligand, demonstrated a greater coordination effect with the cobalt ion than did Xantphos, a bidentate neutral P-ligand, ensuring the efficacy of the hydro-hydrazidation of alkenyl silanes. The former metal catalyst, which has already lost the core metal ion, won't have an impact on the subsequent reaction. Through metal hydride hydrogen atom transfer (MHAT), the newly formed intermediate C may produce a radical intermediate and a cobalt intermediate D, which could then interact with one another in the presence of a diazo compound to produce the intermediate E. In order to produce the azo enolate cobalt intermediate F, the cobalt species E might next go through alkyl group migration. This would be followed by σ-bond metathesis with PhSiH_3_ to produce the silyl enol ester intermediate G and regenerate the cobalt hydride intermediate C. The end product would be produced by sequential hydrolysis and isomerization of intermediate G in the presence of H_2_O.^323^

### 2.12 Coupling reaction

**Fig. 37:**
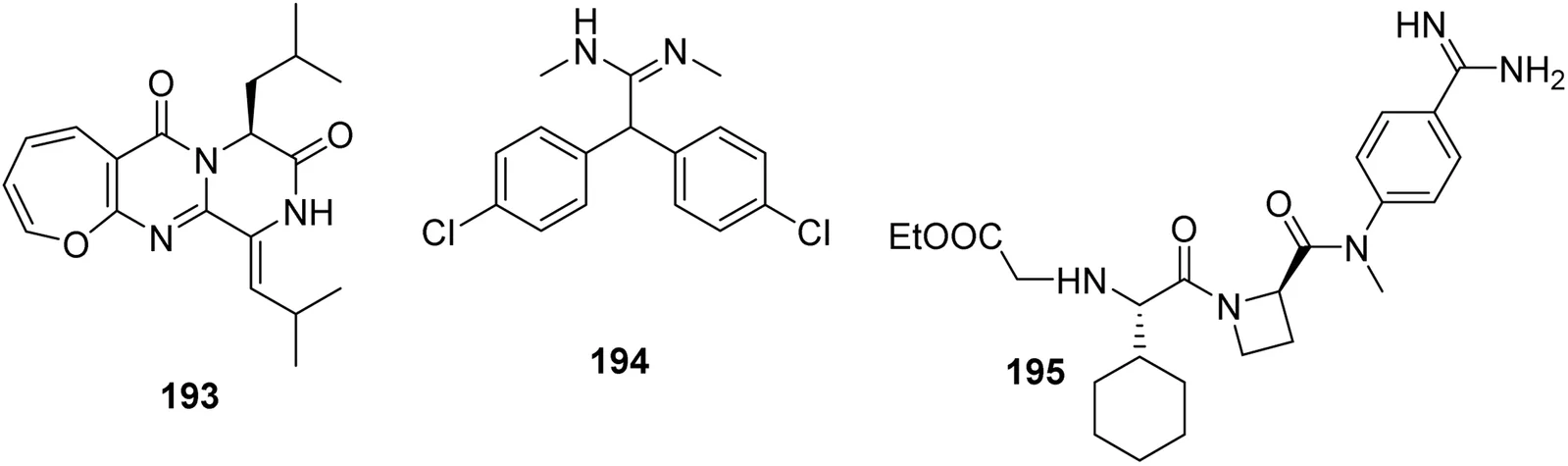
Biologically active heterocycles.

Amidines are abundantly present in products that are organic and biologically active compounds, and they have great importance for this reason.^324–327^ As non-nucleophile bases,^328,329^ organometallic ligands complexes^330–332^ and components needed to produce heterocycles, amidines are employed in the study of organic chemistry.^333–339^ As a result, organic and medical chemists have developed a keen interest in amidines due to their significance and utility (Fig. 37).

**Scheme 40:**
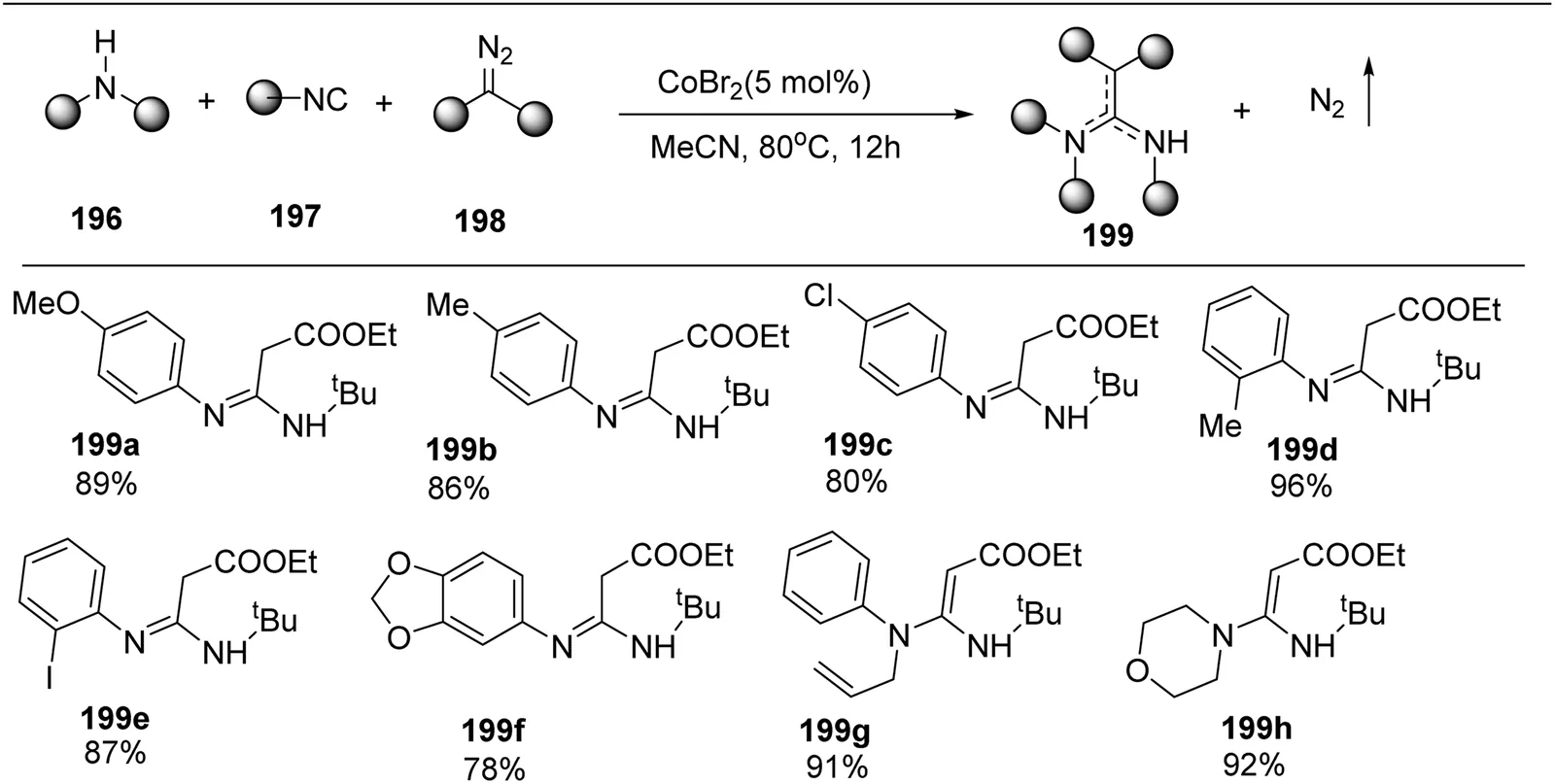
For the synthesis of functionalized amidines, MeCN = methyl cyanide.

Through the coupling reaction of the cobalt–carbene radical, Gu *et al.*, studied the scope of a variety of amines 196 under optimal reaction conditions. The electron-donating groups on the *p*-substituted anilines (OMe, Me) showed good reactivity and produced the desired results 199a and 199b. The related compounds are produced in a modest yield from the electron-withdrawing group-containing *para*-substituted anilines. The desired product 199c was produced by the anilines substituted with *para* halogen (Cl), which were tolerable. The *ortho*-methyl and *ortho*-iodo substituted anilines exhibited good reactivity and yielded the desired product 199d and 199e with excellent yields. In a reaction involving coupling with 3,4-(methylenedioxy) anilines, the amidine product 199f was produced with a 78% yield. Under these reactional conditions, secondary amines like *N*-allylaniline and morpholine produced the corresponding products 199g and 199h in a high yield. Biologically active molecules and medicinal substances both frequently comprise heterocycles containing indoles (Scheme 40).^340^

**Fig. 38:**
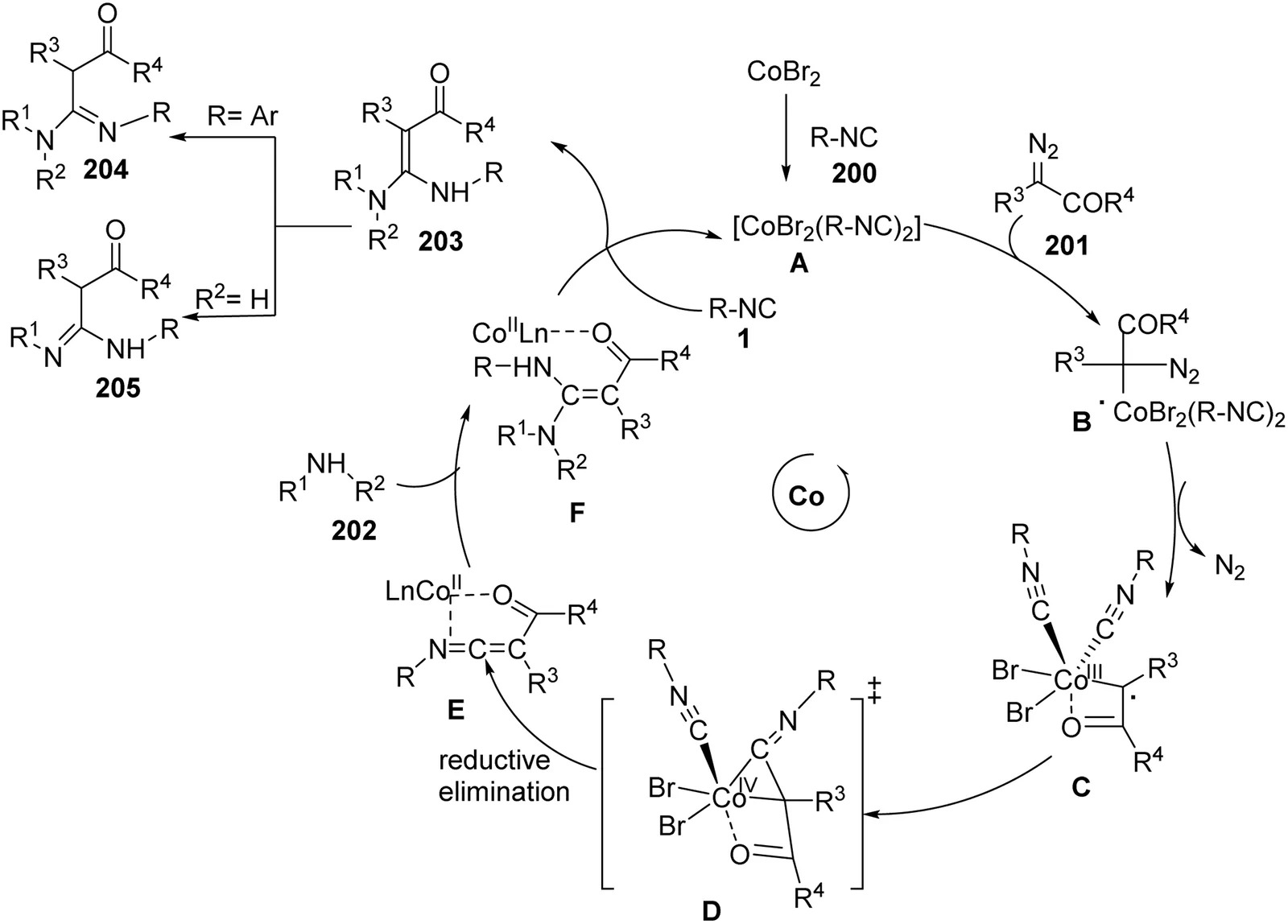
Mechanism for the synthesis of functionalized amidines.

In Fig. 38, an appropriate reaction mechanism has been presented. The isocyanide 200 first reacts with the CoBr_2_ to produce intermediate A. The cobalt species B is then produced by reacting diazo 201 with intermediate A. When N_2_ is released the Co–carbene radical species C is produced. Next, intermediate D was produced through the coordinated isocyanide ligand and carbene radical coupling reaction. The Co(ii)-ketenimine intermediate E is generated by the following elimination of intermediate D through reduction. Additionally, the intermediate F is produced when the amine 202 interacts with intermediate E to produce intermediate F. Following a ligand substitution with isocyanides, intermediate F regenerates into the catalyst cobalt and the targeted product 203, 204, 205.^340^

**Fig. 39:**
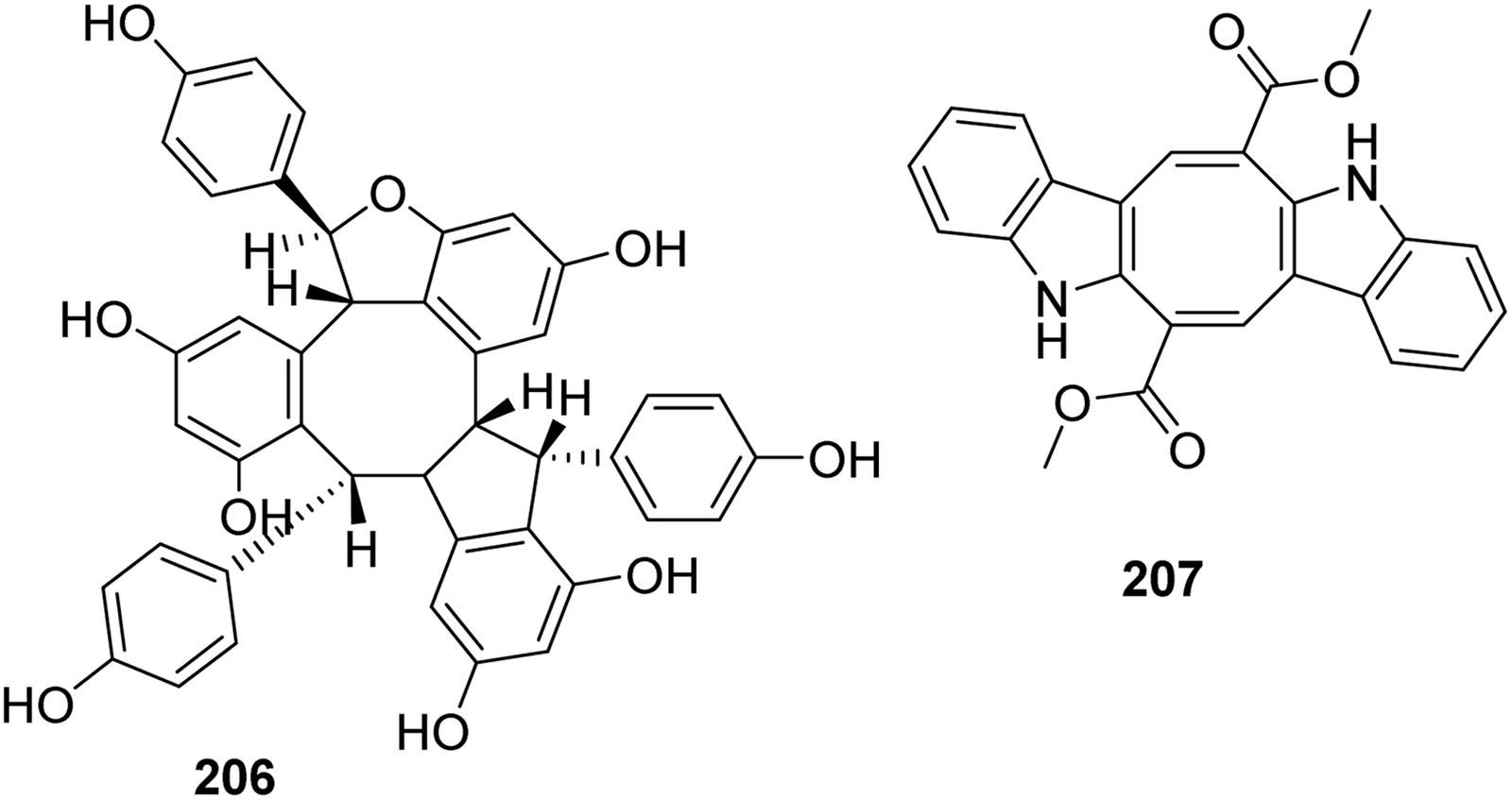
For the bio-active natural products.

The presence of 8-membered rings in a wide range of bioactive compounds,^341^ perfumes,^342^ fuels^343^ and catalysts makes them significant.^344,345^ There are several ways of building 8-membered rings including ring closure metathesis,^346^ carbene–carbene cyclizations,^347^ metal-catalysed [4 + 4]^348,349^ or [6 + 2] cycloaddition^350,351^ and oxabicyclic metal-promoted ring opening processes^352^ have been developed. These methods produce a lot of by products, hence an effective and primarily suitable method using a cheap metal catalyst has been established for the synthesis of 8-membered rings (Fig. 39).

**Scheme 41:**
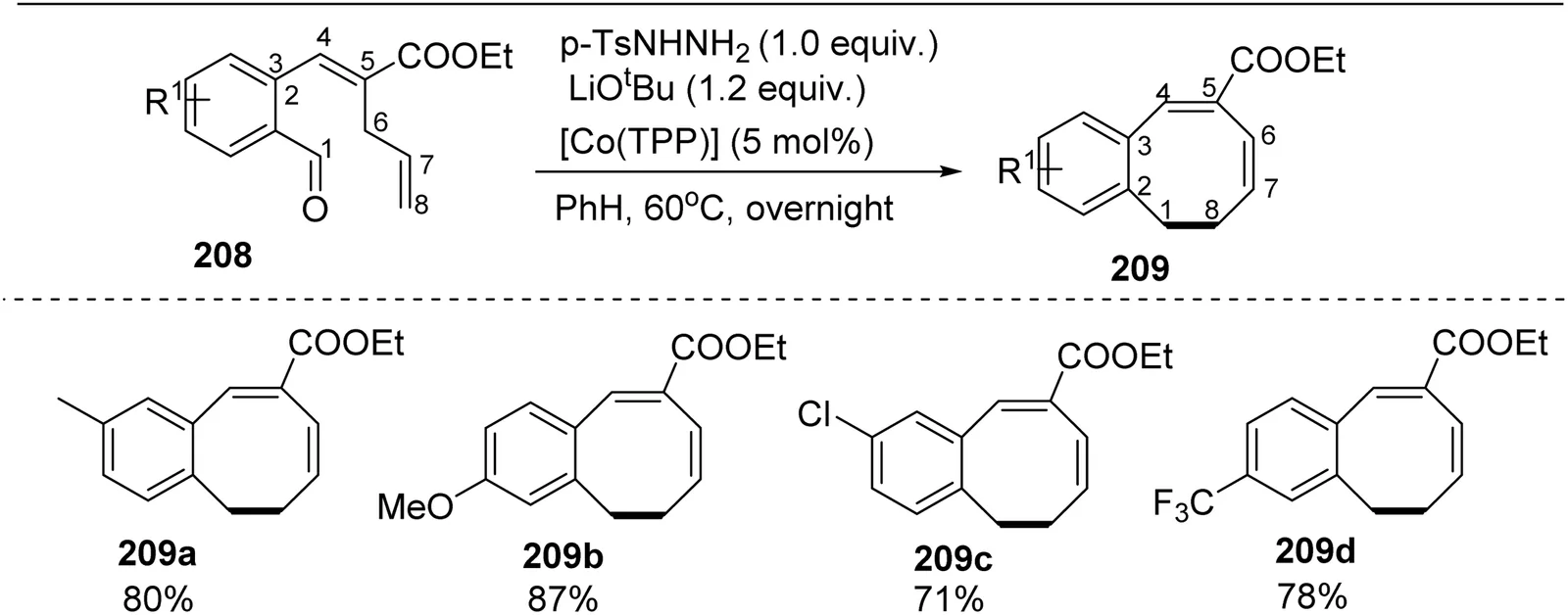
For the synthesis of compounds with eight members.

Zhou *et al.*, investigated the range of substrates available for the synthesis of mono-benzo cyclo-octadiene rings 209 with eight members (Scheme 41). The efficacy of the reaction is first examined in relation to various aromatic ring substituents. Although different yields of products are produced, the substrate when heated in the presence of base, it is not very stable because it contains electron withdrawing groups. It demonstrates that at the usual reaction conditions (60 °C, 5 mol% catalyst), lesser yields of the relevant product are achieved. In order to prevent base-mediated substrate degradation and produce the corresponding product in good yield, therefore lowered the reaction temperature while substantially increasing the catalyst loading. A required product may be acquired with favourable yields under these adjusted reaction conditions 209c and 209d. The desired product is typically produced in good yields from substrates with groups on the aromatic ring that either donate or withdraw electrons. while substrates with electron-donating groups produced the mono-benzo cyclo-octadiene's in marginally greater yields 209a and 209b. Therefore, it is evident that the phenyl ring substitution pattern has no effect on reaction yield and that heat is not always necessary to transform *N*-tosyl hydrazones into the necessary diazo compounds.^353^

**Fig. 40:**
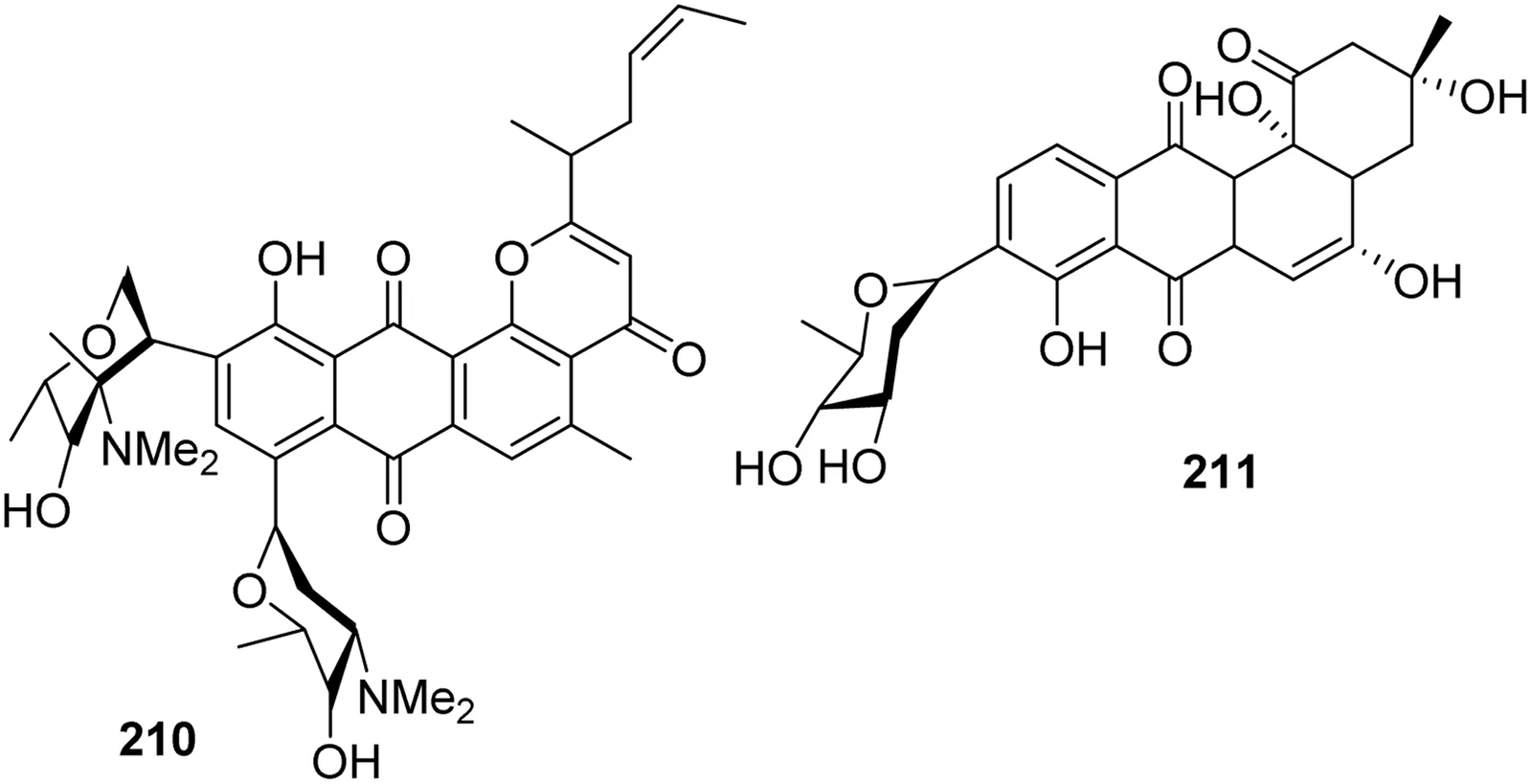
Different biologically active compounds with a glycoside moiety.

Natural products contain *C*-glycosides, which have a variety of biological activities.^354^ In comparison to *O*- or *N*-glycosides, *C*-glycosides are more stable throughout both enzymatic and chemical hydrolysis.^355–359^ Numerous naturally occurring substances of medicinal value including aquayamycin 211 and saptomycin B 210 (Fig. 40) include the 2-d-*C*-glycosides structure in the majority of natural compounds.^354^

For the greatest part in natural compounds, the 2-d-*C*-glycosides structure is predominant.^360^ Because there are no participating groups at the C2 position, it is more difficult to directly synthesize moieties having beta-configuration. Therefore, the radical method has exceptional stereoselectivity and is used to synthesis the majority of *C*-glycosides.

**Scheme 42:**
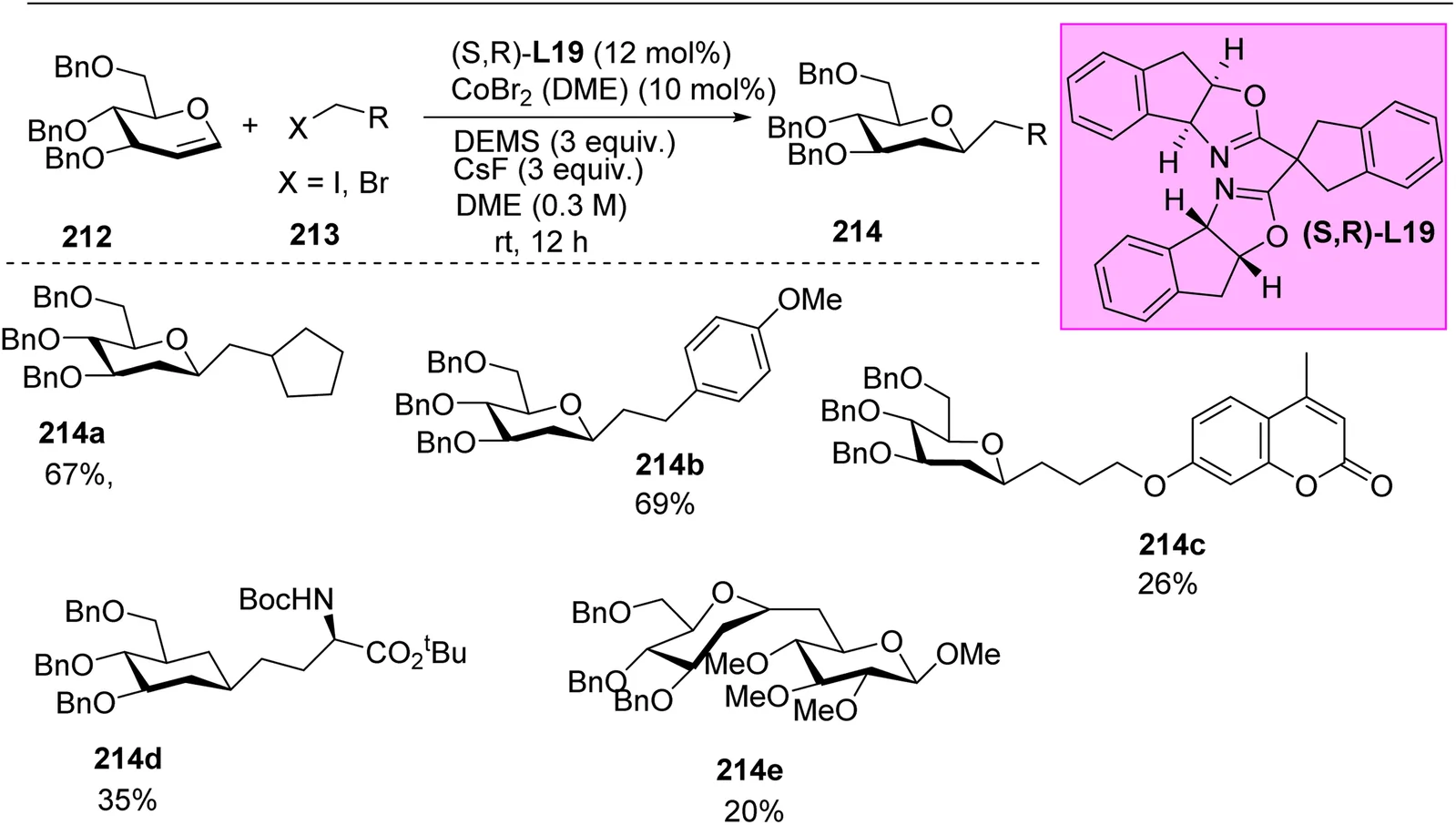
For the purpose of developing various glycoside derivatives, DEMS = diethoxymethylsilane.

Bartoszyk *et al.*, examined the range of substrates for alkyl halides 213 under optimal reaction conditions. Alkyl halides with cycloalkyl groups 214a and aryl groups 214b reacted without difficulty and the good dia-stereoselectivity was maintained (only in the β-configuration) (Scheme 42). Drug compounds and natural products can also be modified in the late stages using this technique. A variety of structurally intricate bioactive alkyl fragment-containing β-2-deoxy-*C*-glycosides were effectively synthesized. For instance, target products with potential bioactivity were easily created from alkyl iodides generated from natural products like cumarin 214c and homoserine 214d. This method was also successful in generating glycosidic C–C bonds with the β-configuration during the synthesis of disaccharide 214e. Many naturally occurring substances with complicated and various structures contain the β-configured disaccharide moiety including the anthelmintic hikizimycin, the neurotoxin maitotoxin and other synthetic substances like dodecodiulose.^361^

**Fig. 41:**
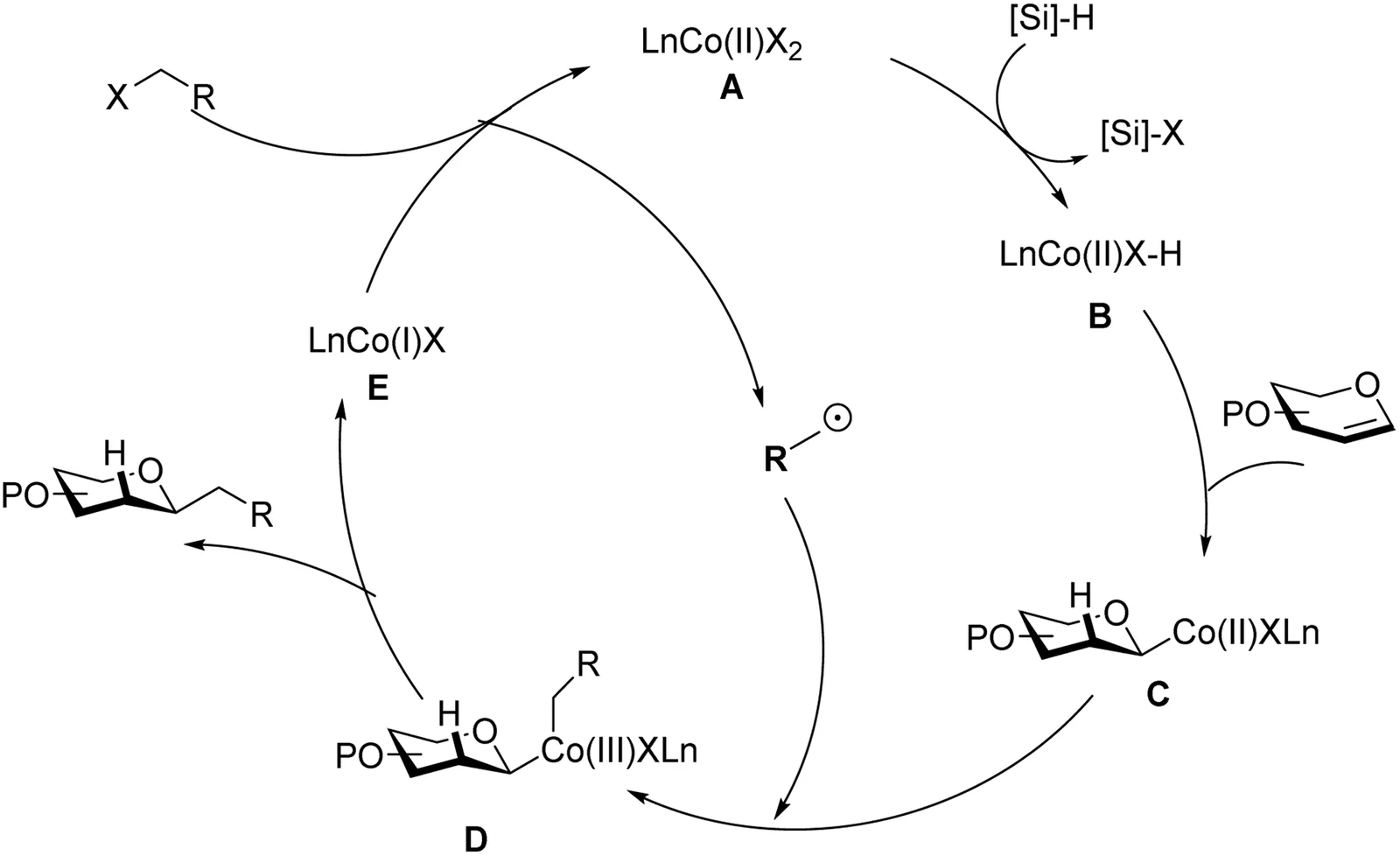
Plausible mechanism for the glycosyl hydroalkylation.

Fig. 41 suggests a probable mechanism for the glycosyl hydroalkylation. With the help of silane, the initial catalyst LnCo^II^X_2_A produces the metal hydride LnCo^II^X-H B. The *syn*-addition adduct alkyl cobalt species C is produced when B inserts into the C–C double bond. This adduct has the ability to rapidly bind the alkyl radical, forming the Co^+3^ species D. The target product (β-configuration) and Co^+1^ species E are then produced by reductive elimination from D. This Co^+1^ species E then regenerates the catalyst A which was employed initially and generates an alkyl radical by reacting with RX.^361^

**Scheme 43:**
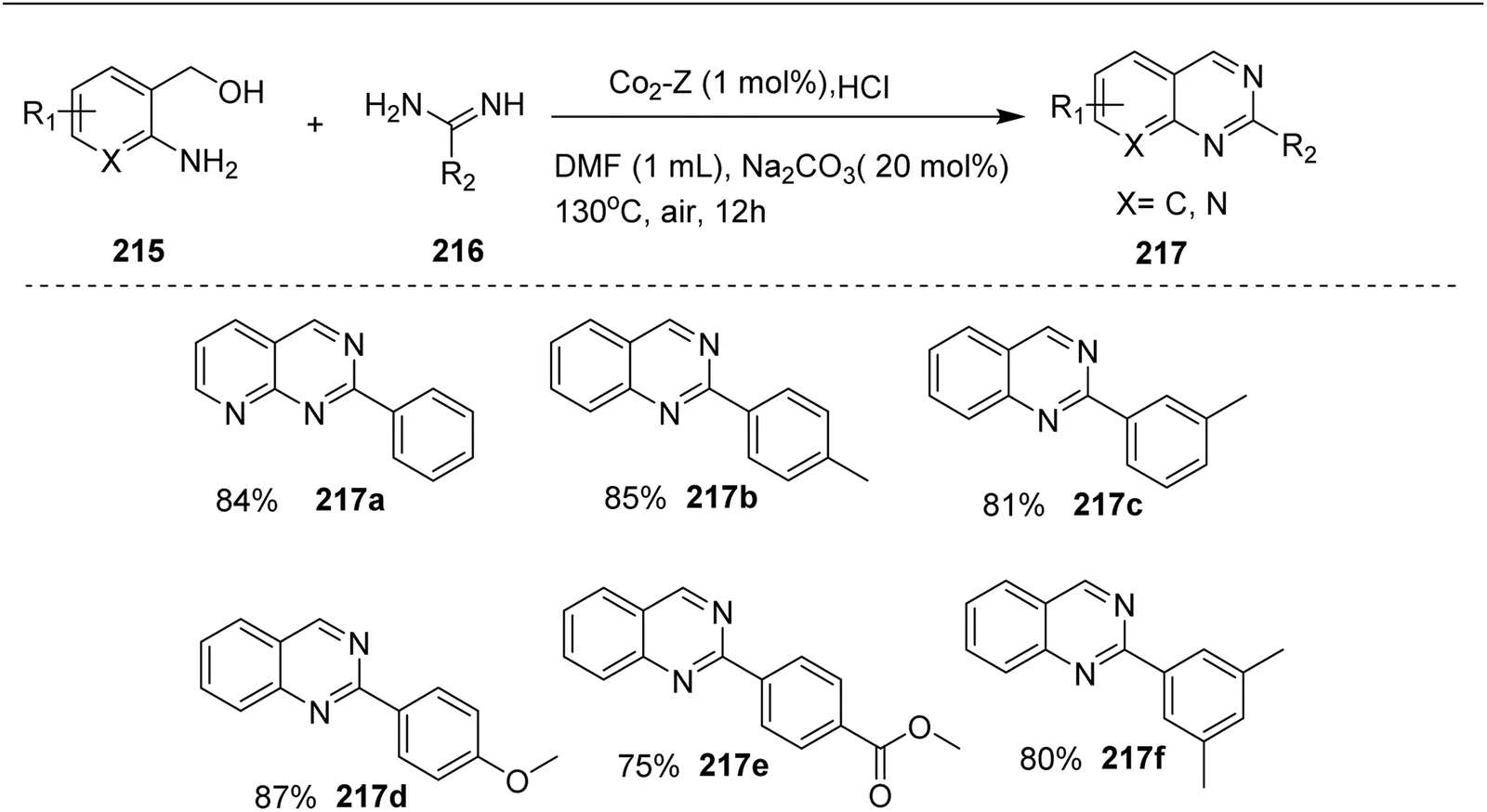
Diatomic cobalt-catalysed cyclization of *o*-amino-benzyl alcohol with amidine.

**Fig. 42:**
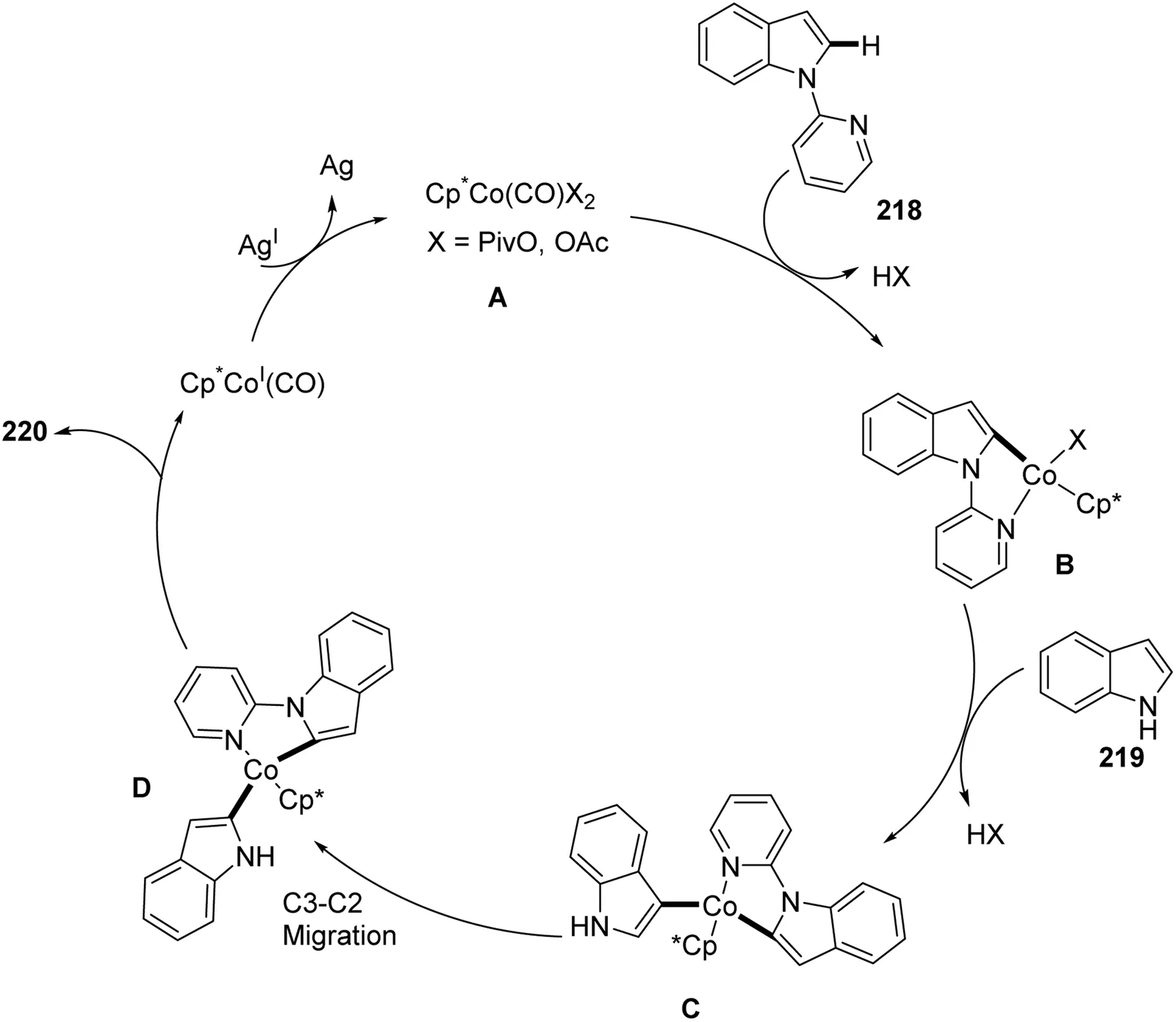
A suitable catalytic pathway.

For the cyclization reaction of anthranilic alcohol 215 and amidine 216, Fu *et al.*, created a heterogeneous cobalt bimetallic catalyst that achieved outstanding catalytic performance, stability, and recyclability in a straightforward and eco-friendly procedure. Under low base loading circumstances, 2-phenylquinazoline 217 and its derivatives were synthesized in a yield of up to 84% *via* cobalt bimetallic catalyst (Scheme 43).^362^ The 2,2′-biindoles are found as significant skeletal components in numerous biologically active substances, agrochemicals and medicines (Fig. 42).^363–367^ It was simple to synthesize 2,2′-biindoles, biologically active indole-carbazole and other alkaloids.^368–371^ For the synthesis of 2,2′-biindoles, a simple and effective technique has been reported using selective Co-catalysed oxidative hetero dimerization of various indoles toward 2,2′-linked bi-indolyl scaffolds.

**Scheme 44:**
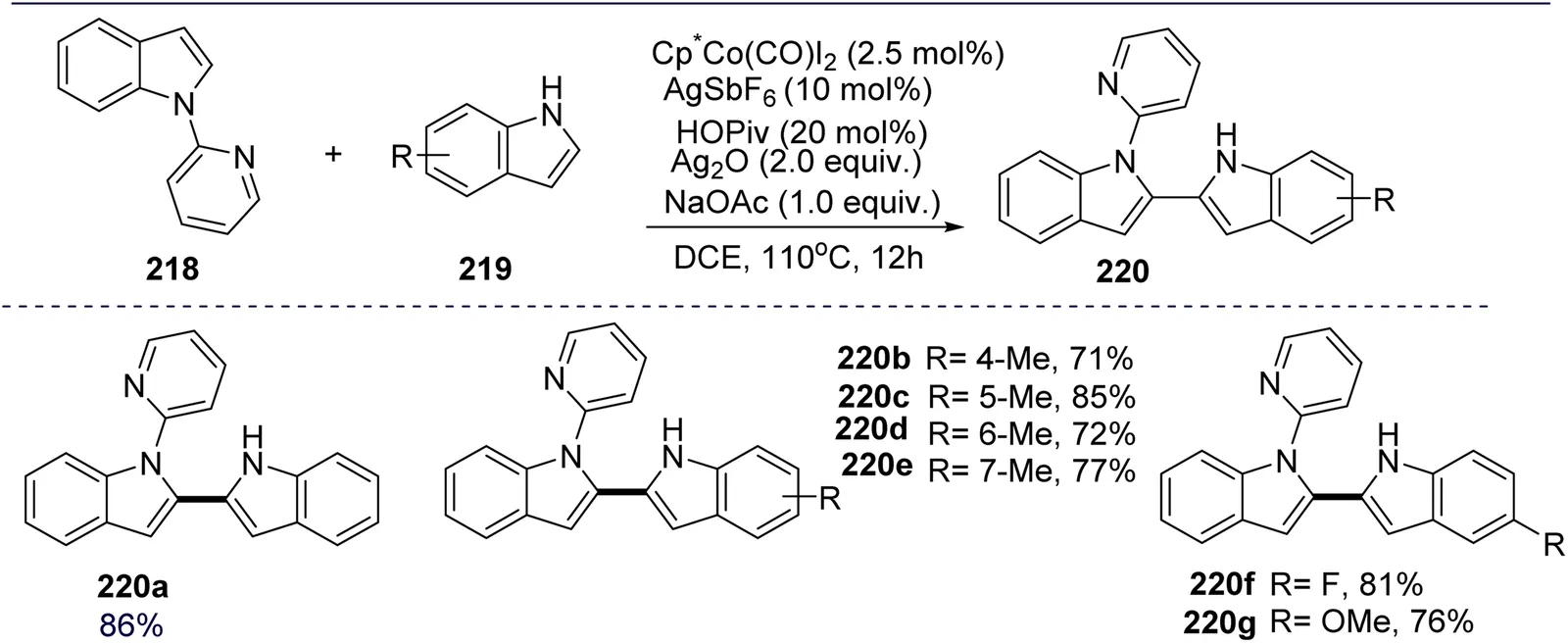
For the synthesis of unsymmetrical 2,2′-biindoles.

Li *et al.*, examined the substrate scope of the reaction under optimum reaction conditions (Scheme 44). The required cross coupling product 220a could initially be produced with *N*-pyridyl indole in an 86% yield. The corresponding unsymmetrical 2,2′-biindoles were produced in good to excellent yields (220b, 220c, 220d and 220e 72–85%) from free indoles involving the –Me group at the C4-, C5-, C6- and C7-positions. Additionally, the corresponding unsymmetrical 2,2′-biindoles 220f and 220g can be produced in moderate to high yields using other free indoles substrates that contain either electron-donating substituents like methoxy or electron-withdrawing groups like Fluorine at the C5- or C6-position. When free indole with a –Me group at the C3-position was utilized, the intended product 220f could not be separated.^372^

In Fig. 42, a catalytic mechanism for the synthesis of 220 has been depicted. Using the substrates 218 and 219 as an example, the coordination of 218 with the Cp*CO(iii) species and the subsequent *ortho*-C–H bond activation of the arene result in the formation of the cyclometallation intermediate B. Then, intermediate C undergoes migration to create intermediate D as a result of the electrophilic interaction that follows between B and the C3-position of the indoles. To get the desired product 220 the resultant intermediate D is then put through a reductive elimination. An Ag salt then re-oxidizes the resultant Cp*CO(i) species to the Cp*CO(iii) species to supply the catalytic cycle.^372^

### 2.13 Hydroalkylation reactions

**Fig. 43:**
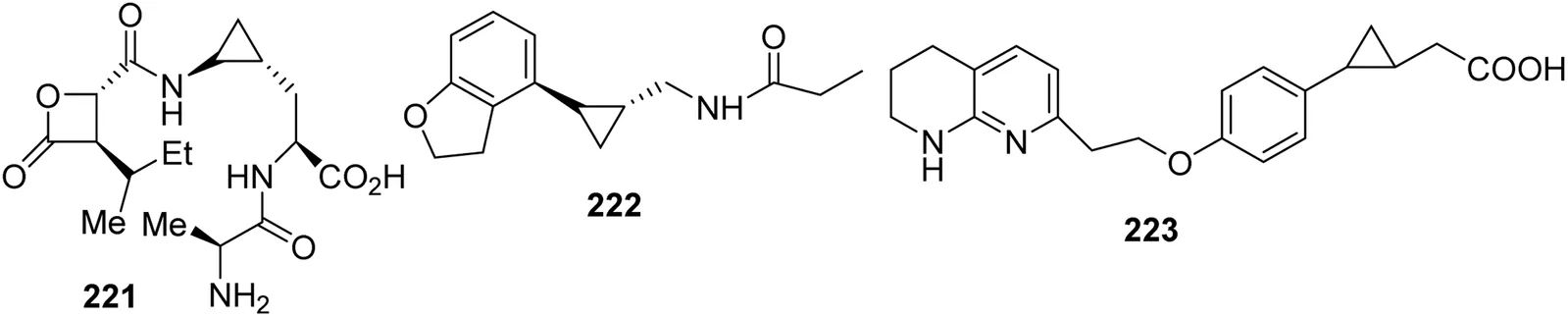
Molecules with biological activity and functionalized alkyl-cyclopropane structures.

Enantioenriched functionalized cyclo-propenes play a key role in many molecules that are biologically active, and they found in a large number of useful molecules used in organic synthesis.^373–376^ Numerous diasteroselective and enantioselective functionalized alkylcyclopropanes intermediates have been prepared for the synthesis of complex molecules and drug development (Fig. 43).^377–379^ The ring-opening of a variety of readily available cyclopropanols and the production of Co-homoenolate intermediates, which are used for the distero- and enantioselective hydroalkylation of cyclo-propenes, were the primary factors for the development of this novel method by the researchers team.^380^

**Scheme 45:**
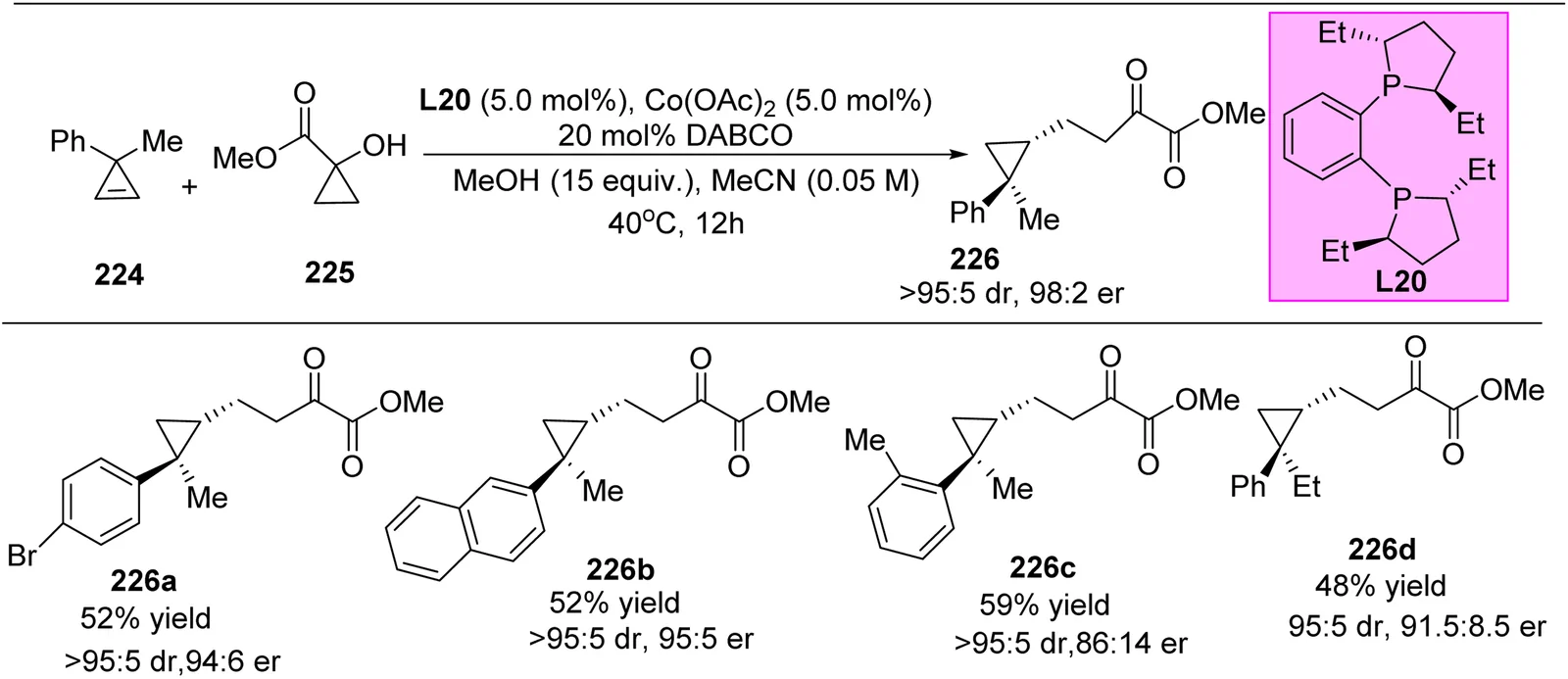
For cyclopropene hydroalkylation reaction, DABCO = 1,4-diazabicyclo[2.2.2]octane.

Huang *et al.*, investigated the substrate range under optimum reaction conditions (Scheme 45). The equivalent products 226a, 226b, 226c, and 226d are produced in this reaction in yields of 52%, 52%, 59% and 48%, respectively, from different cyclopropenes that are appropriate substrates. The reaction efficiencies in this instance are lower than those obtained when using aryl- and alkenyl-substituted cyclo-propanols without a loss in enantioselectivities. The enantioenriched cyclopropane-containing α-ketoesters that are produced may serve as significant precursors for the chiral α-hydroxy- and α-aminoesters that are frequently used in the synthesis of bioactive compounds.^380^

**Fig. 44:**
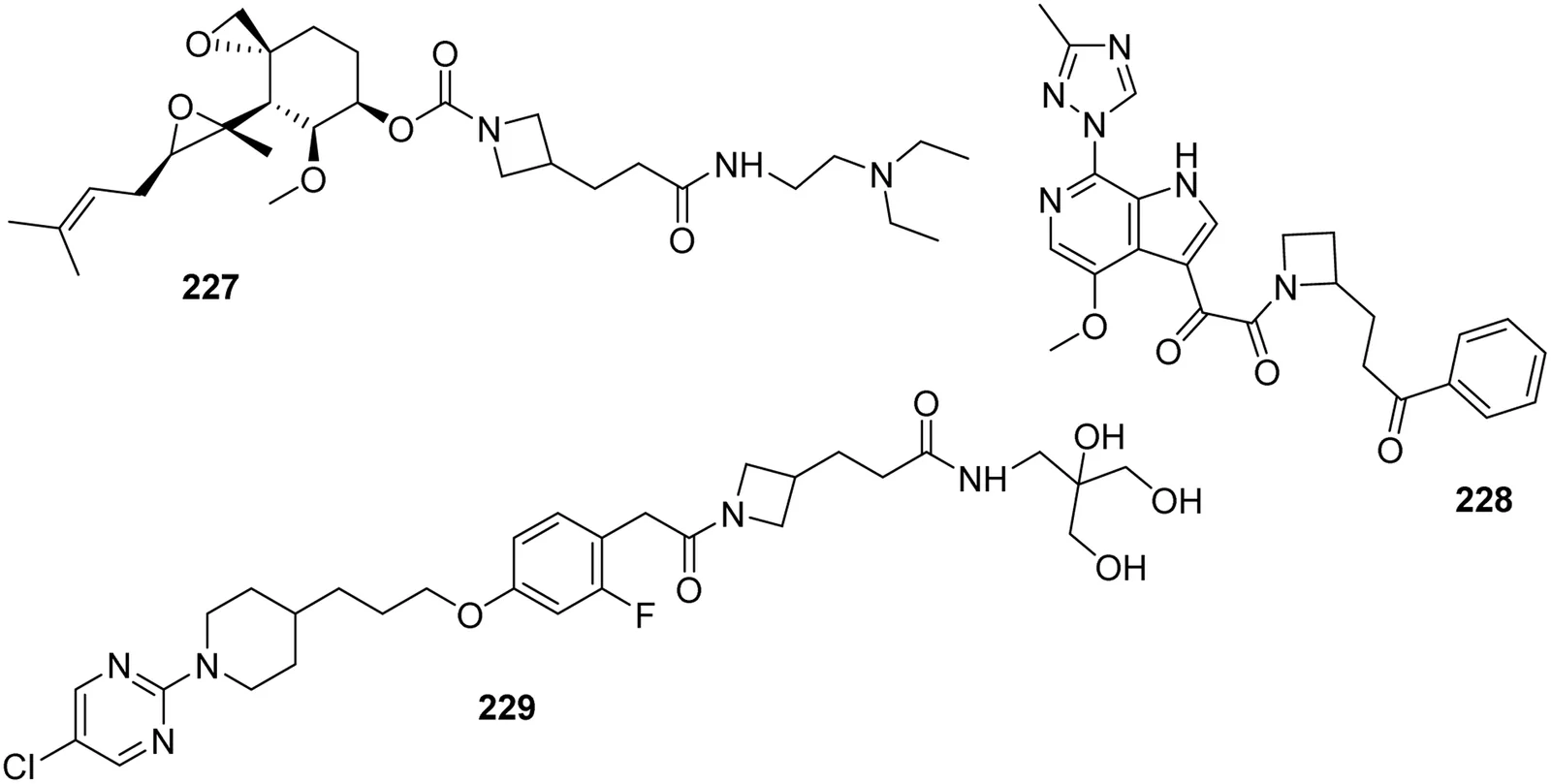
Molecules with functionalized alkyl-azetidine structures that are biologically active.

In addition to being highly valuable synthetic intermediates, azetidines are also frequently identified as structural elements in a wide variety of medications and biologically active compounds (Fig. 44).^381–383^ For instance, molecules containing fumagillol 227 are used to treat obesity.^384^ HIV-1 entry inhibitors 228, in particular HIV-1JR-CSF, demonstrate actions that prevent HIV virus particles from entering human cells.^385^ For the treatment of type 2 diabetes mellitus GPR119 agonists 229 are used. It has been claimed that a unique technique for the highly regioselective hydroalkylation of *N*-Boc-2-azetidines cobalt catalyst exists. The *N*-Boc-2-azetidines may serve as significant precursors to azetidines which are frequently employed in the production of compounds with biological activity.

**Scheme 46:**
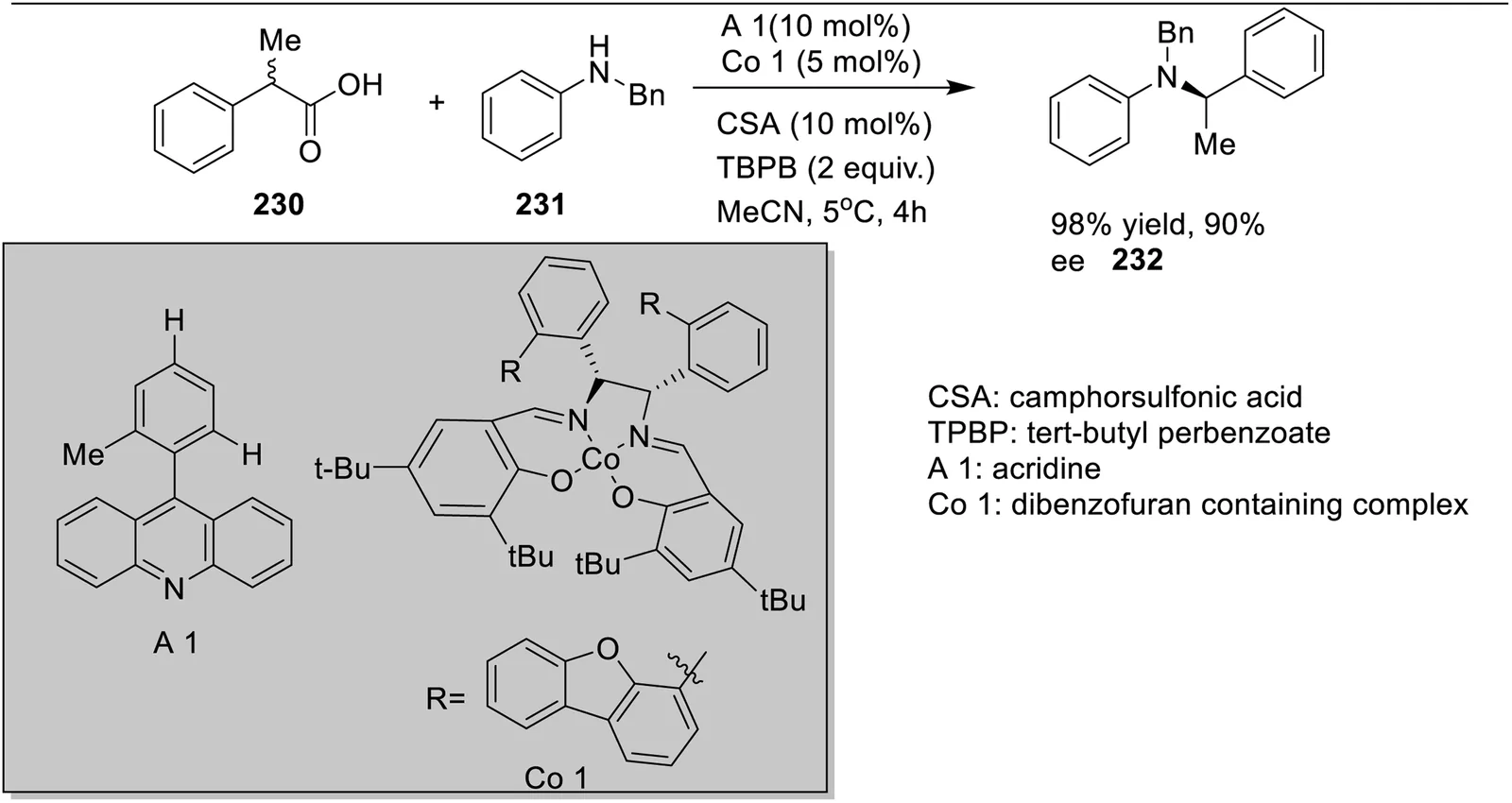
Cobalt-catalyzed synthesis of α-chiral benzylic amines.

A new enantioconvergent decarboxylative *N*-alkylation process of aromatic amines 231 was created by Huang *et al.*, the combination of acridine photocatalysis and cobalt-catalysed radical–polar crossover allows for the creation of asymmetric C–N bonds without preactivating the amine or acid coupling partner. Under mild conditions, the reaction makes it easier to synthesize a variety of α-chiral benzylic amines 232 that are useful both synthetically and medicinally (Scheme 46).^386^

**Scheme 47:**
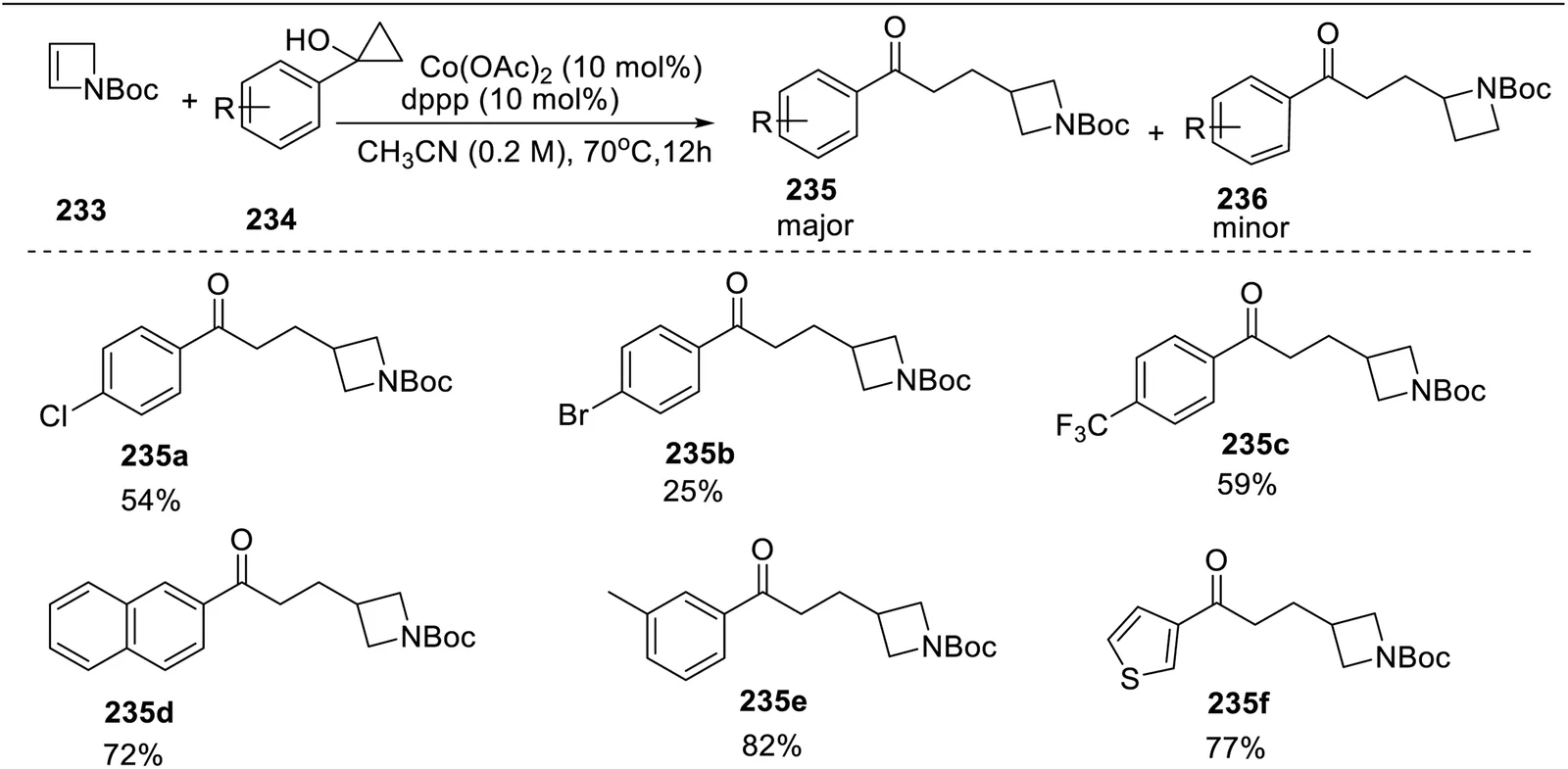
For *N*-Boc-2-azetine hydroalkylation with regioselectivity, dppp = 1,3-bis(diphenylphosphino)propane.

Li *et al.*, examined the substrate scope under optimum reaction conditions (Scheme 47). A variety of azetidines are available from the cyclopropanols with -Ar rings substituted with electron-withdrawing 235a and 235c, electron-donating 235e, sterically congested groups 235d and halogen 235a groups. Due to the catalyst decomposing as a result of adding oxidatively of Co-complex to the C–Br bond, the product 235b is produced in a significantly lower yield than 235a. A 77% yield was obtained during the reaction of cyclopropanols with heterocycle groups 235f.^383^

### 2.14 Multicomponent reactions

**Scheme 48:**
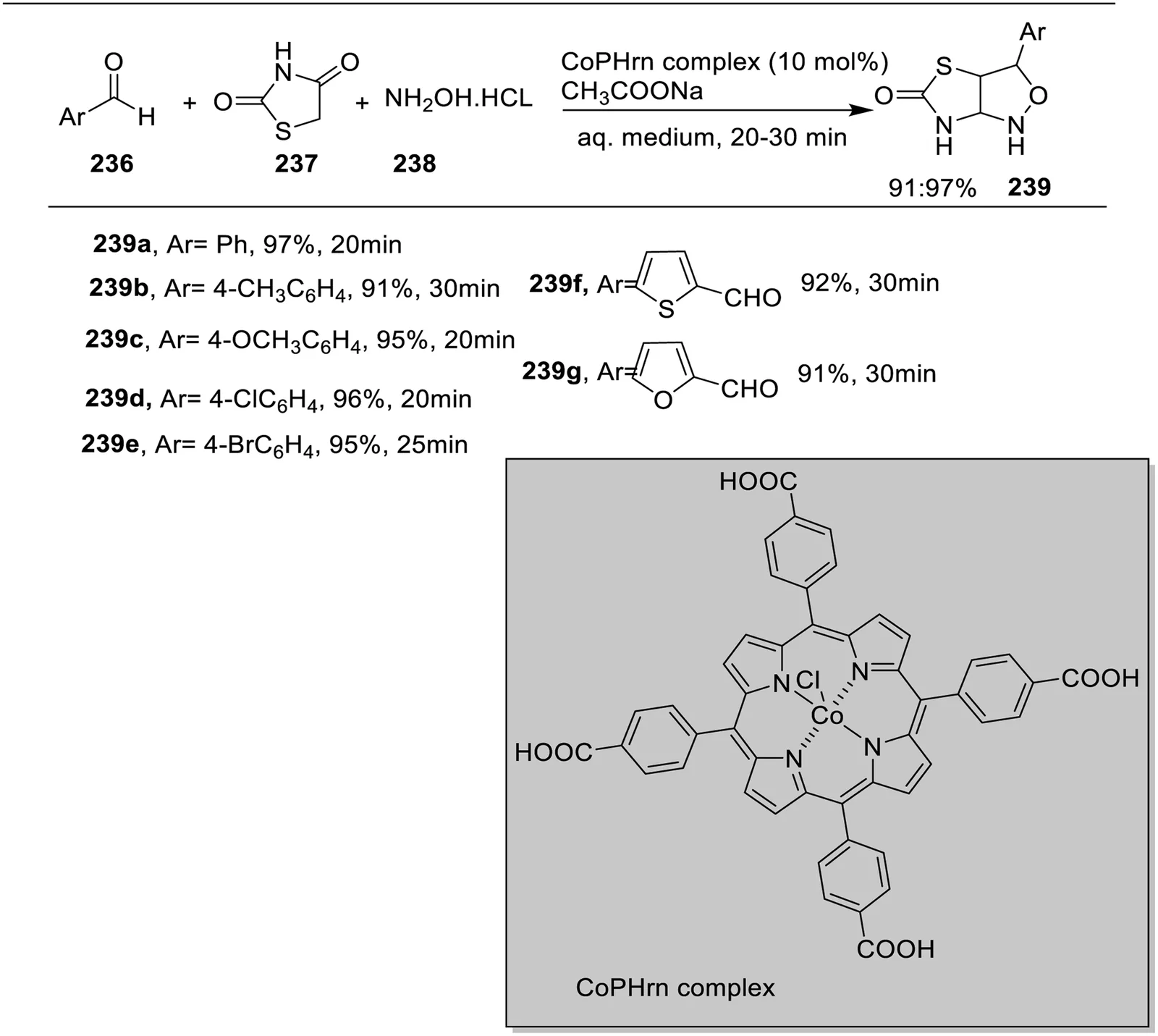
Cobalt-catalysed synthesis of tetrahydro-2-oxa-4-thia-diazapentalen-5-one derivatives.

In order to synthesize tetrahydro-2-oxa-4-thia-diazapentalen-5-one derivatives 239a–g, Abd *et al.*, studied a novel catalytic multicomponent reaction that involved the combination of aromatic aldehyde 236 (1 mmol), 2,4-thiazolidenedione 237 (1 mmol), and hydroxylamine hydrochloride 238 (1 mmol) (Scheme 48). The mechanism of this reaction was examined while taking into account important factors like catalyst amount, solvent selection, reaction time, and the influence of various Lewis acids and basic catalysts on catalytic activity. The goal was to determine the ideal reaction conditions that would produce the desired products with high efficiency. It should be noted that the condensation process would take a lot longer to complete in the absence of a catalyst. In order to boost efficiency and yields of the desired tetrahydro-2-oxa-4-thia-diazapentalen-5-one derivatives, a catalyst is therefore essential to speed up and facilitate the process.^387^

**Scheme 49:**
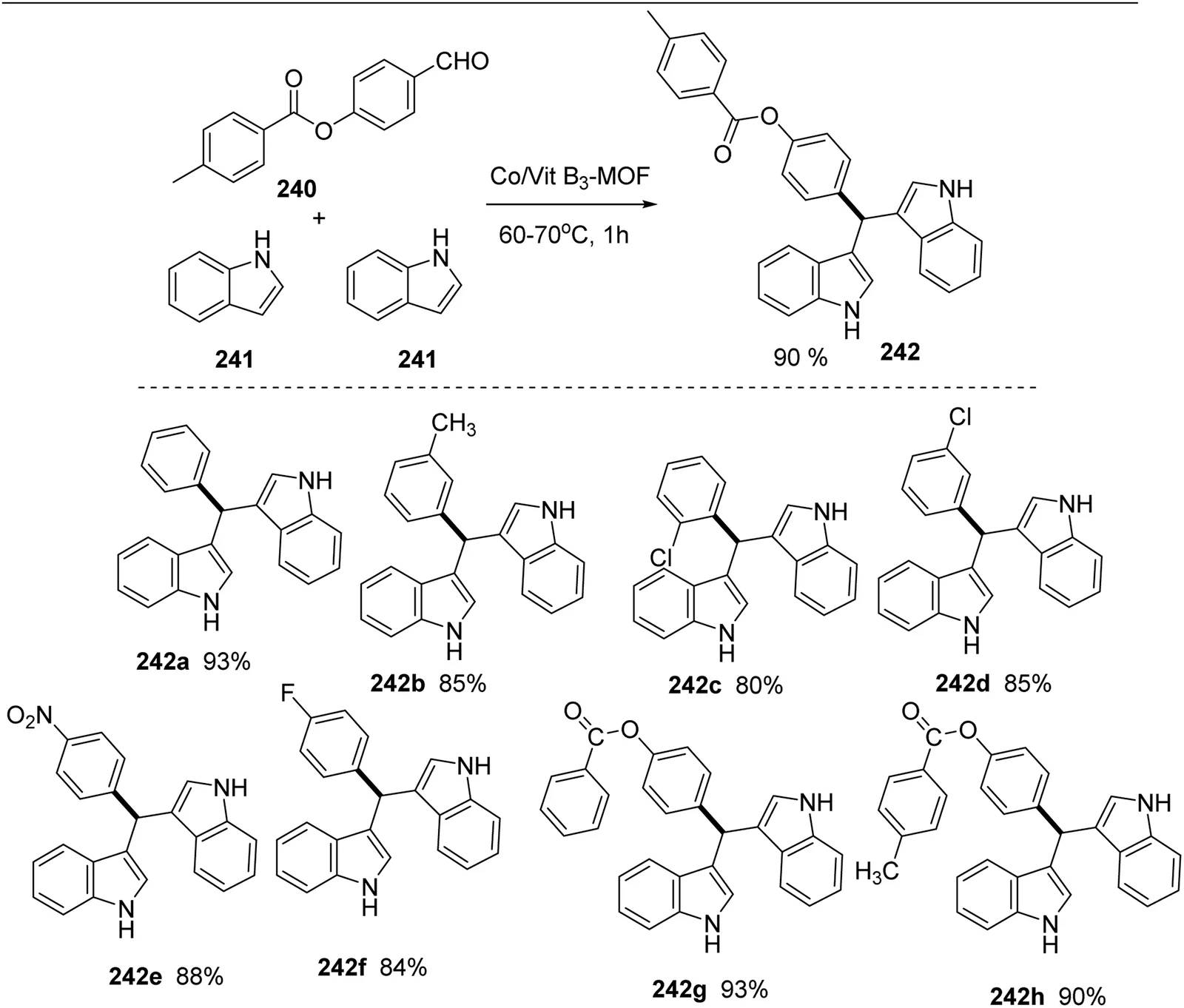
Cobalt-catalysed synthesis of bis-indole derivatives.

**Fig. 45:**
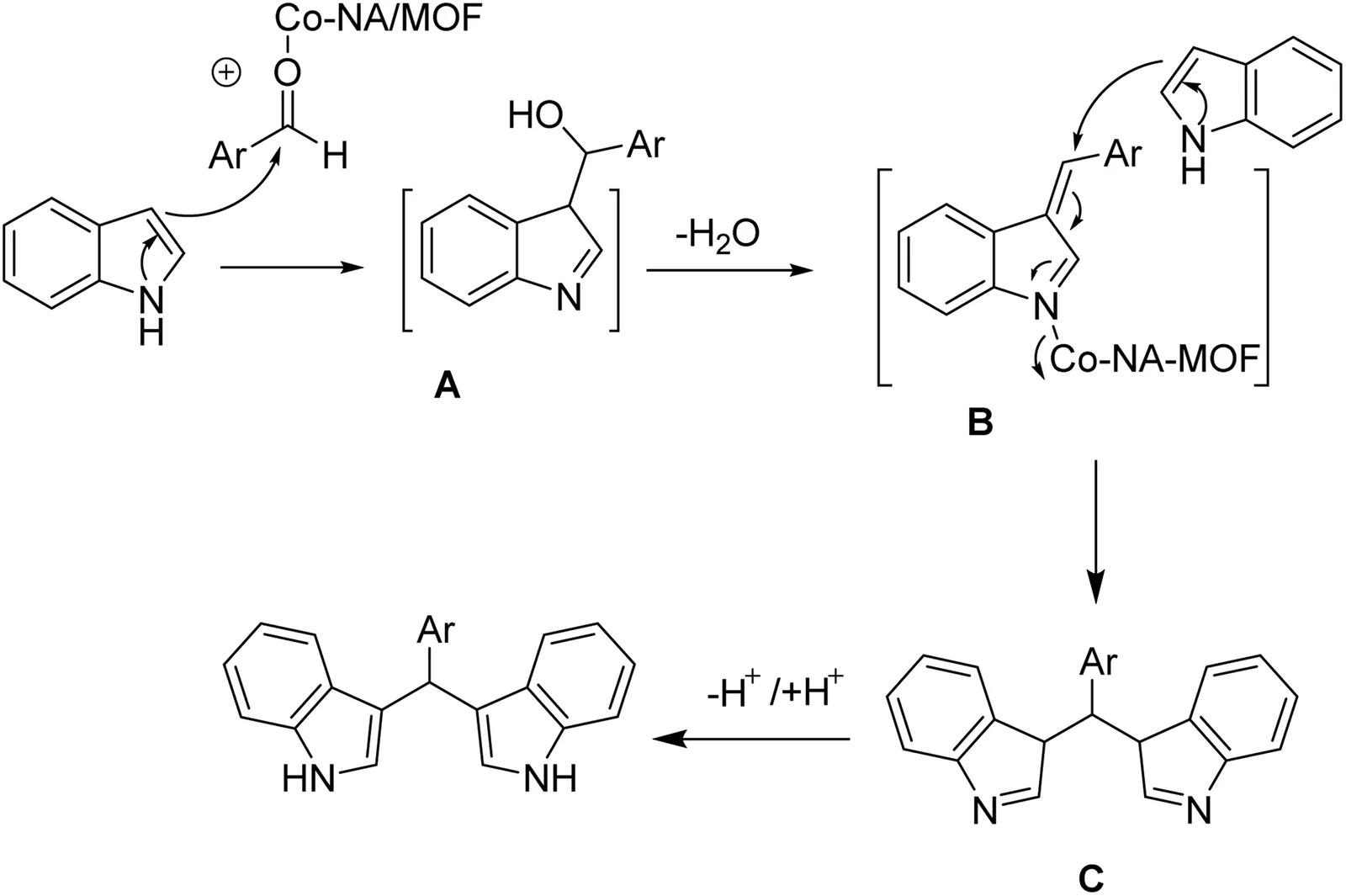
Plausible mechanism for the synthesis of bis-indole.

Under solvent-free conditions, a cobalt/vitamin B3 metal–organic framework was employed as a nano-catalyst in the synthesis of several novel bis-indole derivatives. The extensive porosity and unique surface area in MOFs give exceptional catalytic capabilities, which appeared in the synthesis of indole and its derivatives shown in many biological and natural domains, therefore many researcher groups targeted it and seeking for new synthesis of indoles. Said *et al.*, synthesized bis-indole derivatives *via* one pot synthesis. Two moles of indole 241 and one mole of 4-formylphenyl 4-methylbenzoate 240 were combined with a catalytic amount of the prepared catalyst at 60–70 °C without the use of solvents as an example of the optimized reaction (Scheme 49). Fig. 45 shows the plausible reaction mechanism. By creating an oxonium intermediate and enhancing polarity, the produced Co-MOFs catalysed the aldehydic carbonyl, which attracted the nucleophilic (β) position in indole. The intermediate (I) was created following hydrogen transfer and nucleophilic assault. After dehydration, the catalyst activated the intermediate (II), which attracted the second attack by the (β) position in the second mole of indole, forming intermediate (III). The final product was then obtained after tautomerization.^388^

## 3 Conclusion

In conclusion, research into the use of cobalt metal-based catalysis to create biologically important molecules has opened up a promising avenue for the development of pharmaceuticals and other compounds with biological activity. The study highlights recent advancements in this field that demonstrate the environmental sustainability, efficacy, and adaptability of cobalt catalysis. With its unique reactivity profiles, low toxicity, and affordability, cobalt is a first-row transition metal that makes a good replacement for costlier metals that are frequently employed in catalysis. It has been shown that cobalt catalysis is useful in a variety of synthetic transformations, such as C–H activation, cross-coupling reactions, hydrogenation reactions, reduction reactions, annulation reactions, one pot synthesis, carbonylation reactions, amination reactions and the chiral centre formation process. These techniques not only make it easier to synthesize intricate chemical structures with excellent efficiency and selectivity, but they also encourage the formulation of novel proposals for modifying drug-like compounds. Future developments are expected to broaden the breadth of cobalt-catalysed reactions as this field of study continues to develop, providing new opportunities for the effective and sustainable synthesis of physiologically active chemicals.

## Author contributions

Conceptualization, methodology: M. Z., and F. A.; supervision: M. Z.; writing – original draft preparation: S. S.; writing – review and editing: S. B., R. R. and N. N.

## Conflicts of interest

There are no conflicts to declare.

## Data Availability

No datasets were generated or analysed during the current study.
